# The behavioral and neural basis of empathic blame

**DOI:** 10.1038/s41598-017-05299-9

**Published:** 2017-07-12

**Authors:** Indrajeet Patil, Marta Calò, Federico Fornasier, Fiery Cushman, Giorgia Silani

**Affiliations:** 1 Scuola Internazionale Superiore di Studi Avanzati, Neuroscience Sector, Trieste, Italy; 2 Department of Psychology, Harvard University, Cambridge, USA; 3 University of Trieste, Trieste, Italy; 4 Department of Applied Psychology: Health, Development, Enhancement and Intervention, University of Vienna, Vienna, Austria

## Abstract

Mature moral judgments rely both on a perpetrator’s intent to cause harm, and also on the actual harm caused–even when unintended. Much prior research asks how intent information is represented neurally, but little asks how even unintended harms influence judgment. We interrogate the psychological and neural basis of this process, focusing especially on the role of empathy for the victim of a harmful act. Using fMRI, we found that the ‘empathy for pain’ network was involved in encoding harmful outcomes and integrating harmfulness information for different types of moral judgments, and individual differences in the extent to which this network was active during encoding and integration of harmfulness information determined severity of moral judgments. Additionally, activity in the network was down-regulated for acceptability, but not blame, judgments for accidental harm condition, suggesting that these two types of moral evaluations are neurobiologically dissociable. These results support a model of “empathic blame”, whereby the perceived suffering of a victim colors moral judgment of an accidental harmdoer.

## Introduction

Law, philosophy and psychology agree on a basic two-part template for moral judgment. On the one hand, we judge actions by considering the mental states that prompted them—for instance, a malicious motive or a misguided belief^1–3^. On the other hand, we also judge actions by assessing whether they actually caused harm, and how much^1, 4, 5^ Combining these features, if we see a person fire a gun, we naturally ask, “why did she shoot”? and “did she hit anyone”? before passing judgment.

The contribution of mental state analysis to moral judgment is well studied. Past research identifies the brain networks responsible for mental state analysis in moral judgment^6–12^, characteristic deficits in clinical populations^13, 14^, and so forth^15^. Indeed, the synthesis of behavioral and neural research on mental state analysis in moral judgment stands out as a remarkable case study of integrative social cognitive neuroscience.

In contrast, far less research interrogates the behavioral and neural basis of the second major contributor to moral judgment: Representations of the actual harm caused by an act^16–18^. These play a crucial role in “moral luck”, the phenomenon of chance outcomes influencing moral judgments^1, 19–22^. (For instance, two drunk drivers who fall asleep at the wheel face very different penalties if one runs into a tree and another runs into a person). Although chance outcomes only play a small role in adults’ moral judgments of a person’s character or conduct (i.e., *acceptability judgments*), they play a large role in the assignment of blame and punishment^1, 4, 23, 24^ (i.e., *blame judgments*) (for a more detailed discussion, see Supplementary Text S1). We aim to clarify the psychological and neural basis of this effect.

Existing behavioral research hints that a key feature of harm representation is our capacity for empathy with a victim^18, 25–31^. Below, we report new behavioral findings that support and refine the “empathic blame” hypothesis, which states that the degree to which we condemn others for producing harmful outcomes, intentionally or unintentionally, depends on the degree to which we empathize with the victim’s suffering. We then turn to our principle aim, which is to interrogate its neural basis. Specifically, we ask whether, and how, a network of brain regions associated with empathy for the victim contribute to the moral judgment of third party actions.

In order to accomplish this, we build on prior research^32^ showing that a specific network of brain regions is activated when we witness others suffer, referred to here as the “empathy network”. These brain regions overlap partially with the brain regions that are activated when we experience the sensation of pain ourselves, indicating that they may enable empathy for suffering (i.e., a congruent emotional response in an agent and an observer^33^). (We refer to this set of neural structures as the “empathy network” not because it is activated exclusively by empathy–indeed, it certainly is not^34^–but rather because it responds reliably in the presence of empathy for the suffering of others^35^). Past research shows that the activation of this network plays a role in promoting prosocial action aimed at alleviating victim suffering^36–40^. Yet, at the neural level, it has not previously been associated with the moral condemnation of those who cause others to suffer^41^.

Following prior research into the neural basis of moral judgment^2, 7, 8, 17^, we presented participants with vignettes describing third-party actions that varied both in their intent and their outcome towards a potential victim. For instance, a person might put a white powder they believe to be sugar (neutral intent) or poison (harmful intent) into a potential victim’s coffee, and the powder might be sugar (neutral outcome) or poison, causing the victim to die (harmful outcome).

We use these cases to test three novel predictions. First, we predict that the empathy network will show enhanced activation at the time when the outcome to a victim is described, compared with other periods of the stimulus presentation when no information about harm is provided. This response might be selective to cases in which harm occurs, or it might generalize even to cases in which harm does not occur, indicating a reliance on affective mirroring to process both harmful and benign outcomes. In either event, this prediction is consistent with the hypothesis that empathy underlies the moral sensitivity to the experiences of the victim of a potential transgression. However, it can also be seen as consistent with the possibility that people empathize with victims in a manner disconnected from their moral judgment of the harmdoer.

Thus, more specifically, we predict a positive correlation between activation of the empathy network when delivering their moral judgments–presumably reflecting the influence of empathy for the victim upon moral judgment–and condemnation of actions that result in harm. In contrast, we predict no such correlation for malicious acts that happen not to produce harm; for instance, when a person pours what they believe to be poison into a person’s coffee, but the powder turns out to be harmless sugar. In other words, we expect that the more one empathizes with the suffering victim, the more one is likely to condemn the perpetrator’s action, but only for actions that result in harm.

Finally, we anticipate that enhanced activation of the empathy network during moral judgment will be moderated by the category of judgment, i.e. whether acceptability or blame judgment needs to be made. As noted above, moral luck (i.e., moral judgments that are sensitive even to chance outcomes) is substantially stronger for blame judgments than for acceptability judgments. In other words, even when peoples’ behavior is judged morally acceptable, they will be blamed for accidental harms that arise from it. Consequently, we predicted greater activation of the empathy network during judgments of blame (which rely more on the assessment of harm) than during judgments of acceptability (which rely less on the assessment of harm). This final prediction allows for an especially strong inference that activation of the empathy network subserves the task of *moral judgment*, rather than arising as an incidental byproduct of the stimuli.

In summary, we hypothesize that empathy for victims contributes to the condemnation of harm and, therefore, that (1) neural activation consistent with affective sharing will occur when learning about outcomes to victims, (2) especially among people whose moral judgments are most sensitive to outcomes, and (3) especially for types of judgment that are most sensitive to outcomes.

## Behavioral Studies

We begin by presenting the results of eight preliminary behavioral studies that motivate our neural investigation. These studies do not each precisely match our imaging study in terms of the content of the stimuli or manner of presentation, nor were they designed to. Rather, they establish a theoretical background against which our neuroimaging design, analysis, and interpretation can best be understood.

## Studies (1–4): Empathy predicts the condemnation of accidents

**Figure 1:**
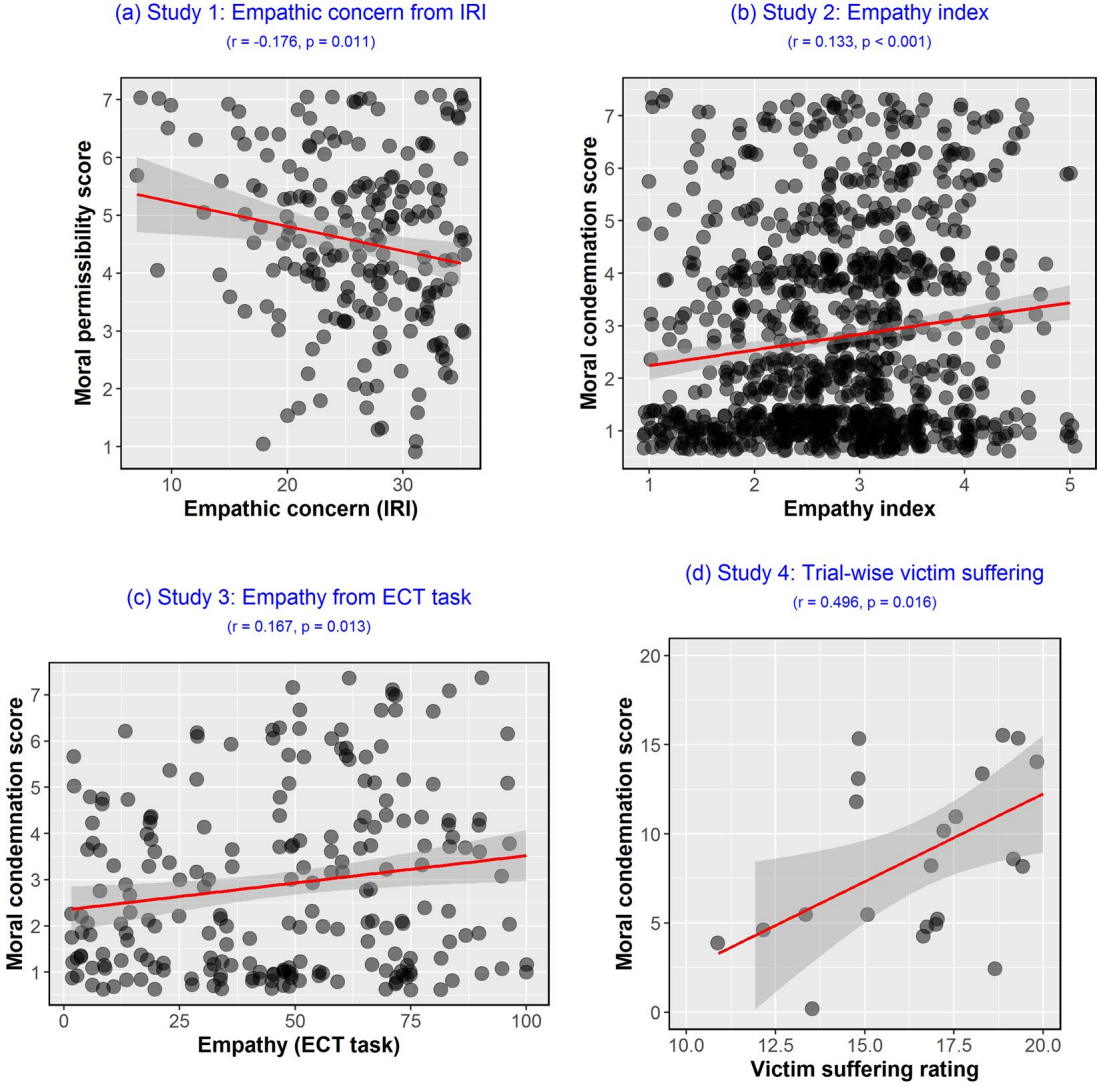
Positive relationship between empathy and condemnation of accidents. Behavioral studies 1–4 revealed that individuals who scored high on empathy, as assessed using (**a**) empathic concern (EC) subscale of Interpersonal Reactivity Index (IRI), (**b**) Empathy index measure, (**c**) empathy scores from the Empathy-Compassion Task (ECT), and (**d**) trial-by-trial victim suffering ratings, condemned accidental harm-doers more severely. Different questions were asked across studies 1–4: (**a**) moral permissibility, (**b**,**c**) wrongness and punishment, and (**d**) acceptability and blame.

The empathic blame hypothesis posits that we condemn actions based on the harm they cause because we empathize with the victim. This effect would be especially crucial for the condemnation of accidental harms, as compared to intentional harms. This is because intentional harms can be condemned for another salient reason: the agent’s malicious intent. In contrast, accidental harms are condemned solely based on harmful outcomes (perhaps due to empathy, as we hypothesize). A strong prediction, then, is measure of empathy will correlate with measure of moral condemnation for accidents. We tested this prediction across four studies that utilized several trait and state measures of empathy (Fig. 1; full details in Supplementary Text S2–S5).

In Study 1, we found that participants scoring high on dispositional empathic concern (EC, assessed with Interpersonal Reactivity Index or IRI^42^) judged accidental harms to be *less* morally permissible (*r* = −0.177, 95% CI [−0.306, −0.042], *t*(205) = −2.574, *p* = 0.011). We focused on EC subscale based on prior work showing that higher EC is associated with more severe assessment of accidental harms^27, 29^. In Study 2, we replicated this effect using another measure of trait empathy (Empathy index^43^) and found a positive association between higher self-report scores of empathy and condemnation of accidents (*r* = 0.133, 95% CI [0.072, 0.193], *t*(1002) = 4.234, *p < *0.001). In Study 3, we borrowed a recently developed measure designed to dissociate empathy from compassion^30^ and found that there was a positive correlation between empathy scores and severity of judgments for accidents (*r* = 0.167, 95% CI [0.036, 0.293], *t*(218) = 2.506, *p* = 0.013). In Study 4, we found that trial-by-trial ratings of perceived victim suffering for each moral vignette predicted harsher moral condemnation only for the accidental harms (*r* = 0.496, 95% CI [0.106, 0.754], *t*(21) = 2.618, *p* = 0.016). *Note*: Only simple linear correlations are shown here for illustrative purposes. To see results from ordinal mixed-effects regression analyses﻿ for these studies, see Supplementary Text S2–S5).

In summary, these studies supported the empathic blame hypothesis: individuals that either self-reported to have more empathic predisposition or reported to have stronger situational empathic engagement gave more severe ratings for agents who unintentionally caused harm. We also note that all effect sizes were small to typical, according to contemporary guidelines^44^.

## Study 5: People empathize with victims more than perpetrators

**Figure 2:**
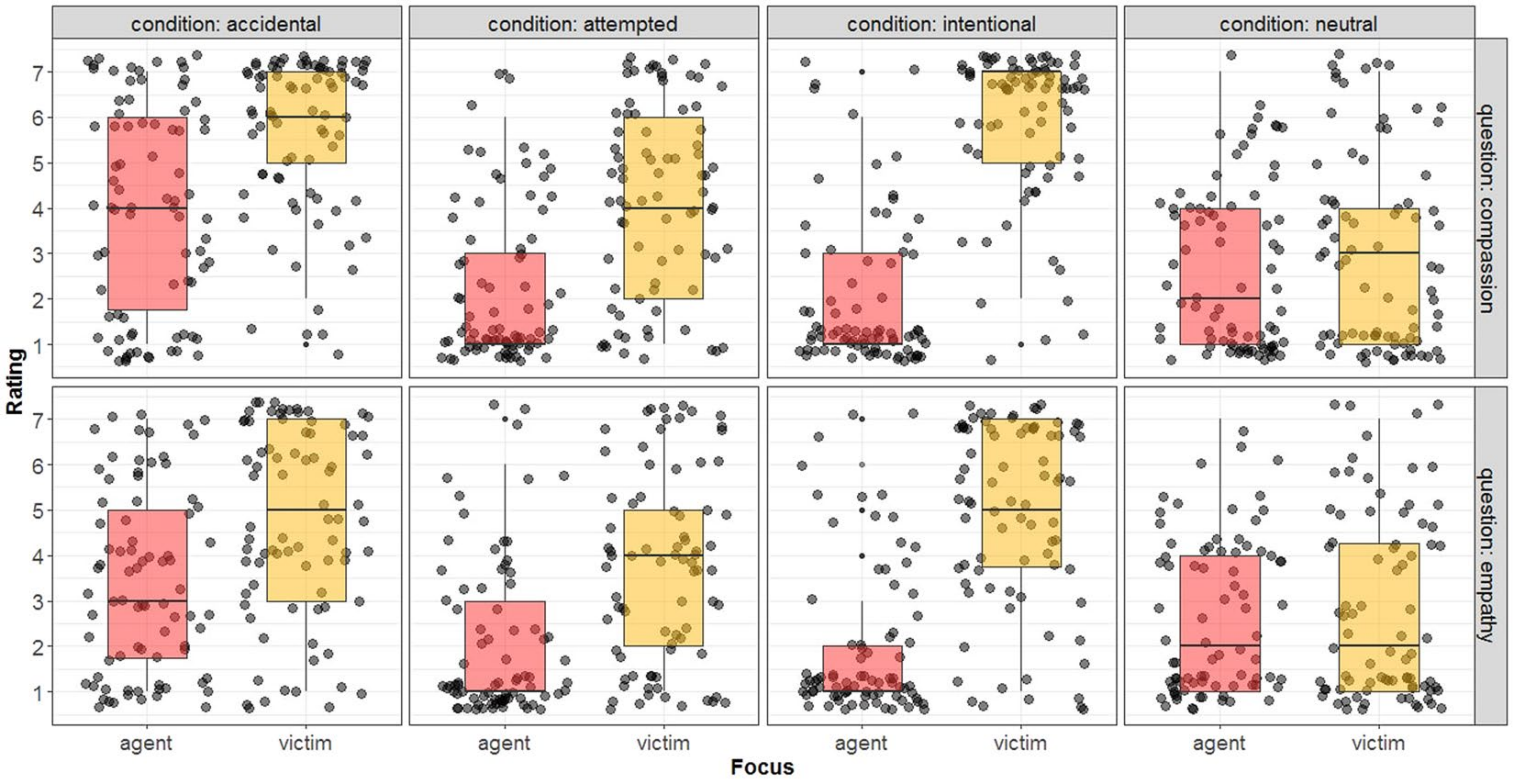
Empathy is for the victims of harm, not the agent. Boxplots with jittered data-points represent participants’ trial-by-trial ratings for empathy (affective sharing, i.e.) and compassion questions revealed that they empathized with and felt compassion for the victim, and not the agent, of the third-party dyad. Participants did report that they felt some empathy and compassion for agents involved in accidental harms.

The preceding studies tacitly assumed that the relevant target of empathy in a moral vignette is the victim. In theory, however, unless properly tested, we cannot rule out the possibility that people are empathizing with the *perpetrator* of the harmful accident (“Oh dear, he must feel awful about what happened!”). The positive association found between assessment of victim suffering and blame for the agent speaks against this alternative, since it is difficult to see how sharing the internal states of the accidental harmdoer–which are blameless–would *increase* the severity of moral judgment. In Study 5 we test of this inference by directly asking people who they empathized with. Across all conditions of our design we find greater empathy for victims than agents (*p*s < 0.001; see Supplementary Text S6), although accidents do tend to elicit the greatest degree of empathy for agents, relative to the other conditions (Fig. 2).

## Studies 6–8: Punishment shows greater outcome effects than acceptability

**Figure 3:**
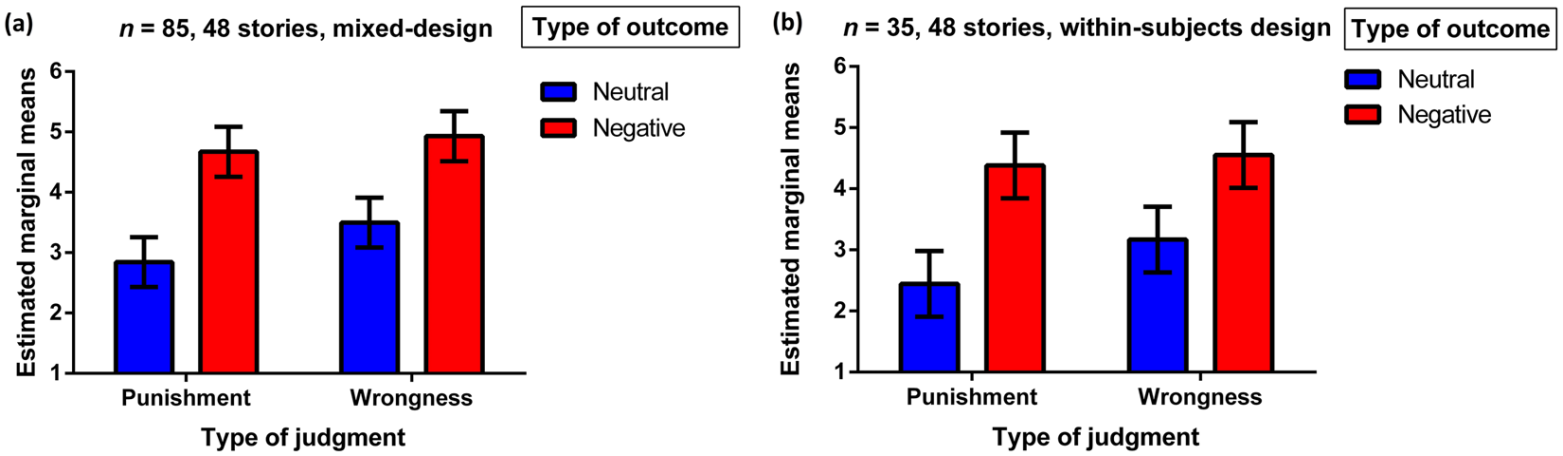
Increased reliance on outcomes for punishment versus wrongness judgments. The outcome-by-judgment interaction effect was observed in (**a**) a mixed-design and (**b**) a within-subjects design such that the difference in ratings for negative versus neutral outcomes was greater for punishment judgments as compared to wrongness judgments. Error bars represent 95% confidence intervals.

As we have already discussed, prior research shows that judgments of moral blame show greater dependence on the harm caused to a victim than do judgments of moral acceptability^1, 23, 24, 45^. This effect is crucial to our neural analyses, and so we first successfully replicated it (Fig. 3)–both in a within-subjects design (similar, but not identical, to our neuroimaging paradigm) in Study 6, and then in a mixed-design (more similar to prior research) in Study 7 (*p*s < 0.001; see Supplementary Text S7 and 8 for full details).

**Figure 4:**
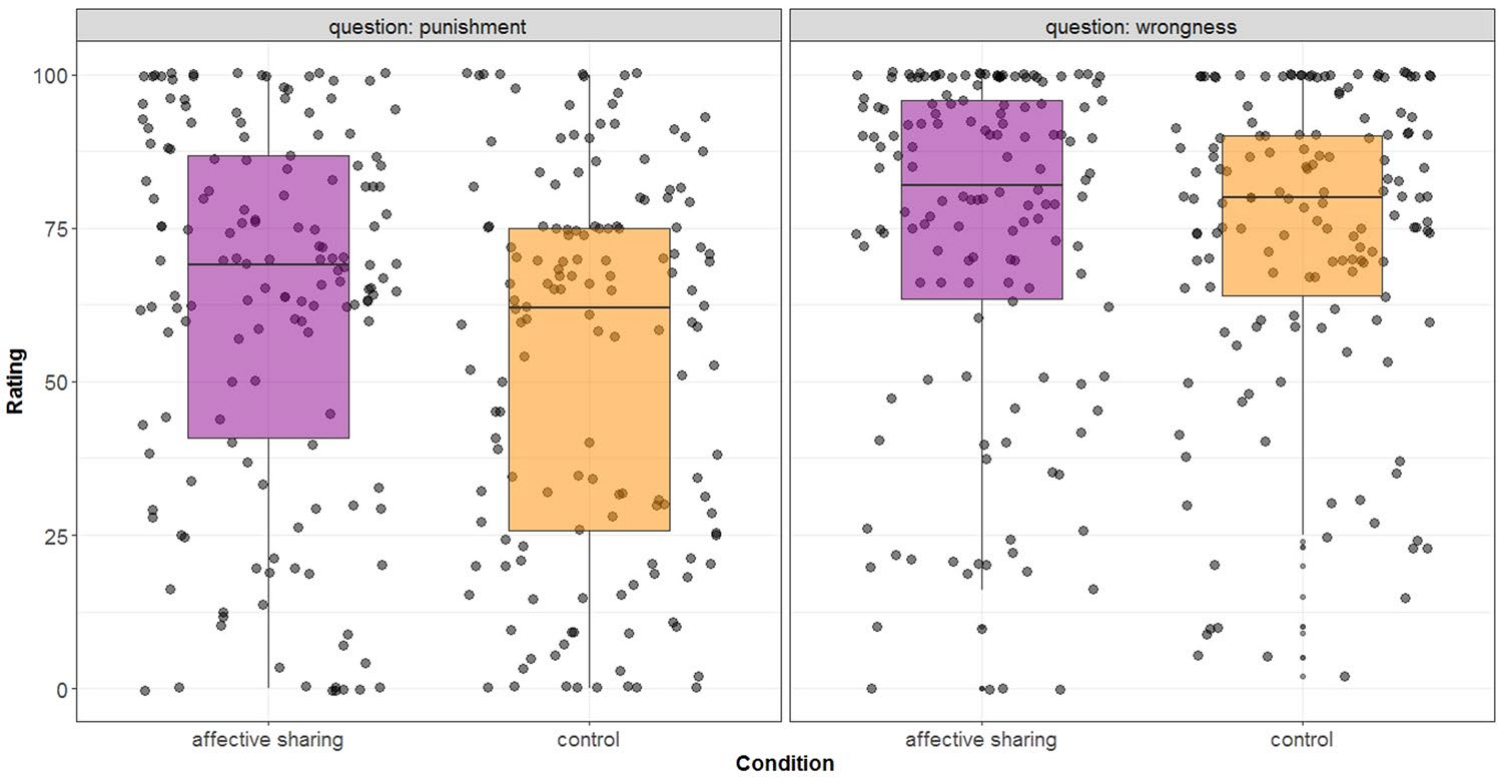
Instructional affective sharing increases punishment for accidents. Boxplots with jittered data-points show participants’ wrongness and punishment judgments in two conditions: when they were instructed to share the victim’s pain (affective sharing group) and when no such instruction was provided (control group). Instructional affective sharing increased punishment ratings, but left wrongness judgments unaltered.

To provide further convergent evidence, we conducted an additional study (Study 8) in which we explicitly instructed participants to share the victim’s pain (vs. no instructions) and then provide either punishment or wrongness judgment. Again, our focus was cases of accidental harm. As expected, when instructed to share victim’s pain, participants punished the accidental harm-doer more (Fig. 4), but their wrongness judgments remained unaffected (interaction effect: *p* = 0.06; full details in Supplementary Text S9).

## fMRI Study (Study 9)

Having established the effects of interest at the behavioral level, we next turned to investigating the neural basis of empathic blame. We investigated if harmful outcomes are encoded and retrieved via the empathy network and whether this information is recruited differentially for different types of moral judgments.

## Methods and Materials

### Participants

We tested 50 healthy community members (32 females; average age 23.06, SD = 3.08). We excluded all data from one participant because we discovered after testing that he had consumed clinically-prescribed psychoactive drugs. Functional data from two participants was removed due to excessive head motion, from four participants due to high collinearity among regressors (see below), and data from one additional participant could not be collected due to technical error. Thus, valid functional data was available for 42 participants, while behavioral data was available for 49 participants.

### Experimental stimuli and procedure

#### Moral judgment task

Participants read and responded to 36 moral scenarios adapted from prior studies^1, 6^ and translated into Italian (see Supplementary Text S10 for more details). Across participants, each scenario appeared in four variations derived from a 2 × 2 design crossing *belief* (neutral, negative) and *outcome* (neutral, negative). Each participant viewed 9 scenarios from each cell of this design.

**Figure 5:**
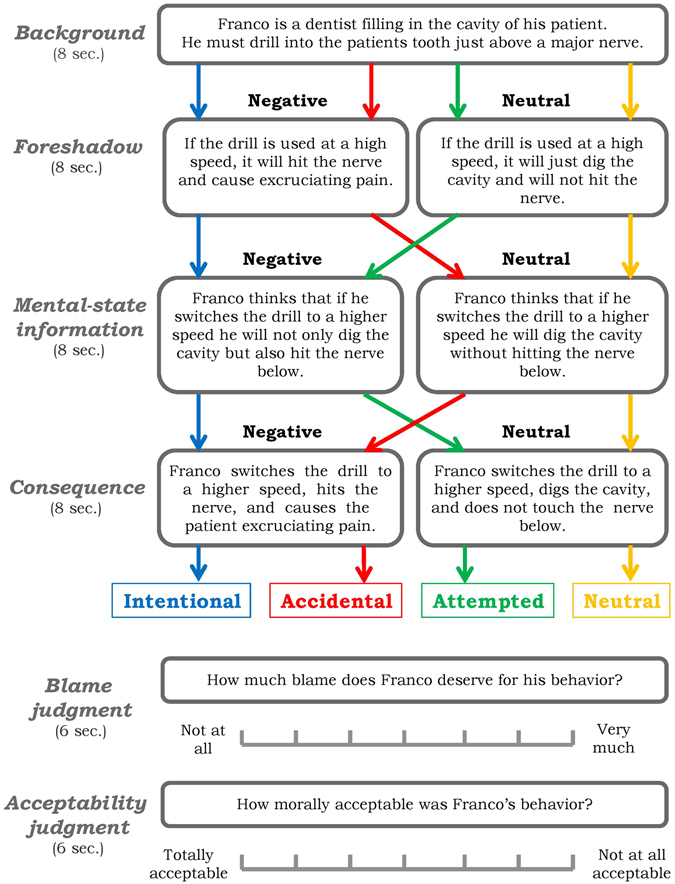
Experimental stimuli and design. Each moral vignette consisted of the following text segments: a *background* stem providing set-up for the story, a *foreshadow* segment that foreshadowed the nature of outcome, a *mental-state information* segment that provided information about actor’s belief, a *consequence* segment that described action and its outcome. These segments were then followed by questions assessing acceptability and blame judgments.

Each scenario lasted for 32 s and consisted of four cumulative segments (each lasting for 8 s): (*i*) *background:* this stem was common to all variations and provided settings in which the story took place; (*ii*) *foreshadow:* this segment foreshadowed whether the outcome will be neutral or harmful; (*iii*) *mental-state information:* this segment provided information about whether the agent was acting with a neutral or harmful belief; (*iv*) *consequence:* this final segment described agent’s action and its outcome. We use the terms *mental-state information* instead of *belief*, and *consequence* instead of *outcome*, to avoid confusion: the latter terms represent factors of the experimental design, while the former terms represent story segments containing information about the agent’s beliefs and the nature of the outcome, respectively. All story text was then removed and replaced with the question and response scale (see Fig. 5).

After reading each story, participants provided two types of moral judgments^1^ (presented in randomized order) on all trials:

- *acceptability*–“How morally acceptable was [the agent]’s behavior”? (1: *Totally acceptable* to 7: *Not at all acceptable*);
- *blame*–“How much blame does [the agent] deserve”? (1: *Not at all* to 7: *Very much*).

Note that although the online behavioral studies (6–7) included the terms “wrongness” and “punishment,” the fMRI study used the terms “acceptability” and “blame”. This is because the surveys were conducted in English with US participants, while the fMRI study was conducted in Italy and the scenarios were presented in Italian and introduced two additional concerns- (*i*) there is no direct translation of “wrongness” in Italian and the most natural analogue is “acceptability”; (*ii*) participants seem to adopt an internal punishment scale based on a legal metric of incarceration duration when the term “punishment” is used, even in the absence of explicit instructions to do so^46^, and we were not certain whether this would be the case with Italian participants and thus used the term “blame”. Crucially, prior research shows similar patterns of reliance on harm caused for both blame judgments and punishment judgments^1^. Indeed, the effect for punishment has been replicated across numerous studies^1, 4, 23, 24^.

Each question lasted for 6 s and participants could provide their judgment using a 7-point Likert scale on which cursor could be moved using two fingers; the initial location of the cursor was randomized across trials. After each scenario, participants viewed a fixation cross on the screen for a jittered ITI of 2–4 seconds. Additional details about the experimental protocol are provided in Supplementary Text S11.

In summary, the six segments of interest could be divided into two phases: four *reading* phase segments (*background*, *foreshadow*, *mental-state information, consequence*), when initial representations were formed, and *judgment* phase segments (*acceptability* and *blame*), when the encoded information was recruited in the service of making moral judgments.

#### Functional localizer task

Empathy involves the ability to understand and share others’ affective states (emotions, pain, etc.) in isomorphic manner while maintaining self-other distinction^32^. It has been consistently shown that perceiving others in pain (empathy for pain) activates a set of brain regions that also encode nociceptive information while one is experiencing pain first-hand^32, 35, 47, 48^, the so-called “empathy network”.

**Figure 6:**
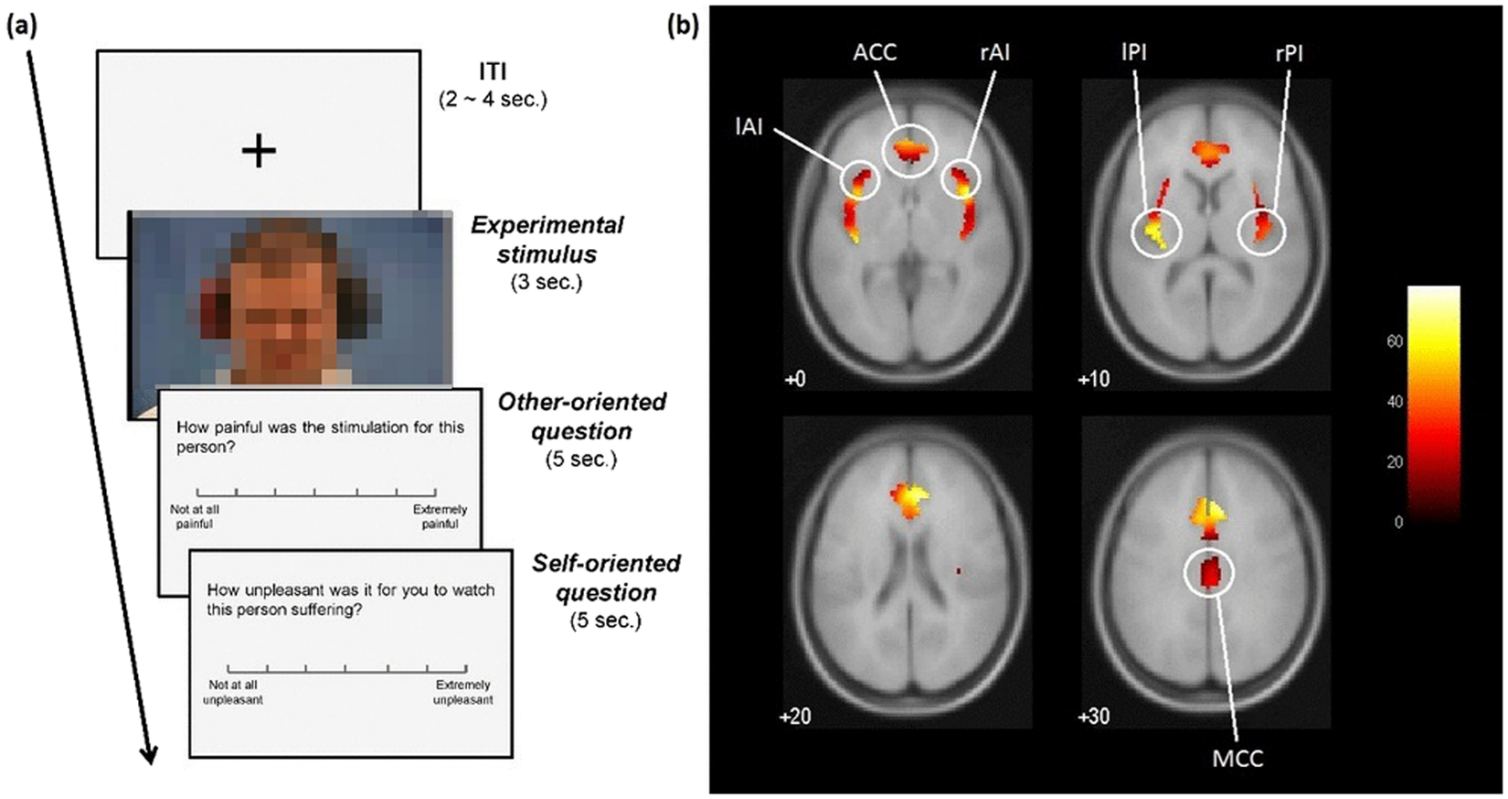
Task schematics and results for the empathy localizer task. (**a**) The videos showed individuals receiving auditory stimulation and displaying the transition from neutral facial expression (0.5 s) to exhibiting painful facial expressions (2.5 s). After presentation of each painful video, participants responded to two questions: one assessing *other-oriented* empathic response by gauging intensity of the experienced pain, while the other assessing *self-oriented* distress via experienced unpleasantness. The face of the target has been pixelated to protect the identity of the person in photo. (**b**) Brain regions where the BOLD signal was higher while watching painful videos as compared to baseline (*n* = 49, group-level random effects analysis, *p* < 0.05, FWE-corrected, *k* > 10), masked with Neuromorphometrics anatomical atlas labels for ROIs. The slice numbers represent *z*-coordinate in MNI stereotactic space and the color bar denotes the *F*-statistic. Regions of Interest (ROIs) are highlighted and labeled. *Abbreviations*: ACC: anterior cingulate cortex, AI: anterior insula, MCC: midcingulate cortex, PI: posterior insula.

To localize the empathy network in each participant, we used a modified version of a prior task^49^. Participants were shown 18 videos, each lasting for 3 s, of people experiencing painful auditory stimulation. After each video, participants provided ratings for the videos (see Fig. 6(a)). Mean inter-trial interval (ITI) was 2 s and was randomly jittered (jitter range: 0–2 s) to reduce predictability of the stimuli presentation.

### fMRI data acquisition and preprocessing

All fMRI scans were acquired using a 3T Philips Achieva scanner at the Hospital ‘Santa Maria della Misericordia’ (Udine, Italy), equipped with an 8-channel head coil, with standard parameters (see Supplementary Text S12). This study was conducted according to the principles in the Declaration of Helsinki, approved by the Ethics Committee of the Hospital ‘Santa Maria della Misericordia’ (Udine, Italy), and was carried out in accordance with the approved guidelines. All participants provided written informed consent before any study procedure was initiated.

Data were analyzed with SPM12. Each subject’s data were motion-corrected (outliers were detected using the Art toolbox) and then normalized onto a common stereotactic space. Data were smoothed by using a Gaussian filter (full width half maximum = 6 mm at first-level) and high-pass-filtered (see Supplementary Text S13 for a more detailed description).

### fMRI data analysis at first-level

For both moral judgment and empathy localizer tasks, the design matrices for fixed-effects General Linear Model were constructed by convolving a canonical hemodynamic response function (HRF) with the stimulus function for events (boxcar function) to create regressors of interest along with its temporal and dispersion derivatives. For a more detailed account, see Supplementary Text S13. Additionally, since we modelled each segment of the story and there was no jitter between these segments, there was possibility of high collinearity among regressors. Although we carried out collinearity diagnostic to address this issue (Supplementary Text S13), this still remains a limitation of the current study and future research should address this concern^18^.

#### ROIs selection and localization at individual level

At the first level, the following ROIs for empathy for pain were defined for each participant based on the localizer task (number of participants for whom an ROI was localized is given in parentheses): bilateral posterior insula (l-PI: 26/42, r-PI: 25/42), bilateral anterior insula (l-AI: 32/42, r-AI: 34/42), anterior cingulate cortex (ACC: 35/42), and midcingulate cortex (MCC: 38/42) (see Fig. 6(b)). The selection of ROIs was based on their consistency across quantitative and qualitative reviews^32, 35, 50–52^ (Supplementary Text S11). ROI coordinates were derived from the contrast: experimental video > baseline (higher BOLD activity while watching painful videos as compared to baseline; *p*(uncorrected) < 0.001, *k* > 10). Not all ROIs could be localized for all participants (see Supplementary Text S14).

#### ROI data extraction

The data from spherical ROIs with a radius of 8 mm was extracted and analyzed using the MarsBar toolbox (v0.44) for SPM (http://marsbar.sourceforge.net/). Within the ROI, the average percent signal change (PSC) was computed relative to the adjusted mean of the time series (for more details, see Supplementary Text S15). The responses of ROIs were measured while participants read the *mental-state information* (8 s) and *consequence* (8 s) segments of the moral stories and gave *acceptability* (6 s) and *blame* (6 s) judgments. PSCs were also extracted for the *background* and *foreshadow* segments, although no information was available at this stage for any morally relevant evaluation to commence. As recommended^53^, data defining ROIs was independent from the data used in the repeated measures statistics. This helps us sidestep the nonindependence error^54^ that can lead to spurious correlations and the observed results are thus unbiased and more trustworthy. Restricting analysis to a few ROIs also reduces Type-I error by drastically limiting the number of statistical tests performed^55^.

### fMRI data analysis at second-level

Since the primary analyses centered on ROI analyses, details of the analysis at the group level are provided in Supplementary Text S13.

### Exploratory functional connectivity analysis

Exploratory functional connectivity was carried out using standardized psychophysiological interaction (sPPI) analysis^56–58^. Specifically, we explored which brain regions showed changes in information exchange with the areas involved in decisions about blame (versus acceptability) for accidental harm cases. The ROI and whole-brain analysis revealed r-AI to be the only region that consistently tracked the outcome-by-judgment interaction (see Results) and thus this was chosen to be the seed region. We took the recommended precautions^59, 60^ while carrying out the PPI analysis (full details provided in Supplementary Text S16).

### Behavioral and ROI Data analysis

Statistical analysis was conducted with R programming language. Given that both the behavioral data (items within conditions within participants) and PSC data from ROIs in the empathy network (conditions within segments within ROIs within participants) had multilevel or nested structure, we utilized linear mixed-effects models (LMM) to correctly handle the inherent dependencies in nested designs and to reduce probability of Type I error due to reduced effective sample size^61–63^. Additional advantage provided by the LMMs over the traditional ANOVA analyses is that they do not depend upon various assumptions (e.g., symmetric variance-covariance matrix) and can deal with missing data^64^ (important for the current study as not all ROIs were localized for all participants). We note that there was a minimum of five observations per cell, a requirement for robust multilevel analysis^65^. Also, as recommended for confirmatory hypothesis testing^62^, all models included the maximal random effects structure (as long as no convergence issues were encountered). Maximum Likelihood (ML) estimation and variance components covariance structure were used.

### Data availability statement

Unthresholded statistical maps of reported contrasts are available on Neurovault^66^ at the following address: http://neurovault.org/collections/1712/. All the behavioral data are available at: https://osf.io/893eh/.

## Results

### Behavioral Results

**Figure 7:**
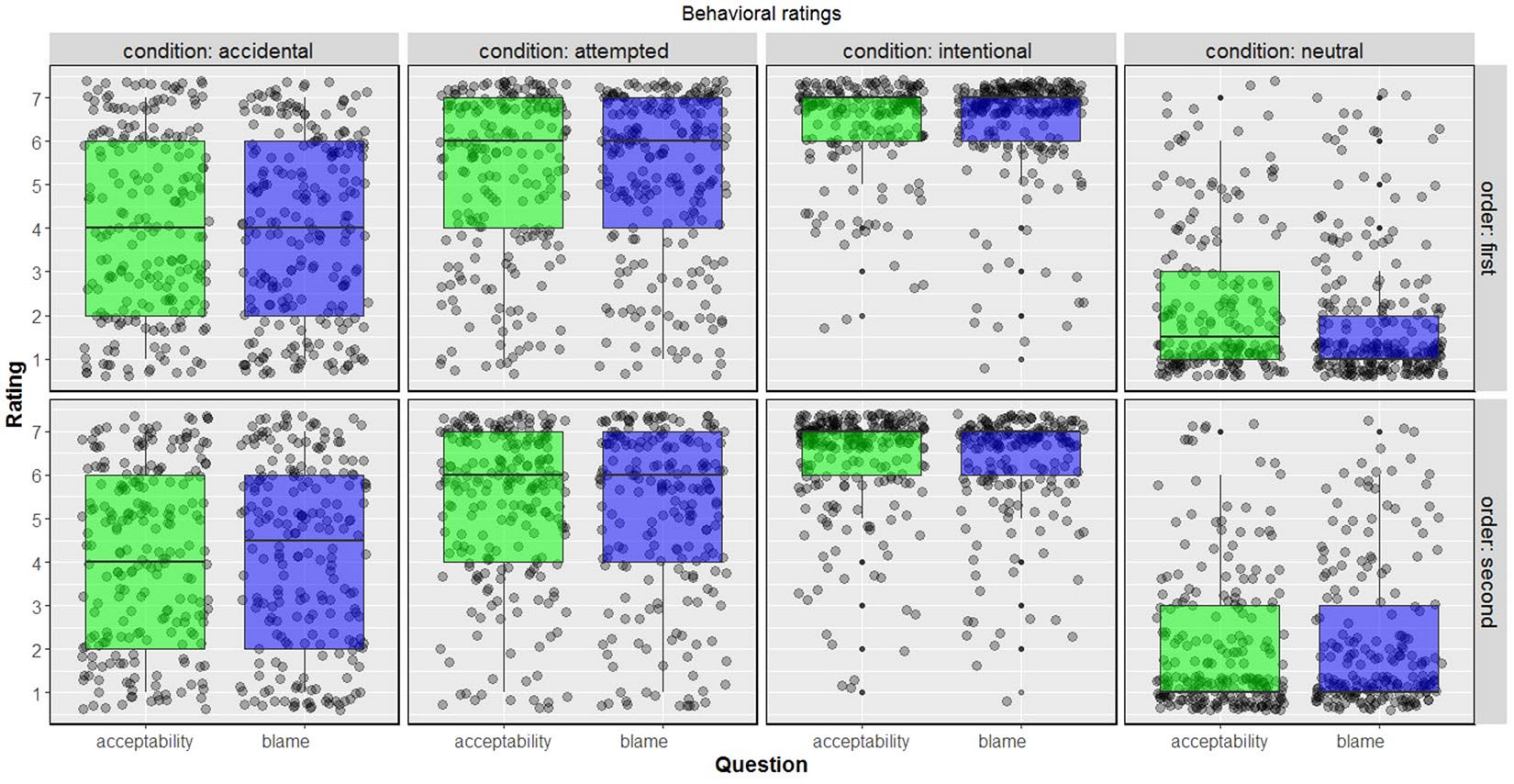
Behavioral results from the fMRI study. Boxplots with jittered data points for moral judgments given by participants in the fMRI study, displayed separately for each order. No outcome-by-judgment interaction was observed in either order. Note that higher ratings on acceptability question mean that the behavior was deemed to be *less* acceptable.

We used LMM that included fixed effects for all within-subjects factors: belief, outcome, type of question, and question order, as well as all possible two-way, three-way, and four-way interactions. We included random intercepts for participant. Given that previous work has revealed that presenting both types of moral judgments in within-subjects designs can lead to order effects^67^, we also included order in which questions were presented as one of the factors and present the results separately for each order (Fig. 7).

As expected from previous work, there was a main effect of belief and outcome and interaction between belief and outcome. But the fixed effect of interest to the current investigation, the interaction between outcome and the type of question/judgment, was not significant: estimate = 0.231, se = 0.233, *df* = 3370.585, *t* = 0.993, *p* = 0.321. Recall though that we did obtain this interaction in behavioral studies, reported as Studies 6–7, and this effect has also been observed in Italian sample^23^ with similar design (For possible explanation of this null effect, see Supplementary Text S20). All other fixed effects and descriptive statistics are provided in Supplementary Text S17.

## fMRI data analysis results

### Greater empathy network response while encoding outcome information

If the empathy network tracks the harm that a victim suffers during stimulus presentation, then the entire network should be selectively more active for part of the story when outcomes to the victim are described (*consequence* segment) than for other parts of the story dominated by other types of information (e.g., background of the story (*background* segment), foreshadowing of the outcome (*foreshadow* segment), or information about belief states provided during the (*mental-state information* segment). In other words, the response in the empathy network at encoding stage should be stimulus-bound^6^: modulated by the presence or absence of information about the victim’s resulting state.

**Figure 8:**
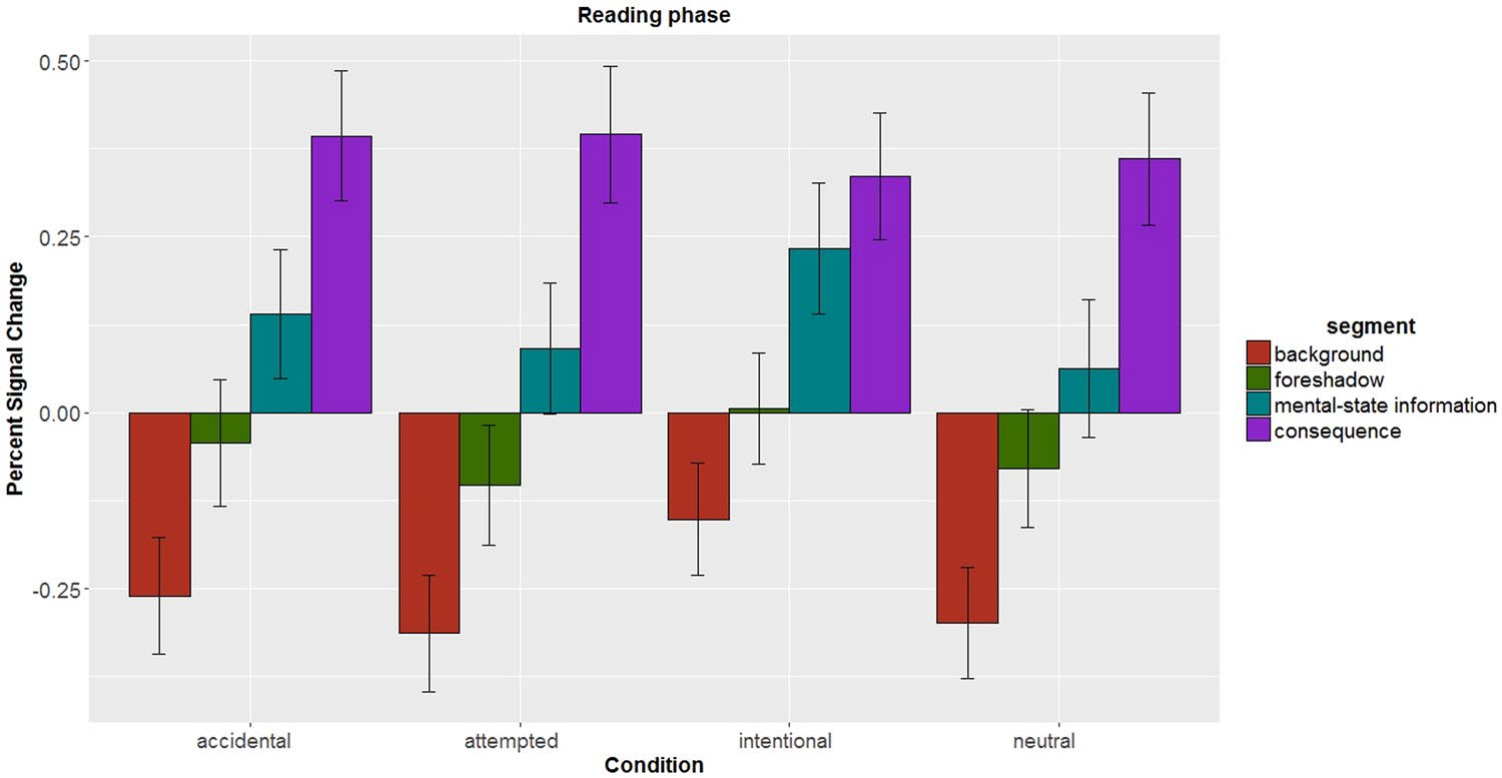
The empathy network encodes harmfulness of outcomes. The estimated PSC across all ROIs of the empathy network during the reading phase text, i.e. when the participants were provided with information about background, foreshadow, beliefs (*mental-state information*), and outcomes (*consequence*). The PSC in the network was significantly greater than the baseline when outcome information was provided, but not when other type of information was provided. Error bars correspond to 95% confidence intervals.

Indeed, LMM on the PSC data from reading phase segments revealed a main effect of *consequence* segment, such that the empathy network showed greater PSC during *consequence* segment than background (estimate = 0.655, se = 0.0553, *z* = 11.855, *p* < 0.001), foreshadow (estimate = 0.436, se = 0.0552, *z* = 7.910, *p* < 0.001), and mental-state information (estimate = 0.297, se = 0.0551, *z* = 5.390, *p* < 0.001) segment (see Fig. 8). All other fixed effects and graphical illustrations are provided in Supplementary Text S18. Thus, the empathy network was more active when information about harmfulness of outcomes was provided than when other type of information was presented. These results reveal neural basis of the empathic process by which harmfulness of outcomes is encoded.

### Empathy network response predictive of moral judgments for negative outcome conditions

Next, we assessed the relationship between the activation of the empathy network and moral judgment. We predicted that the degree to which the entire empathy network responds to outcome information, both during reading phase (*consequence* segment, when this information is provided for the first time) and during the judgment phase (the *acceptability* and *blame* segments combined, when this information is recruited in the service of moral condemnation), will be predictive of the severity of moral condemnation, irrespective of the type of judgment. Additionally, this pattern of response should hold only for negative outcome conditions where a victim is harmed (accidental and intentional harms, i.e.) and not for neutral outcome conditions where no salient information about harm is present (neutral and attempted harms, i.e.).

**Figure 9:**
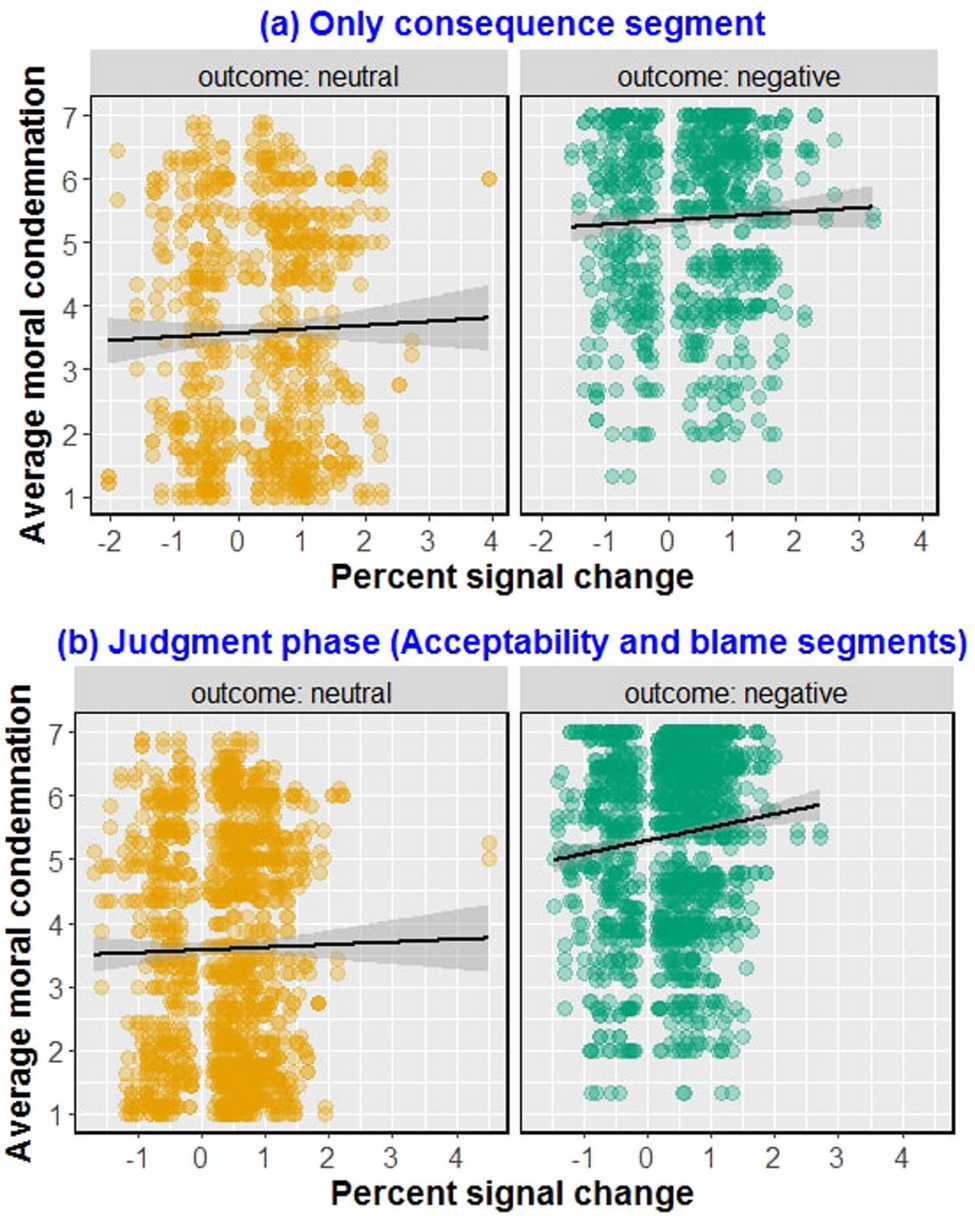
Brain-behavior correlation. The degree to which the empathy network was active (Percent Signal Change) while (**a**) reading information about harmfulness of outcomes and (**b**) providing ratings on judgments predicted the severity of moral judgments for actors who caused harmful outcomes.

As hypothesized, the mixed-effects regression on PSC in the empathy network during the judgment phase showed that moral condemnation was predicted by activity in the empathy network, more so far harmful outcomes than neutral outcomes (PSC × outcome: estimate = 0.1959, se = 0.0628, *df* = 2947.94, *t* = 3.1188, *p* = 0.0018). A similar effect was also found for the *consequence* segment in the reading phase (PSC × outcome: estimate = 0.1477, se = 0.0710, *df* = 457.99, *t* = 2.0798, *p* = 0.0377). In other words, both the initial encoding of the affective state of the victim during consequence segment, and the integration of this information while making judgments determined the degree to which empathic reaction towards the victim informs judgments (see Fig. 9). This result also underscores that the empathic response in this network was directed at the victim and not the perpetrator, since- (*i*) higher empathic response with the perpetrator would have predicted *reduced* moral condemnation; (*ii*) no information about affective state of the perpetrator was provided in the stimuli (for more detailed discussion, see Supplementary Text S19).

### Differential empathy network response to accidental harms for different judgments

If it is true that the empathy network is encoding morally relevant harmful outcomes (as shown by preceding results) and harmful outcomes matter more for blaming as compared to assessing acceptability of third-party moral violations, the empathy network should be more active for blame judgments as compared to acceptability judgments. This should specifically be true for moral luck (accidental versus neutral) contrast, since at the behavioral level the severity of endorsed blame for an agent who accidently produced harm while acting under false belief is increased significantly more than acceptability with reference to a neutral case^1, 23^. Thus, we expected the empathy network to be active to a greater degree while people were assigning blame to accidents as compared to assessing the acceptability of an accident, but for no other condition.

**Figure 10:**
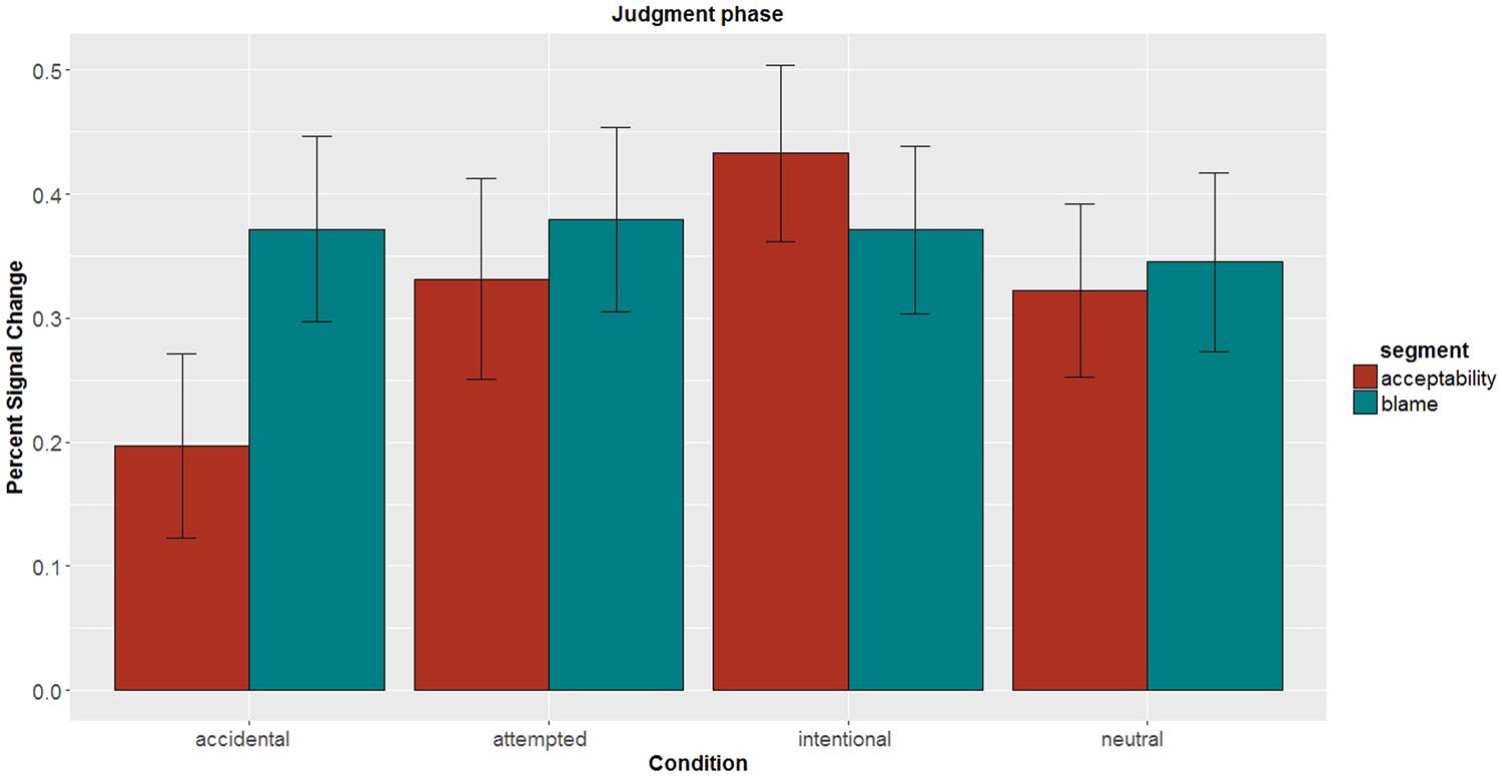
The empathy network activity during the judgment phase. The estimated PSC across all ROIs of the empathy network during the judgment phase, i.e. when the participants made two types of moral judgments. The PSC was higher for blame as compared to acceptability judgments only for accidental harm scenarios (*p* < 0.001). Error bars correspond to 95% confidence intervals. *Abbreviations*–PSC: percent signal change, ROI: region of interest.

The LMM carried out on PSC data from the judgment phase segments (*acceptability* and *blame*, i.e.) revealed that this was indeed the case. There was a significant fixed effect for the interaction belief × outcome × segment: estimate = −0.2622, se = 0.0841, *df* = 2940, *t* = −3.119, *p* = 0.002). All other fixed effects are provided in Supplementary Text S20. This interaction reflects that the empathy network was active to a greater degree for *blame* as compared to *acceptability* segments only for the case of accidental harm (neutral belief and negative outcome: estimate = 0.175, se = 0.042, *t* = 4.149, *p* < 0.001), but not otherwise (see Fig. 10).

To see which subregions of the empathy network showed the moral luck effect, we carried out exploratory *post hoc* analyses, which revealed that the pattern of greater PSC for blame versus acceptability judgments for accidents was seen in r-AI, r-PI, and ACC (*p* < 0.05). But, at the group level in whole-brain analysis (*p*(FWE) < 0.05), the moral luck effect was observed only in r-AI (full details in Supplementary Text S21).

Prior behavioral research on the judgment of accidental harm cannot distinguish two alternative regulation hypotheses: information about harmful outcomes may be either (*i*) selectively *down*-regulated while assessing acceptability of accidents, or instead (*ii*) selectively *up*-regulated while making blame judgments. We can address this question by interrogating the overall activation profile of the empathy network across our design. Comparing accidental harm condition with other conditions favors the former account because the activity for accidental harm condition is attenuated (as compared to other conditions) only for acceptability judgments but not for blame judgments. In other words, it appears that acceptability judgments are insulated from the influence of information about accidental harm due in part to the selective a down-regulation of the empathy network. To further probe this effect, we carried out functional connectivity analysis on r-AI, since this was the only ROI which consistently showed the moral luck effect across both ROI and whole-brain analyses.

### Functional connectivity results

To investigate the neural regions that exhibited changes in functional connectivity with r-AI while making acceptability and blame judgments for accidental harm condition, exploratory psychophysiological interaction (PPI) analyses were conducted during judgment phase segments. This analysis revealed that r-AI exhibited decreased exchange of information (negative PPI effect, i.e.) with the left middle frontal gyrus or l-dlPFC (left dorsolateral prefrontal cortex; *x* = −34, *y* = 10, *z* = 36; *β* = −0.2480, *t* = −4.0582, *k* = 23, *p*(uncorrected) = 0.0003) while making acceptability as compared to blame judgments. Notably, the dlPFC is associated with two functions of relevance to moral decision-making, information integration^16, 45^ and causal reasoning^68^. We return to consider the significance of this finding within our preferred account more fully below.

## Discussion

Our findings indicate that empathy for victims contributes to the moral condemnation of harmful acts (see Supplementary Text S22 for extended discussion). This role is particularly strong in cases of accidents, when a person causes harm that they did not intend, and thus provides an explanation for the phenomenon of “moral luck”. Several convergent behavioral and neural effects support this model of empathic blame. First, the condemnation of accidents is associated with increased perceptions of a suffering victim, and also with increased trait empathy. Second, among a network of brain regions that reliably activate during empathy for pain, we find increased activation as people learn about the harm caused by third-party action, and again when they make moral judgments of that action. Third, individuals who show the greatest neural response in the empathy network to harmful actions also show the greatest moral condemnation of the perpetrator. Finally, consistent with prior research showing that blame judgments are especially susceptible to moral luck effects, we show greater activation of the empathy network when people judge blame for an accidental harm, compared with judgments of acceptability. This effect is unique to the case of accidental harm. Together, these results indicate that people assign blame even to unintentional harmdoers in part because of their empathy for the suffering of a victim, and illuminate the neural basis of this effect.

Surprisingly, we observed identical overall activation levels in the empathy network during the presentation of information about harmful and neutral outcomes. Apparently, even in conditions where no harm was presented or implied in the stimulus, participants simulated the possibility of harm, or mirrored its absence, nonetheless^69^. We regard it as unlikely that people continuously evaluate the possibility of harm for all neutral events; rather, the presence of conditions with harmful outcomes in our experiment likely produced a relative contrast effect^69^. This is in line with prior studies which show that neural responses to events can be modulated by the overall contextual setting in which these events take place^70^ and behavioral reactions towards a particular stimulus is contingent on affective properties of other stimuli concomitant with it^71, 72^. This finding indicates that in some contexts activation of the empathy network can be proactive (i.e., driven by the participants’ expectations) rather than strictly reactive (i.e., driven by properties of the stimulus itself). A similar effect was also observed in the self-report data (Study 5), such that participants reported to have felt empathy for victims in situations where there was no harmful outcome (Fig. 2). By analogy, past work focusing on mentalizing during moral judgments which also shows that the mentalizing network tends to exhibit significant activation both in presence and absence of harmful intent^2, 6^.

Similarly, we observed re-activation of the empathy network when participants made moral judgments of all four event types—a period during which no additional harm-relevant information was provided. Again, this finding has a natural analog in prior research on the neural basis of intent-based moral judgment: The mentalizing network reactivates during the judgment of cases both including and excluding a culpable mental state^2, 6^. We propose that the re-activation of the empathy network reflects the retrieval of previously encoded representations of the harmfulness of outcomes, facilitating integration with other morally-relevant content^73^.

An analysis of functional connectivity revealed that r-AI, which exhibits the clearest neural instantiation of the moral luck effect, showed context-sensitive connectivity pattern with dlPFC. When judging accidents, dlPFC showed tighter coupling with putative harm representations in r-AI for blame judgments than for acceptability judgments, mirroring the greater behavioral influence of harm for blame than for acceptability. This finding sits comfortably with the emerging consensus regarding the role of dlPFC as a superordinate, integrative node in decision making system that combines representations of inputs from multiple subprocesses to reach a final output that biases response selection^8, 16, 18, 45^, the so-called “integration-and-selection” function of dlPFC. In case of third party moral judgment, information about intent and outcome must be integrated into an overall judgment. Because acceptability judgments are dominated by the assessment of intent, while blame judgments rely additionally on the empathic representation of the harm to the victim^1^, the latter judgment type may invoke greater coupling with a brain region implicated in integration. This account makes a prediction, currently untested, that key nodes of the mentalizing network would also show coupling with dlPFC reflecting their role in representing the mental state of the harmdoer. An alternative and viable interpretation stems from prior research implicating the dlPFC in assigning causal responsibility to agents^68^. Thus, coupling between l-dlPFC and r-AI may reflect the integration of information about harm to a victim and the perpetrator causally responsible for that harm. Resolving these accounts, which are currently speculative, remains an important area for future research.

Although we refer to an “empathy network”, this is not intended to imply a network *specific* to empathy, but rather one that responds *reliably* to empathy for suffering^32, 35, 47, 52, 74, 75^. Indeed, past research shows that these regions are involved in the processing of a variety of non-nociceptive, multimodal sensory inputs^52, 76^ and undergird a host of other cognitive and affective functions^48, 77–81^ and can be found to be active even in the absence of subjective sensation of pain^82^. Nevertheless, the tight correspondence between activation of this network, stimuli concerning victim pain (e.g., a rabid dog biting an older lady, skiers breaking their legs in accident, etc.), and participants’ moral judgments favor the conclusion that this network indexes empathy for suffering in our task.

In summary, we provide evidence that the empathy network supports the encoding and integration of harm representations during moral judgment. These results indicate a novel role for a well-studied functional network of brain regions, while also refining current theories of moral judgment. Finally, they clarify the psychological basis of moral luck–a longstanding matter of philosophical and legal concern^17^.

## Electronic supplementary material

Supplementary information
