# Supplementary material for: The behavioral and neural basis of empathic blame
Source: Sci Rep. 2017 Jul 12;7:5200. doi: 10.1038/s41598-017-05299-9 (PMC5508012; doi:10.1038/s41598-017-05299-9)
Supplement: Supplementary file 1 — Supplementary information [file 41598_2017_5299_MOESM1_ESM.pdf]

Supplementary information for "The behavioral and neural basis of empathic blame"

Indrajeet Patil[1,3], Marta Calò[2], Federico Fornasier[2], Fiery Cushman*[3], Giorgia Silani*[4]

[1]Scuola Internazionale Superiore di Studi Avanzati, Neuroscience Sector, Trieste, Italy.
[2]University of Trieste, Trieste, Italy.
[3]Department of Psychology, Harvard University, Cambridge, USA.
[4]Department of Applied Psychology: Health, Development, Enhancement and Intervention, University of Vienna, Austria.

* These authors made equal contributions to this work and share senior authorship.

Correspondence should be addressed to:

Indrajeet Patil, 33 Kirkland Street, Cambridge, Massachusetts, 02138, USA.

E-mail: patilindrajeet.science@gmail.com

Abbreviations: sd = standard deviation, se = standard error, ci = 95% confidence interval, N = sample size

N.B.: All estimates from regression models are unstandardized.

Supplementary Text S1: Acceptability versus blame

Moral judgment is an umbrella term and, consequently, not all types of moral judgments are equivalent. They can broadly be grouped in distinct classes based on their evaluative foci (Cushman, 2015; Malle, Guglielmo, & Monroe, 2014). The wrongness/acceptability/permissibility (henceforth, represented only by the term *acceptability*) represent a class of judgments that are concerned with evaluation of *actions* (or *action plans*) with respect to a norm system and functions to *declare* that the behavior is incongruent with mutually agreed upon moral norms (e.g., "Do not harm others"). On the other hand, blame and punishment together represent a class of judgments that focuses on evaluating *agents* for their involvement in norm violating events and functions as a social mechanism to *regulate* their behavior to deter repetition of such behavior in future.

Although adult human moral judgment is primarily modulated by information about intent, outcomes play a substantial role too (Cushman, 2008) and plenty of psychological research provides evidence for this "outcome bias" in lay judgments (Berg-Cross, 1975; Cushman, 2008; Cushman, Dreber, Wang, & Costa, 2009; Mazzocco, Alicke, & Davis, 2004; Patil, Young, Sinay, & Gleichgerrcht, 2017). More importantly, outcomes matter to a different degree for different classes of moral judgments: acceptability judgments exhibit lesser sensitivity to outcome information as compared to blame/punishment judgments (Cushman, 2008). In other words, evaluation of acceptability of agent's moral behavior primarily relies on assessment of actor's mental state during the act and on determining culpability of this state with respect to normatively acceptable code of conduct, while the blame/punishment for the agent additionally involves appraisal of whether harm occurred, severity of harm caused, and actor's causal involvement in production of harm (Cushman, 2015).

Supplementary Text 2: Empathic concern subscale and moral judgments (Study 1)

Objective of the current study was to replicate prior finding that empathic concern, which is often mistakenly taken to be an index of empathy, is associated with harsher moral condemnation. As we

highlight in the main text and throughout supplementary, we are using the term *empathy* in the sense of affective sharing (“I feel what you feel”), while *empathic concern* represents a general concern for others’ wellbeing (“I am concerned about how you feel”) (Batson, 2009; de Vignemont & Singer, 2006; Decety & Cowell, 2014a; Jordan, Amir, & Bloom, 2016).

Participants

Two hundred and eight participants (98 females) were recruited through Amazon Mechanical Turk and all provided informed consent. Level of education varied widely (from high school to PhD) and so did political orientation and religiosity. All participants received monetary compensation for their participation and all procedures were approved by the Brown University Institutional Review Board.

Methods and Materials

As opposed to the main study, here we focused only on the accidental and attempted harm scenarios. Each participant read 8 stories (4 accidental harm, 4 attempted harm) taken from prior a study (Young, Camprodon, Hauser, Pascual-Leone, & Saxe, 2010). Stories were counterbalanced across conditions and across participants. After reading each story, participant rated moral permissibility of the behavior on a 7-point Likert scale (1: *Morally forbidden*, 7: *Morally permissible*). Scores were averaged to create a mean permissibility scores for accidental (Cronbach’s $\alpha = 0.688$) and attempted ($\alpha = 0.713$) harm scenarios.

After completing the moral judgment task, participants completed the Interpersonal Reactivity Inventory (IRI) (Davis, 1983). This is a 28-item self-report questionnaire, with four 7-item subscales, used to assess specific aspects of dispositional empathy by asking participants to report agreement with statements on a 5-point Likert scale (1: *never true for me*, 5: *always true for me*). We focused on two subscales in particular, empathic concern (EC) ($\alpha = 0.906$) and perspective-taking (PT) ($\alpha = 0.843$), because they were of *a priori* interest to us based on past work (Patil & Silani, 2014; Trémolière & Djeriouat, 2016).

Results (wide format data)

As expected attempted harms (M = 2.243, SD = 1.184) were judged to be less permissible than accidental harms (M = 4.541, SD = 1.513) ($t(207) = 15.983$, $p < 0.001$, $d = 2.22$).

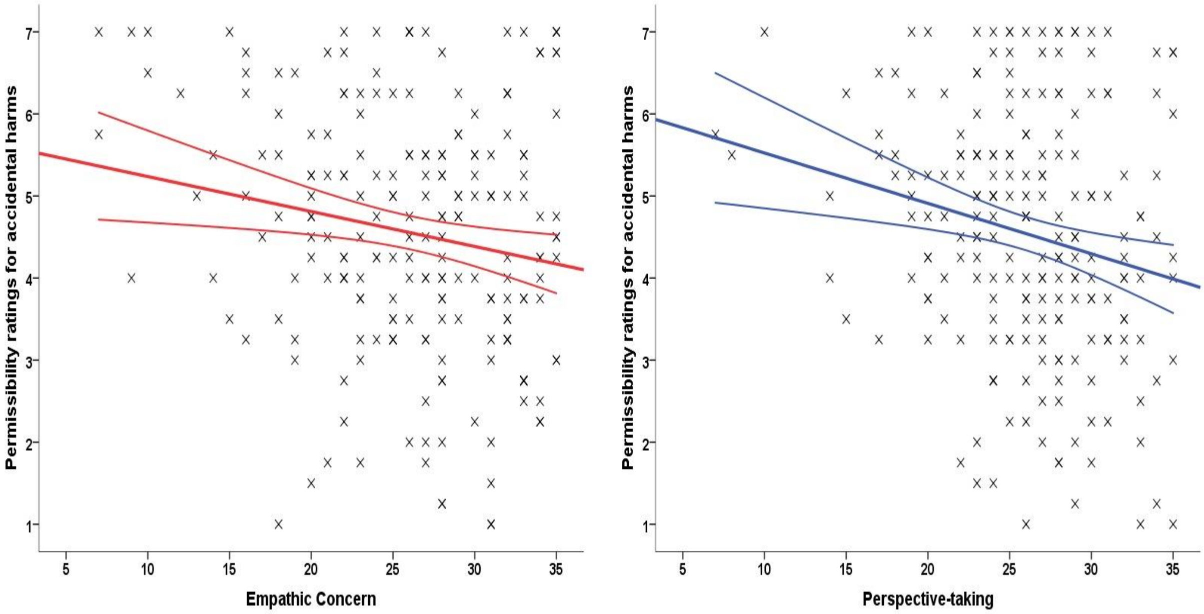

As expected, participants scoring high on dispositional EC found accidental harms to be less permissible ($\rho = -0.144$, $p = 0.039$) and, similarly, higher scores of PT ($\rho = -0.198$, $p = 0.004$) were predictive of harsher condemnation of accidental harms. Additionally, increased EC was also associated with reduced permissibility for attempted harm cases ($\rho = -0.187$, $p = 0.007$), but not PT ($\rho = -0.111$, $p = 0.111$). Although surprising, this result has also been observed in prior studies using IRI measure (Patil & Silani, 2014; Trémolière & Djeriouat, 2016).

Results (long format data)

Ordinal mixed-effects regression

Given the nested structure of the data, we also carried out analysis on long-format data to account for any item or participant level effects. Since our data were discrete (1–7 Likert scale) rather than continuous, we

carried out ordinal mixed effects regression (specifically, we fit a cumulative link mixed model) as an additional robustness check (R package: `ordinal`). The syntax used was the following-

```
clmm(rating ~ EC + (EC | item) + (1 | id), data = subset(dataset,
condition == "condition of interest"))
```

No random effect for question was included given that it has less than 3 levels.

condition	estimate	se	z	*p*-value
accidental	-0.364	0.149	-2.439	0.015
attempted	-0.353	0.178	-1.982	0.048

As found with the mean scores for accidental and attempted harm conditions, the mixed-effects regression also revealed that participants scoring high on EC were harsher (*less* morally permissible ratings, i.e.) in their assessment of both accidental and attempted harms.

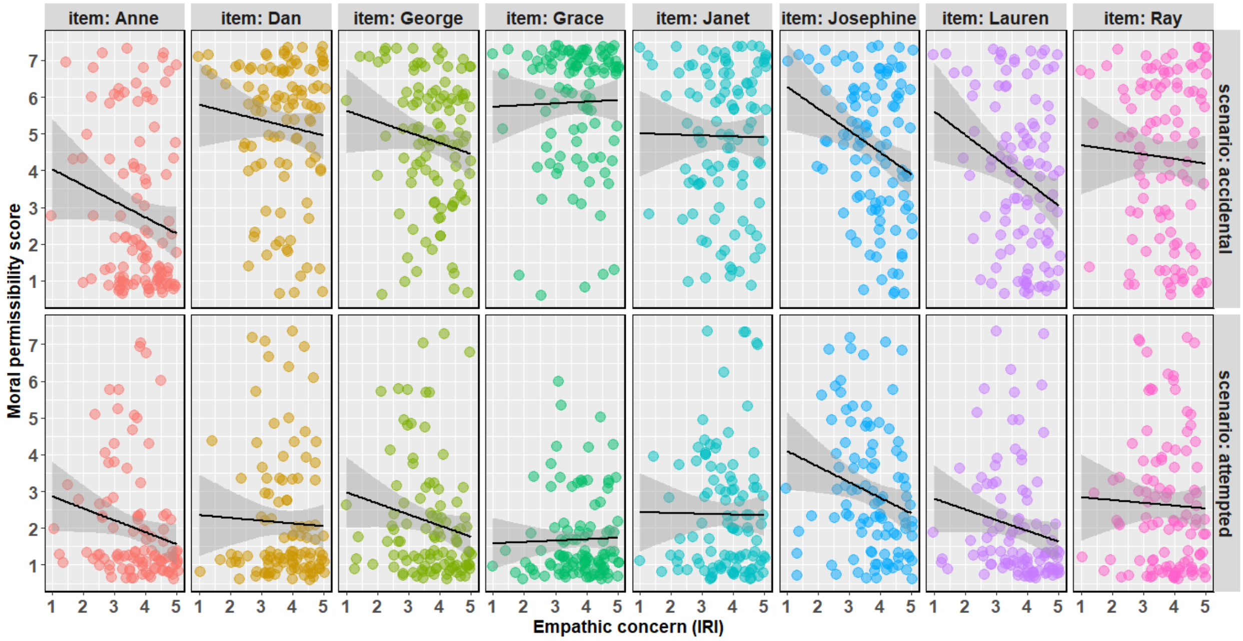

Limitations

Note that, at face value, the positive association between EC and condemnation for attempted harm cases seems to contradict our empathic blame hypothesis. Since there was no harmful outcome in attempted

harm cases, there should not be an empathic response towards victim, and so the hypothesis predicts, no association between empathy and moral judgment.

We reiterate here that we are defining empathy as sharing of others' emotional states by *experiencing* these emotional states while maintaining self-other distinction (de Vignemont & Singer, 2006; Decety & Cowell, 2014b). Defined this way, empathic concern subscale of IRI is a poor measure of the underlying construct. Indeed, recent work suggest that this subscale measures sympathy or compassion and not empathy (Jordan et al., 2016). We nonetheless used EC-IRI for this behavioral study not because it is an ideal measure, but because it has received the most attention in prior work. Thus, the association between EC and attempted harm should not be interpreted as a refutation of the empathic blame hypothesis.

Supplementary Text S3: Empathy index and severity of moral judgments (Study 2)

In this study, we utilize the newly developed empathy index (Jordan et al., 2016) to measure empathy (in the sense of "I feel what you feel") and see if we could replicate the results obtained with EC subscale of IRI. Empathic blame hypothesis would predict a significant positive association between higher scores on empathy index and moral judgments of actions that resulted in harmful outcomes, but not for neutral outcomes.

Participants

Five hundred and two participants (276 males, 224 females, 2 other) were recruited through Amazon Mechanical Turk and all provided informed consent. Level of education varied widely (from high school to PhD) and so did age, political orientation, and religiosity. All participants received monetary compensation ($0.25) for their participation and all procedures were approved by the Harvard University Institutional Review Board.

Methods and Materials

After reading these instructions, participants were shown moral vignettes which consisted of four variations of 4 unique scenarios for a total of 16 stories. The four variations were result of a 2 × 2 within-subjects design. Each participant saw only one variation of each scenario, for a total of 4 stories. The order of presentation of scenarios was randomized across subjects.

After reading each scenario, participants provided ratings of four questions (presented on the same screen) on a 7-point Likert scale:

> (*i*) *wrongness*: How wrong was [the agent]'s behavior? (1: *Not at all*, 4: *Somewhat*, 7: *Very much*);

> (*ii*) *punishment*: How much should [the agent]'s be punished? (1: *None at all*, 4: *Some*, 7: *A lot*)

After providing moral judgment ratings for the scenarios, participants rated the following sentences from the empathy index (Jordan et al., 2016) on a 5-point Likert scale (1: *Does not describe me well*, 5: *Describes me well*):

- If I see someone who is excited, I will feel excited myself.
- I sometimes find myself feeling the emotions of the people around me, even if I don’t try to feel what they’re feeling.
- If I’m watching a movie and a character injures their leg, I will feel pain in my leg.
- If I hear a story in which someone is scared, I will imagine how scared I would be in that situation and begin to feel scared myself.
- If I hear an awkward story about someone else, I might feel a little embarrassed.
- I can’t watch shows in which an animal is being hunted one another because I feel nervous as if I am being hunted.
- If I see someone fidgeting, I’ll start feeling anxious too.

Results

The descriptive statistics are tabulated below:

question	condition	N	rating	sd	se	ci
punishment	accidental	502	2.546	1.798	0.080	0.158
	attempted	502	3.161	2.022	0.090	0.177
	intentional	502	5.141	1.836	0.082	0.161
	neutral	502	1.480	1.131	0.050	0.099
wrongness	accidental	502	3.006	1.999	0.089	0.175
	attempted	502	4.434	2.149	0.096	0.188
	intentional	502	5.916	1.462	0.065	0.128
	neutral	502	1.673	1.389	0.062	0.122

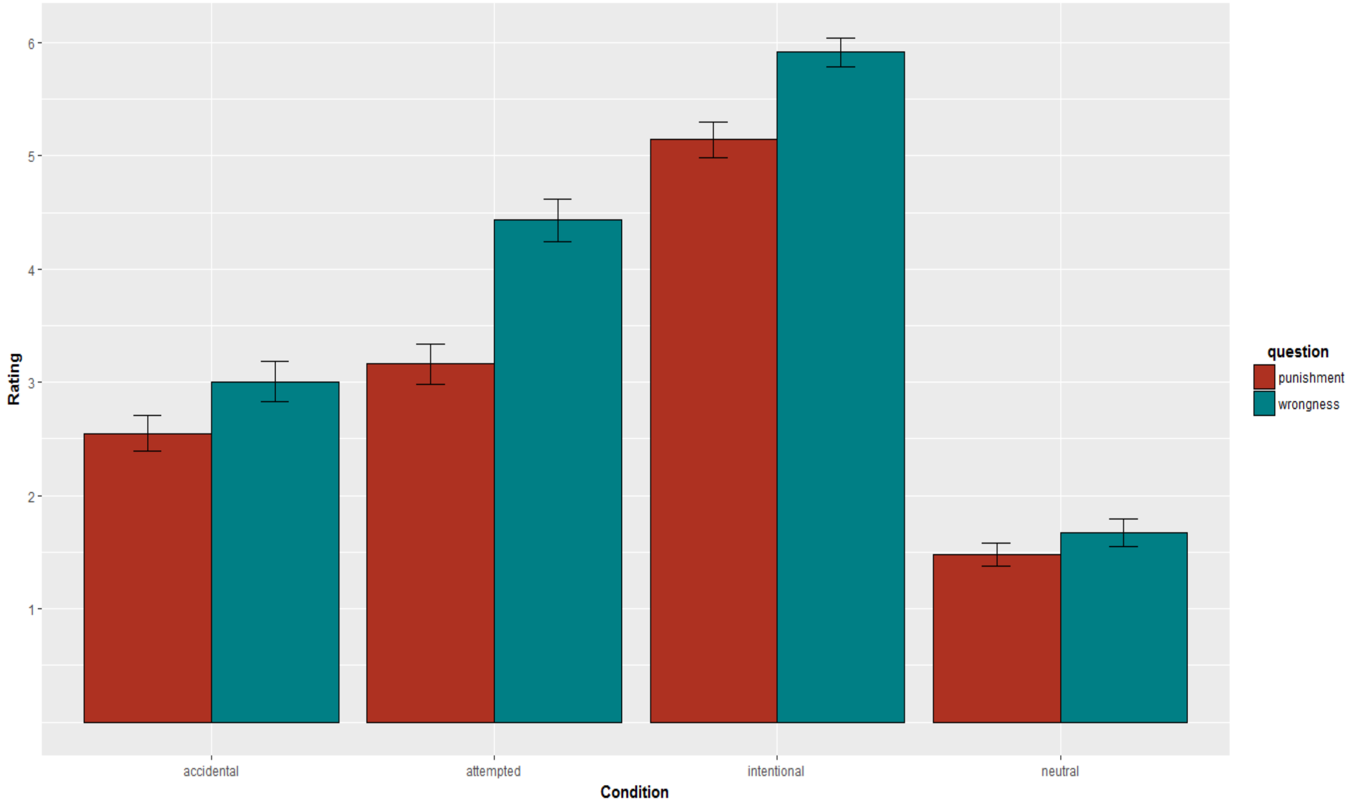

Empathy index items showed good internal reliability (α = 0.840) and were thus averaged to give a unique empathy index for each participant.

Linear mixed-effects regression

We carried out linear mixed-effects regression (random slopes for item and type of question and random intercept for participant) separately for each condition to see if there was a positive association between Empathy index and severity of judgment. R syntax for each condition was the following:

```
lmer(rating ~ Empathy_index + (1 + Empathy_index | item) + (1 +
Empathy_index | question) + (1 | id), data = subset(data_file),
condition = "condition of interest"))
```

The results revealed that empathy index was predictive of severity of moral judgments of actions, irrespective of whether the actions produced neutral or harmful outcomes.

**condition**	**estimate**	**95% percentile ci**		**se**	***df***	***t* value**	***p*-value**
		**LB**	**UB**				
neutral	0.260	0.053	0.460	0.098	4.850	2.662	0.046
accidental	0.348	0.171	0.515	0.090	23.591	3.857	0.001
attempted	0.343	0.158	0.525	0.092	18.083	3.718	0.002
intentional	0.179	-0.006	0.350	0.094	5.966	1.916	0.104

Note: The percentile bootstrap confidence intervals for estimates were computed from 1000 permutations.

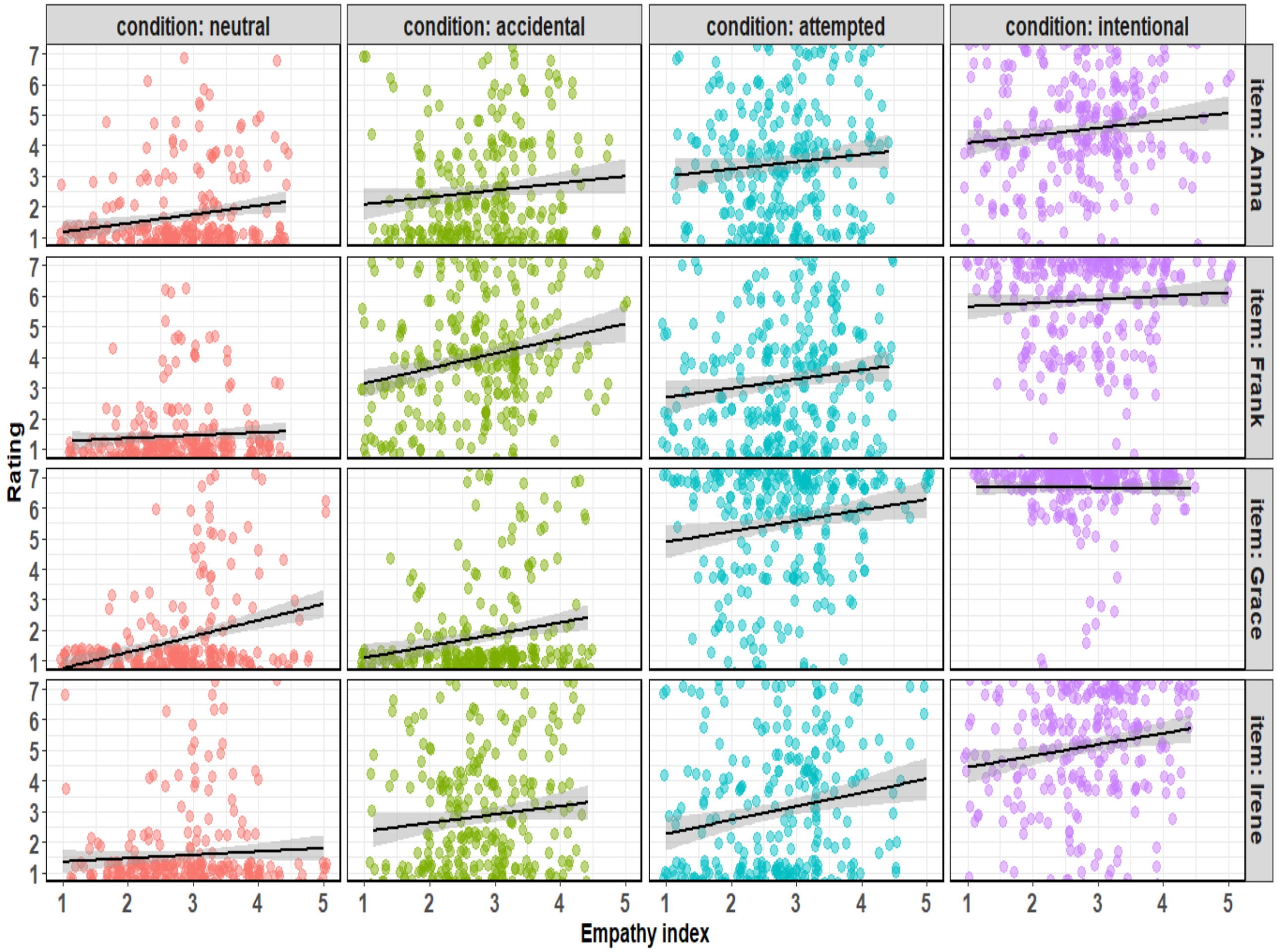

Ordinal mixed-effects regression

Since our data were discrete (1–7 Likert scale) rather than continuous, we additionally carried out ordinal mixed effects regression (specifically, we fit a cumulative link mixed model) as an additional robustness check (R package: `ordinal`). The syntax used was the following-

```
clmm(rating ~ Empathy_index + (1 + Empathy_index | item) + (1 | id), data = subset(dataset, condition == "condition of interest"))
```

No random effect for question was included given that it has less than 3 levels.

condition	estimate	se	z	p -value
neutral	0.456	0.162	2.824	0.005
accidental	0.882	0.222	3.975	< 0.001
attempted	0.461	0.116	3.981	< 0.001
intentional	0.264	0.183	1.444	0.149

Limitations

Although the positive association between Empathy index and neutral and attempted harms seems to be incongruent with the empathic blame hypothesis, another possibility is that Empathy index, although touted to be a more unadulterated measure of affective sharing (Jordan et al., 2016), is also tapping into a more general empathic concern for others and empathic concern has indeed been associated with more severe judgment for all conditions (Patil & Silani, 2014).

To address this possibility, we carried out one pilot study and one additional replication study on Amazon Mechanical Turk to see if there was any association between IRI-EC and Empathy index. The results from both pilot (n = 68, r = 0.66, p < 0.001) and replication (n = 78, r = 0.52, p < 0.001) studies revealed that indeed there was a large correlation between Empathy index and IRI-EC.

Since both *trait* measures we used were closely associated and tracked a general concern for the wellbeing of others, we instead decided to use *state* measures of empathy.

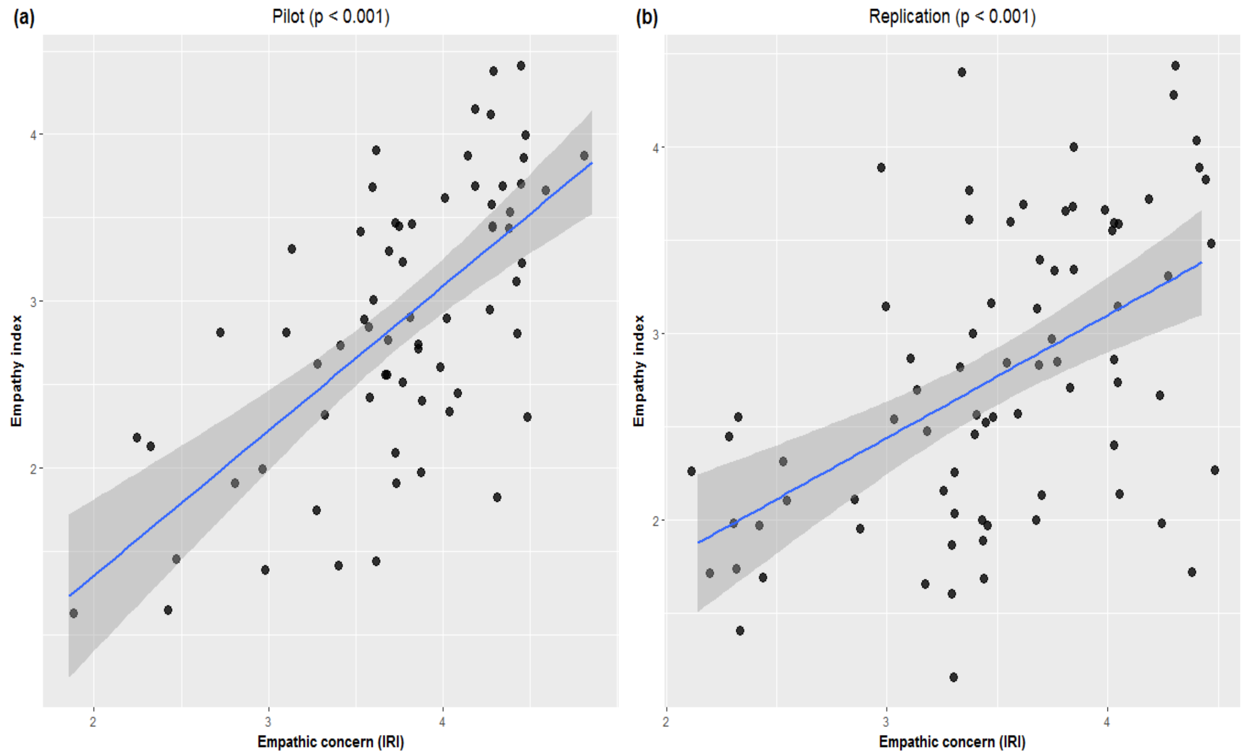

Supplementary Text S4: Empathy Compassion Task (ECT) and moral judgments (Study 3)

In this study, we utilize the newly developed ECT task (Miller & Cushman, 2017) to measure empathy ("I feel what you feel") and sympathy ("I am concerned about how you feel"). Empathic blame hypothesis would predict a significant positive association between higher scores on empathy (ECT) and moral judgments of actions that resulted in harmful outcomes, but not for neutral outcomes.

Participants

Hundred and ten participants (58 females) were recruited through Amazon Mechanical Turk and all provided informed consent. Level of education varied widely (from high school to PhD) and so did age, political orientation, and religiosity. All participants received monetary compensation ($0.25) for their participation and all procedures were approved by the Harvard University Institutional Review Board.

Methods and Materials

ECT task

The ECT task contains six brief vignettes (ranging from 110-175 words) that are presented in succession. Each vignette focuses on a protagonist having a negative affective reaction to an unpleasant situation. Full details of the task and more detailed description of all used vignettes are provided in the associated publication (Miller & Cushman, 2017).

Given that there is a discrepancy between the way lay people and researchers use the terms "empathy" and "compassion," participants are first reminded of the distinction between these two terms using the following text:

> "In this study, you will read some stories and answer few questions related to these stories. We will be interested in your assessment of the feelings of the characters involved in the story. If the answers to these questions seem obvious, that's ok. Just go ahead and give the obvious answer. If they seem difficult, that is also ok. There are no trick questions and we are just interested in your honest opinion. Before you begin, please read the following text carefully, since it emphasizes differences between two types of other-oriented emotional responses we are interested in for the current study.
>
> When people hear about or see others in a stressful or painful situation, they often respond in one of two ways.
>
> *Empathy*:
>
> On the one hand, they might feel like they're experiencing the same thing as the other person. In other words, they may mirror their feelings. If they hear that another person is afraid, they may feel fearful themselves. Or, if they hear about another person in pain, they may wince, as if they too are in pain. You can think of this as feeling with the other person.
>
> *Compassion*:

> On the other hand, they may feel compassion for the other individual. This feeling is often described as a positive feeling of warmth, tenderness, or concern, and it is usually accompanied by a strong desire to help. To some degree, compassion involves understanding what the other person is going through, but it doesn't require mirroring their feelings. To distinguish this emotion from the former one, you can think of it as feeling for the other person, rather than with. Of course, it is possible to feel both of these things at the same time, or neither at all. There is no "right" response, and the response each person has may depend on the particular circumstance."

After this introduction, they read vignettes and provide ratings of empathy and compassion for characters undergoing negative experiences. We provide one representative example of a vignette that features a woman who accidentally falls into a large glass window and is left with severe cuts on her hands and face:

> *It's a brisk, snowy December evening, and Caroline decides to go for a stroll through her town's shopping district to look at the Christmas lights and storefront decorations. She puts on her snow boots, wraps her scarf snugly around her neck, and walks three blocks to where the local cafés and boutiques begin.*
>
> *Caroline is enjoying her solitude and the quiet of the streets, when suddenly she comes across a patch of black ice on the sidewalk. Her right boot slides across the slippery surface, throwing her off balance. She tries to stabilize herself, but her centre of gravity is too far off and she can't gain any traction. Her body veers uncontrollably to the right, and she smashes into the window of the adjacent storefront. The glass is thin, and the force causes the window to shatter all around her. The jagged shards of glass dig deep into the skin of her exposed face and hands, leaving Caroline with several painful cuts.*

Below the vignette, participants were provided with a range of negative affective responses across scenarios (3 primarily involve pain, 1 anxiety, 1 embarrassment, and 1 fear) to get a more robust measure

of how participants respond after reading about others' negative experiences. At the end of each vignette, participants were asked to rate two things:

(*i*) how much they felt what the protagonist was feeling, i.e. how much pain/anxiety/fear/embarrassment they were feeling (our measure of empathy);

(*ii*) how much compassion they felt for the protagonist. Ratings were provided on separate sliding scales that ranged from 0 ("*None at all*") to 100 ("*The most you could imagine ever feeling on another's behalf*").

Empathy and compassion scores were computed for each participant by averaging their responses across the six scenarios and had good internal reliability scores (Miller & Cushman, 2017).

Moral judgment task

These participants were re-contacted and asked to provide moral judgments on vignettes, which consisted of four variations of 4 unique scenarios for a total of 16 stories. The four variations were result of a 2 × 2 within-subjects design. Each participant saw only one variation of each scenario, for a total of 4 stories. The order of presentation of scenarios was randomized across subjects.

After reading each scenario, participants provided ratings of four questions (presented on the same screen) on a 7-point Likert scale:

(*i*) *wrongness*: How wrong was [the agent]'s behavior? (1: *Not at all*, 4: *Somewhat*, 7: *Very much*);

(*ii*) *punishment*: How much should [the agent]'s be punished? (1: *None at all*, 4: *Some*, 7: *A lot*)

Results

- *Empathy*:

Linear mixed-effects regression

We carried out linear mixed-effects regression (random slopes for item and type of question and random intercept for participant) separately for each condition to see if there was a positive association between ECT-empathy and severity of judgment. R syntax for each condition was the following:

```
lmer(rating ~ Empathy_ECT + (1 + Empathy_ECT | item) + (1 +
Empathy_ECT | question) + (1 | id), data = subset(data_file),
condition = "condition of interest"))
```

Although the results revealed a marginally significant effect for accidental harm condition in the expected direction, the 95% percentile confidence interval (from 1000 bootstrap samples) for the estimate did not include 0.

**condition**	**estimate**	**95% percentile ci**		**se**	***df***	***t* value**	***p*-value**
		**LB**	**UB**				
neutral	-0.002	-0.010	0.005	0.004	108.590	-0.631	0.529
accidental	0.012	0.001	0.023	0.006	8.496	2.105	0.066
attempted	0.002	-0.011	0.014	0.007	27.375	0.297	0.769
intentional	-0.009	-0.018	0.000	0.005	29.090	-1.740	0.092

Ordinal mixed-effects regression

Since our data were discrete (1–7 Likert scale) rather than continuous, we additionally carried out ordinal mixed effects regression (specifically, we fit a cumulative link mixed model) as an additional robustness check (R package: `ordinal`). The syntax used was the following-

```
clmm(rating ~ Empathy_ECT + (1 + Empathy_ECT | item) + (1 | id),
data = subset(dataset, condition == "condition of interest"))
```

No random effect for question was included given that it has less than 3 levels.

condition	estimate	se	*z*	*p* -value
neutral	-0.003	0.027	-0.114	0.909
accidental	0.024	0.012	2.074	0.038
attempted	0.000	0.009	0.048	0.961
intentional	-0.019	0.013	-1.440	0.150

Thus, we found evidence for the empathic blame hypothesis with this new measure of state empathy: individuals scoring high on this measure condemned accidents harshly.

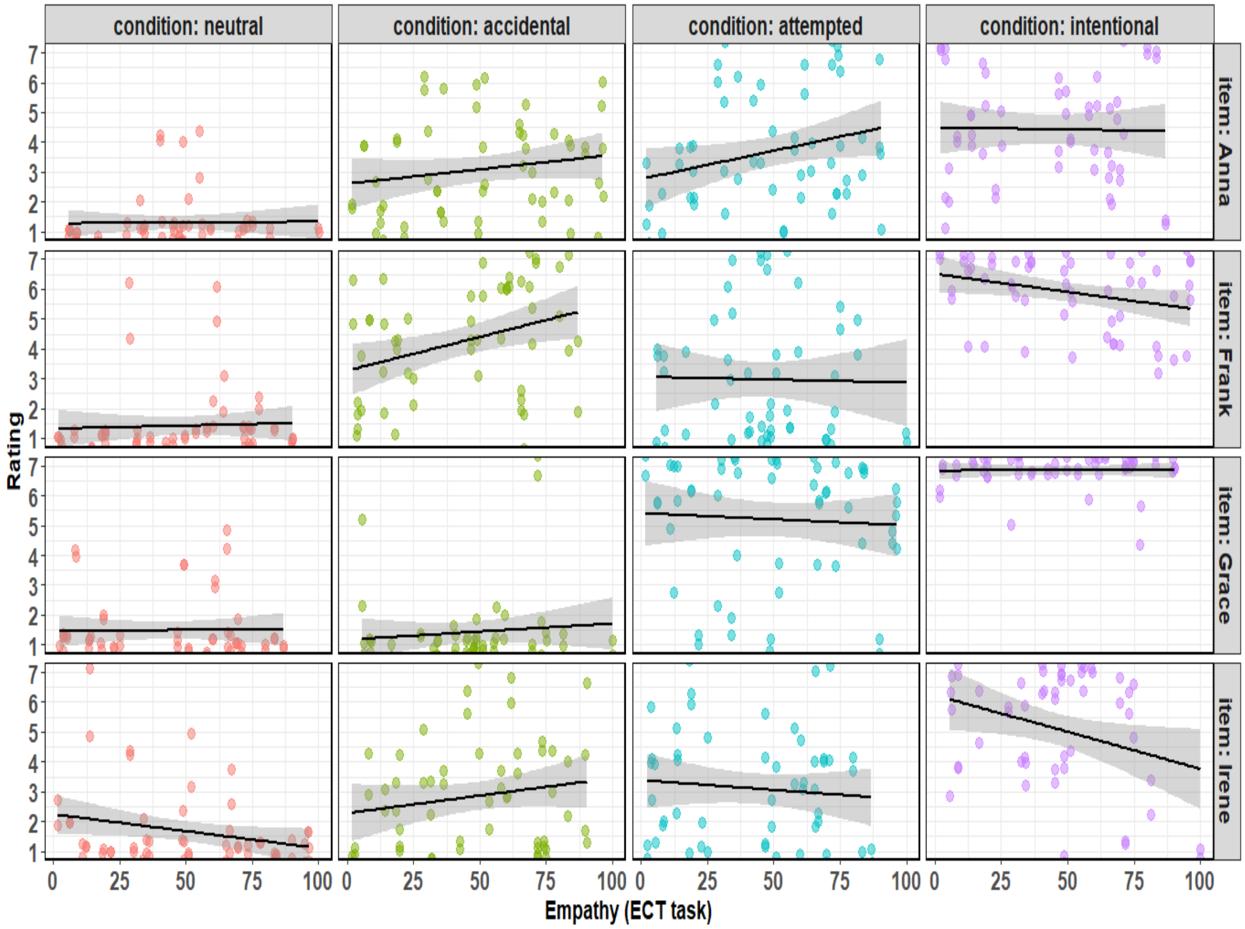

- *Compassion*:

Linear mixed-effects regression

In exploratory analysis, we also carried out linear mixed-effects regression (random slopes for item and type of question and random intercept for participant) separately for each condition to see if there was any association between ECT-compassion/sympathy and severity of moral condemnation. R syntax for each condition was the following:

```
lmer(rating ~ Sympathy_ECT + (1 + Sympathy_ECT | item) + (1 + Sympathy_ECT | question) + (1 | id), data = subset(data_file), condition = "condition of interest"))
```

There were no significant results. More importantly, as would be predicted by the empathic blame hypothesis, there was no relationship between the (state feelings of) compassion participants felt and the tendency to condemn the accidental harm-doer based on the harmful outcome.

condition	estimate	95% percentile ci		se	*df*	*t* value	*p*-value
		LB	UB				
neutral	-0.010	-0.021	0.000	0.005	5.110	-1.943	0.108
accidental	0.001	-0.013	0.015	0.007	4.260	0.196	0.854
attempted	0.007	-0.008	0.022	0.008	28.762	0.920	0.365
intentional	0.001	-0.013	0.015	0.007	9.116	0.146	0.887

Ordinal mixed-effects regression

Since our data were discrete (1–7 Likert scale) rather than continuous, we additionally carried out ordinal mixed effects regression (specifically, we fit a cumulative link mixed model) as an additional robustness check (R package: `ordinal`). The syntax used was the following-

```
clmm(rating ~ Sympathy_ECT + (1 + Sympathy_ECT | item) + (1 | id), data = subset(dataset, condition == "condition of interest"))
```

No random effect for question was included given that it has less than 3 levels.

outcome	estimate	se	z	*p* -value
neutral	-0.017	0.009	-1.877	0.061
accidental	0.003	0.019	0.177	0.860
attempted	0.007	0.012	0.590	0.555
intentional	-0.006	0.018	-0.335	0.737

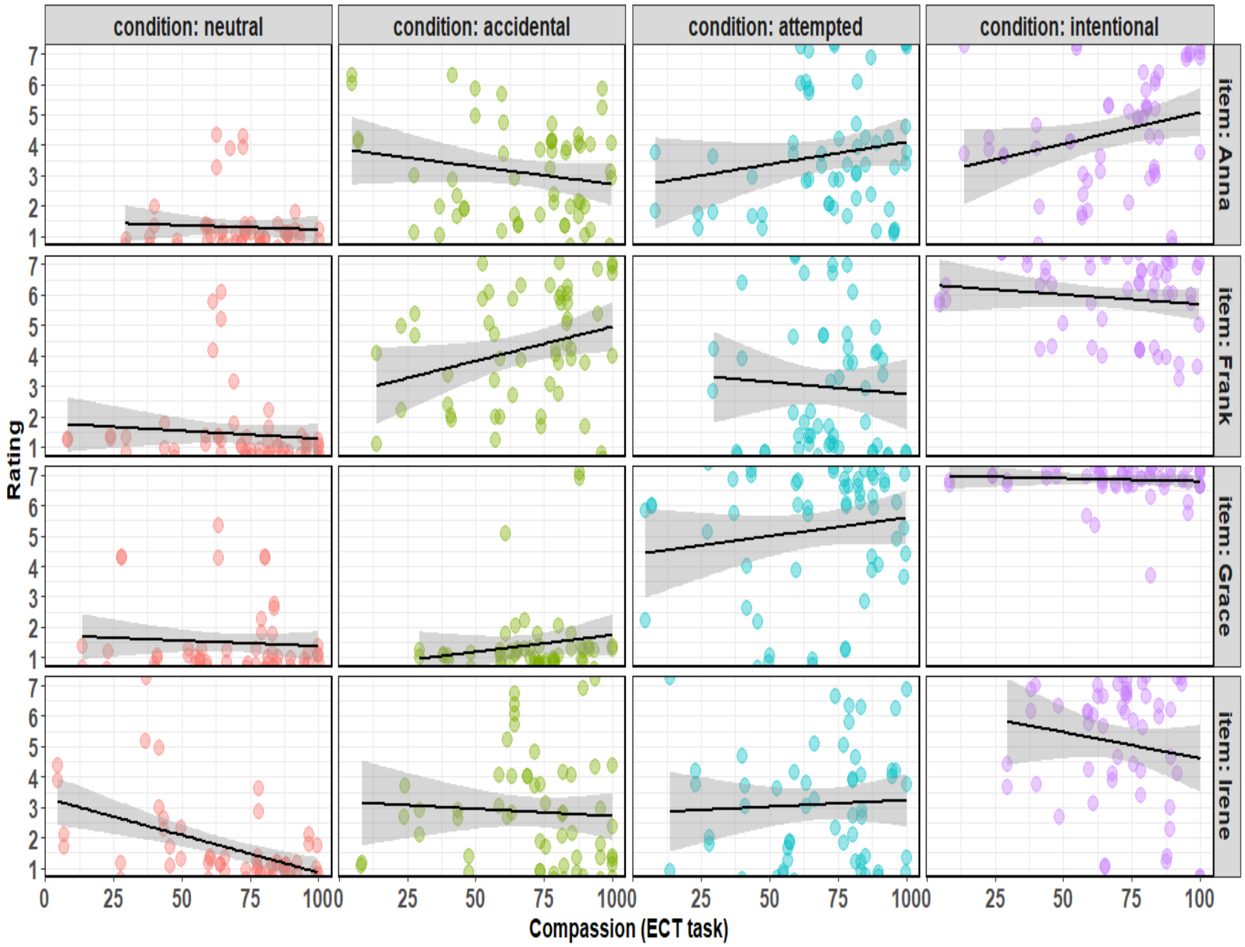

Supplementary Text 5: Trial-by-trial victim suffering ratings (Study 4)

In the current study, we assessed if empathic response when confronted with specific situation can also predict harshness of moral judgments. This would provide further support for the empathic blame hypothesis.

Participants

Twenty-three healthy control subjects (six women, 17 men, M_{age} = 34.4, age range: 17-66 years) were recruited. All participants took part voluntarily, gave written informed consent, and were monetarily compensated for their time and travel expenses. The study was approved by the Ethics Committee of the University of Vienna and conducted in accordance with the declaration of Helsinki.

Methods and Materials

Experimental stimuli consisted of four variations of 20 unique scenarios for a total of 80 stories. All scenarios were adapted in German. The four variations were result of a 2 (belief: neutral, negative) × 2 (outcome: neutral, negative) within-subjects design. Each participant saw only one variation of each scenario, for a total of 20 stories. Each scenario lasted for as long as the participant needed to reduce working memory load and consisted of four cumulative segments. The order of presentation of scenarios was randomized across subjects.

After reading each scenario, participants provided four types of moral judgments:

(*i*) *acceptability*: “How morally acceptable was [the agent]’s behavior?” (0 = *not at all acceptable*; 20 = *completely acceptable*);

(*ii*) *blame*: “How much blame does [the agent] deserve?” (0 = *none at all*, 20 = *very much*);

(*iii*) *suffering*: "How much do you think [the victim] suffered?" (0 = *not at all*; 20 = *extreme suffering*);

(*iv*) *emotional arousal*: “How emotionally arousing did you find this scenario?” (0 = *not at all arousing*; 20 = *extremely arousing*).

The first two judgments ("acceptability" and "blame") were presented in random order, but "suffering" was always at third and "emotional arousal" always at fourth place. After each story was presented, participants responded using a computerized visual analog scale (VAS), implemented as horizontal on-

screen bar. A gray bar indicated the total range of possible responses, while a slightly narrower black asterisk included in the gray bar indicated the participants' current response. The position of the asterisk could be altered by using the "←" and "→" arrows on the keyboard. This scale was anchored with the labels mentioned before. Participants' responses were later converted to standardized scores with [min, max] of [0, 20]. The acceptability scores were reverse-scored to have a positive association with blame scores (higher score = less acceptable behavior and more blame). There was no time limit to respond to any of the questions. The response buttons were active for as long as the question remained on the screen and so participants could move the asterisk to one position and change it to another until they moved on to the next question by pushing the "space bar".

Results

The descriptive statistics for all ratings are provided below:

Question	Condition	*M*	*SD*	Min	Max
Acceptability	accidental	9.03	5.06	0.26	18.66
	attempted	13.99	3.67	7.66	20.00
	intentional	17.87	2.39	12.08	20.00
	neutral	2.21	1.91	0.00	6.05
Blame	accidental	8.79	5.21	0.13	19.14
	attempted	13.69	3.91	6.94	20.00
	intentional	17.96	2.54	11.54	20.00
	neutral	2.21	1.87	0.00	6.06
Suffering	accidental	16.61	2.54	10.90	20.00
	attempted	1.44	2.01	0.00	8.99
	intentional	17.33	2.33	12.77	20.00
	neutral	0.80	1.09	0.00	3.60
Arousal	accidental	8.11	5.61	0.00	17.50
	attempted	5.46	4.10	0.03	12.98
	intentional	8.96	5.91	0.00	19.33
	neutral	2.55	2.87	0.00	9.87

Since the acceptability and blame judgments were highly correlated, they were averaged to get a unique moral condemnation[1] score for each participant (Cronbach's alphas: neutral = 0.885, accidental = 0.963, attempted = 0.933, intentional = 0.950).

Hierarchical regression analyses determined whether emotional arousal or victim suffering predicted moral condemnation once age and gender were controlled for, and whether each could predict the dependent variables after the other was controlled for. As noted in a previous study (Brewer et al., 2015), it is necessary to perform hierarchical regressions with correlated predictor variables entered in both possible orders to independently investigate the effect of each, after controlling for the other, because of collinearity. We provide here results only for the order (suffering first, arousal second), although the same result was observed in the other order (arousal first, suffering second).

Note: All *p*-values are two-tailed and effects of primary interest are highlighted in bold.

**Step**	**Predictor**	**accidental harm**				
		β	t	p	ΔR^2	p for ΔR^2
1	(Constant)		2.524	0.020	0.015	0.858
	gender	0.028	0.116	0.908		
	age	0.111	0.465	0.647		
2	(Constant)		-1.599	0.126	0.284	**0.012**
	gender	0.035	0.168	0.868		
	age	0.221	1.056	0.304		
	suffering	0.545	2.777	0.012		
3	(Constant)		-1.462	0.161	0.090	0.120
	gender	-0.082	-0.392	0.700		
	age	0.245	1.217	0.239		
	suffering	**0.430**	**2.136**	**0.047**		
	arousal	0.338	1.630	0.120		

[1] Similar results are observed even if the regression analyses are run independently for acceptability and blame judgments without combining them to form a unique moral condemnation score.

Step	Predictor	intentional harm				
		β	t	p	ΔR^2	p for ΔR^2
1	(Constant)		13.642	< 0.001	0.231	0.072
	gender	0.466	2.220	0.038		
	age	0.036	0.173	0.864		
2	(Constant)		3.034	0.007	0.146	**0.048**
	gender	0.410	2.096	0.050		
	age	0.017	0.087	0.932		
	suffering	0.388	2.115	0.048		
3	(Constant)		3.290	0.004	0.056	0.200
	gender	0.541	2.509	0.022		
	age	-0.041	-0.211	0.836		
	suffering	**0.405**	**2.242**	**0.038**		
	arousal	-0.267	-1.330	0.200		

Similar analysis for the neutral cases and attempted harm did not reveal any significant predictors of interest ($p > 0.05$).

Step	Predictor	neutral case				
		β	t	p	ΔR^2	p for ΔR^2
1	(Constant)		2.190	0.041	0.028	0.750
	gender	-0.176	-0.744	0.466		
	age	0.024	0.102	0.920		
2	(Constant)		2.426	0.025	0.099	**0.159**
	gender	-0.122	-0.527	0.605		
	age	0.063	0.273	0.788		
	suffering	-0.323	-1.464	0.159		
3	(Constant)		2.537	0.021	0.195	0.035
	gender	-0.154	-0.728	0.476		
	age	-0.087	-0.394	0.698		
	suffering	**-0.271**	**-1.349**	**0.194**		
	arousal	0.470	2.273	0.035		

Step	Predictor	attempted harm				
		β	t	p	ΔR^2	p for ΔR^2
1	(Constant)		6.156	< 0.001	0.052	0.587
	gender	0.210	0.899	0.379		
	age	0.041	0.175	0.863		
2	(Constant)		6.116	< 0.001	0.047	**0.330**
	gender	0.104	0.406	0.689		
	age	0.048	0.207	0.838		
	suffering	-0.241	-1.000	0.330		
3	(Constant)		5.547	< 0.001	0.006	0.730
	gender	0.081	0.300	0.768		
	age	0.048	0.199	0.845		
	suffering	**-0.270**	**-1.038**	**0.313**		
	arousal	0.083	0.351	0.730		

Thus, as can be garnered from the regression coefficients, after accounting for shared variance, victim suffering, and not emotional arousal, emerged as the significant predictor of moral judgments: subjects who reported greater perceived suffering in the victims of the accidental and intentional harms also judged these cases more severely in moral terms. Additionally, in none of the models, arousal ratings explained any additional variance once variance associated with suffering was accounted for.

This study provides further evidence for the empathic blame hypothesis as it shows that trial-by-trial rating of the victim's suffering predicts how much the third-party judge condemns the agent who accidentally or intentionally harmed someone. It is important to note that the relationship observed in Study 1 between empathic concern and attempted harms is no longer observed with the improved measure we used here that assesses victim suffering specifically rather than indexing a general, dispositional concern for others' wellbeing. This is in line with the empathic blame hypothesis.

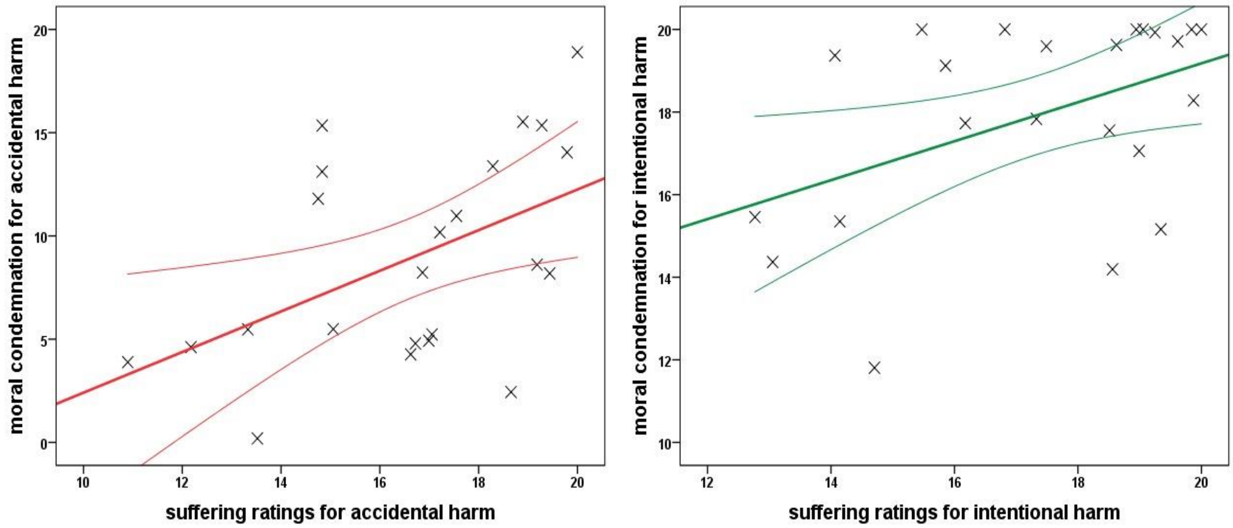

Limitations

Although participants provided their assessments of how much the victim suffered, it is not clear if their assessment stemmed from vicariously sharing victim's feelings (which would indeed be "empathy") or by explicitly reasoning and drawing inferences about their mental and feeling states (which would be mentalizing or cognitive empathy; Zaki & Ochsner, 2012). We sidestep these shortcomings of self-report measures in the neuroimaging study by using neural activity extracted from empathy network nodes, localized independently, and see if a similar association is observed.

Supplementary Text S6: Empathy and compassion for the agent versus the victim (Study 5)

In this study, we provide evidence to support the oft-made - but rarely empirically supported - claim that while evaluating third-party harmful behaviors, the judges empathize with the victims and not with the perpetrators.

Participants

Eighty participants (26 females) were recruited through Amazon Mechanical Turk and all provided informed consent. Level of education varied widely (from high school to PhD) and so did age, political

orientation, and religiosity. All participants received monetary compensation ($1) for their participation and all procedures were approved by the Harvard University Institutional Review Board.

Methods and Materials

To make sure that we were not committing a nomothetic fallacy (Meindl & Graham, 2014) whereby we were using the terms “empathy” and “compassion” in a different way than the participants, we first provided participants with text illustrating the way we were defining these terms (see Supplementary Text S4).

After reading these instructions, participants were shown moral vignettes which consisted of four variations of 4 unique scenarios for a total of 16 stories. The four variations were result of a 2 (belief: neutral, negative) × 2 (outcome: neutral, negative) within-subjects design. Each participant saw only one variation of each scenario, for a total of 4 stories. The order of presentation of scenarios was randomized across subjects.

After reading each scenario, participants provided ratings of four questions (presented on the same screen) on a 7-point Likert scale (1: *None at all*, 4: *Somewhat*, 7: *A lot*):

(*i*) *agent empathy*: “After reading this scenario, how much empathy did you feel for [the agent]? In other words, to what degree did you feel what this person was feeling.”;

(*ii*) *agent compassion*: “After reading this scenario, how much compassion did you feel for [the agent]? In other words, how much were you concerned about this person's well-being.”;

(*iii*) *victim empathy*: “After reading this scenario, how much empathy did you feel for [the victim]? In other words, to what degree did you feel what this person was feeling.”;

(*iv*) *victim compassion*: “After reading this scenario, how much compassion did you feel for [the victim]? In other words, how much were you concerned about this person's well-being.”;

Results

The descriptive statistics for all ratings are provided below:

condition	belief	outcome	question	focus	N	mean	sd	se	ci
intentional	negative	negative	compassion	agent	80	2.000	1.691	0.189	0.376
	negative	negative	compassion	victim	80	5.900	1.658	0.185	0.369
	negative	negative	empathy	agent	80	1.988	1.673	0.187	0.372
	negative	negative	empathy	victim	80	4.950	2.086	0.233	0.464
attempted	negative	neutral	compassion	agent	80	2.188	1.669	0.187	0.372
	negative	neutral	compassion	victim	80	4.200	2.071	0.232	0.461
	negative	neutral	empathy	agent	80	2.125	1.702	0.190	0.379
	negative	neutral	empathy	victim	80	3.700	2.071	0.232	0.461
accidental	neutral	negative	compassion	agent	80	3.963	2.281	0.255	0.508
	neutral	negative	compassion	victim	80	5.675	1.727	0.193	0.384
	neutral	negative	empathy	agent	80	3.463	2.025	0.226	0.451
	neutral	negative	empathy	victim	80	4.713	2.045	0.229	0.455
neutral	neutral	neutral	compassion	agent	80	2.688	1.832	0.205	0.408
	neutral	neutral	compassion	victim	80	3.025	2.129	0.238	0.474
	neutral	neutral	empathy	agent	80	2.625	1.694	0.189	0.377
	neutral	neutral	empathy	victim	80	2.825	2.042	0.228	0.455

The density plot for the ratings by condition by question is shown below. As can be seen, for attempted and intentional harm conditions, there was neither empathic nor compassionate response towards the agent. On the other hand, there was strong empathic reaction and compassion for the victim of the harmful act. For the most interesting condition for the current study, the accidental harm condition, the response was mixed: Although people empathized with and felt compassion for the victim more than the agent, this response was significant nonetheless (one sample t-test: *p*s < 0.001).

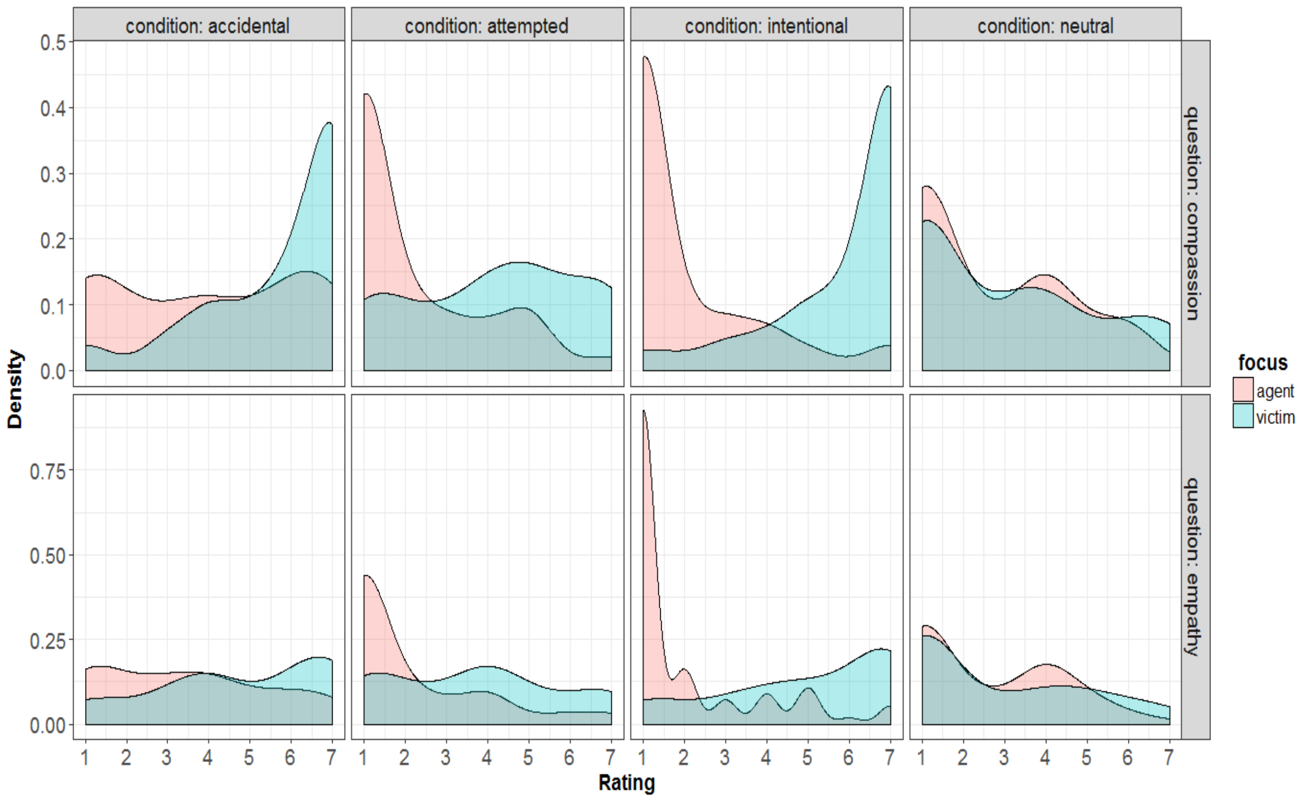

The linear mixed-effects regression model predicting ratings from the fixed effects of belief (neutral, negative), outcome (neutral, negative), question (empathy, compassion), and focus (agent, victim) and the random intercepts for item and participant:

```
lmer(rating ~ belief*outcome*question*focus + (1 | item) + (1|
id), data = emp_comp_long, REML = FALSE, control =
lmerControl(optimizer = "Nelder_Mead", restart_edge = TRUE,
boundary.tol = 1e-5, calc.derivs = TRUE, use.last.params = FALSE,
optCtrl = list(maxfun = 2e7)))
```

Of interest to us, people had more empathy (estimate = -1.2500, se = 0.3021, t = -4.138, p < 0.01) and compassion (estimate = -1.7125, se = 0.3021, t = -5.668, p < 0.01) for the victim than the agent.

Fixed effect	estimate	*df*	se	*t*	*p*
(Intercept)	1.997	0.239	55.290	8.361	0.000
belief (neutral)	1.972	0.258	1196.859	7.647	0.000
outcome (neutral)	0.189	0.258	1196.873	0.733	0.464
question (empathy)	-0.013	0.258	1196.832	-0.048	0.961
focus (victim)	3.900	0.258	1196.832	15.125	0.000
belief (neutral) : outcome (neutral)	-1.471	0.365	1196.859	-4.035	0.000
belief (neutral) : question (empathy)	-0.487	0.365	1196.832	-1.337	0.182
outcome (neutral) : question (empathy)	-0.050	0.365	1196.832	-0.137	0.891
belief (neutral) : focus (victim)	-2.187	0.365	1196.832	-5.999	0.000
outcome (neutral) : focus (victim)	-1.887	0.365	1196.832	-5.176	0.000
question (empathy) : focus (victim)	-0.937	0.365	1196.832	-2.571	0.010
belief (neutral) : outcome (neutral) : question (empathy)	0.487	0.516	1196.832	0.945	0.345
belief(neutral) : outcome (neutral) : focus (victim)	0.512	0.516	1196.832	0.994	0.321
belief (neutral) : question (empathy) : focus (victim)	0.475	0.516	1196.832	0.921	0.357
outcome (neutral) : question (empathy) : focus (victim)	0.500	0.516	1196.832	0.970	0.332
belief (neutral) : outcome (neutral) : question (empathy) : focus (victim)	-0.175	0.729	1196.832	-0.240	0.810

The least squares means (lsmean) only for empathy question, which was of interest to us, are shown below. Note that participants reported to have felt empathy for victim even in cases where there was no harmful outcome (neutral and attempted cases, i.e.).

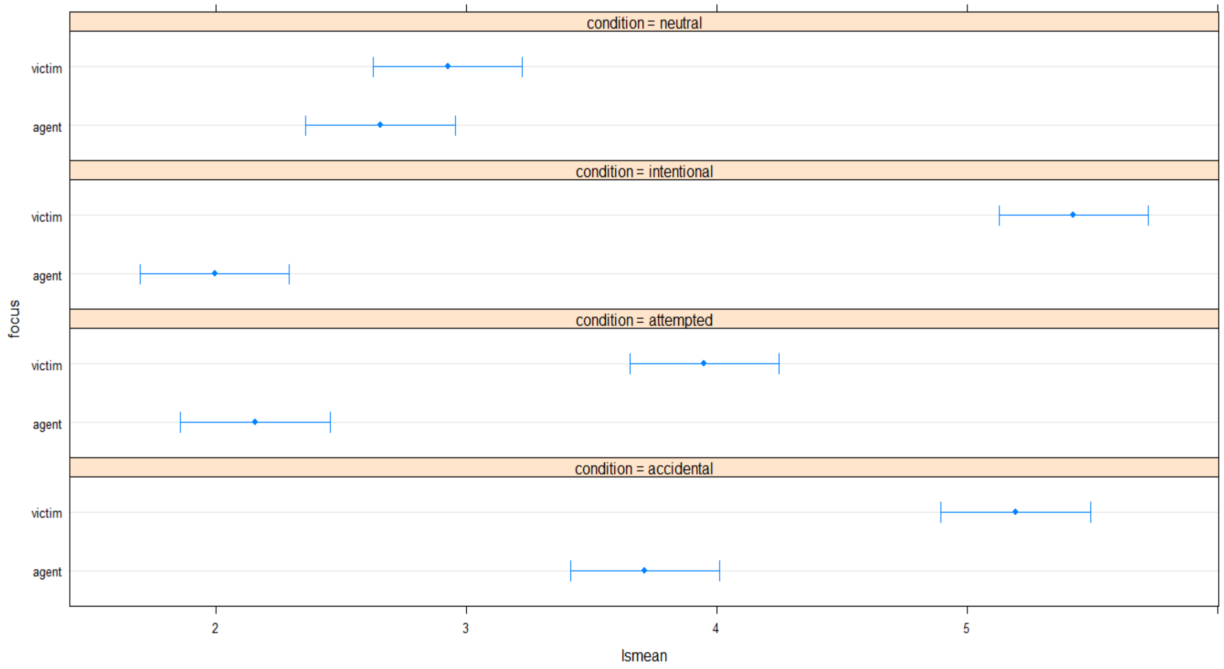

Similar analysis on compassion ratings revealed a similar pattern where participants felt more compassion for the victim (versus agent):

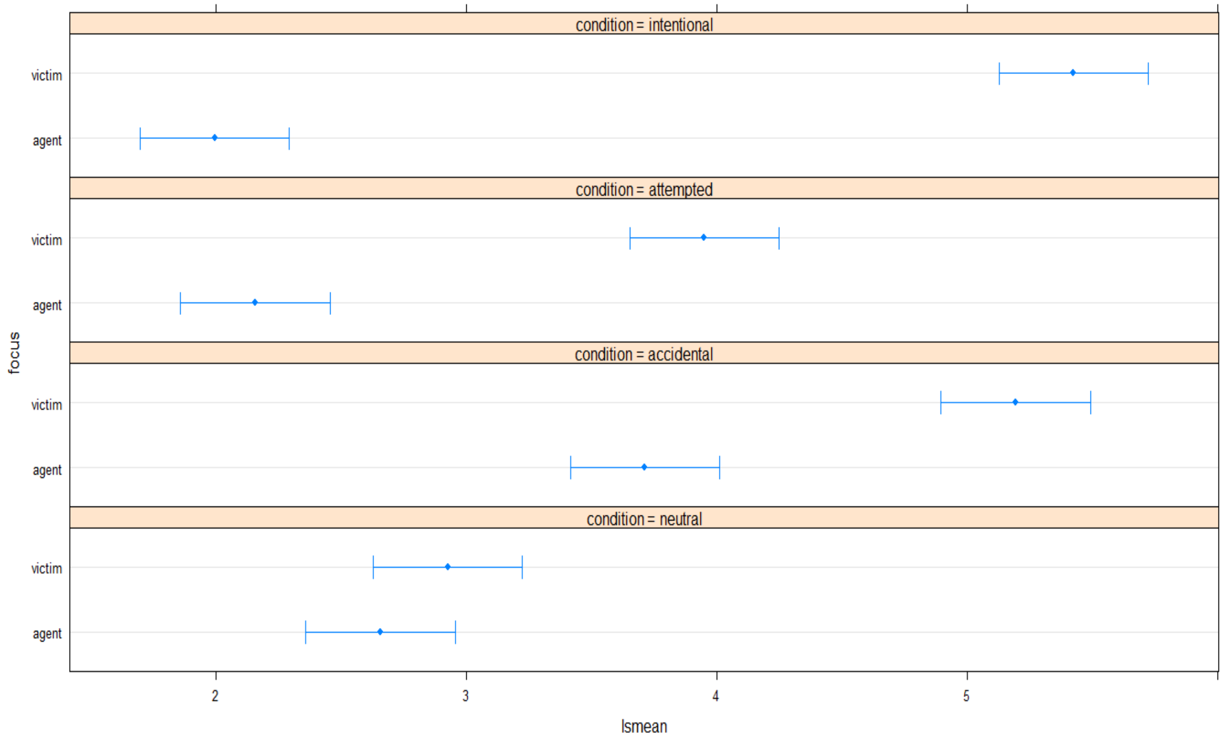

Supplementary Text 7: Outcome-based punishment vs intent-based wrongness (Study 6)

Participants

Eighty-five undergraduates at Brown University completed the task in lab settings for course credit and all provided informed consent. Participation was restricted to native English speakers, although no restriction was imposed regarding US citizenship. All procedures were approved by the Brown University Institutional Review Board.

Methods and Materials

This study employed a mixed design with the following factors: group (wrongness, punishment, or both) × type of judgment (wrongness, punishment) × intent (neutral, negative) × outcome (neutral, negative). Thus, group defined the between-subjects factor: one group of participants provided *only wrongness*

judgments for all stories, another provided *only punishment* judgment, while the third group provided *both* judgments *across* stories (i.e., wrongness for few, punishment for other).

Stimuli consisted of four variations of 48 unique scenarios for a total of 192 stories, taken from previous work (Young, Camprodon, et al., 2010). The story-by-judgment combination was counterbalanced across subjects by creating two different versions of the scenarios. Each participant saw one variation of each scenario, for a total of 48 stories (12 per condition). After each story, participants provided *either* of the two evaluations:

- For wrongness judgment, the instruction was "For each story, you will rate how wrong the protagonist's behavior was, from permissible to forbidden." and the rating was collected on a 7-point Likert scale (1: *permissible*, 7: *forbidden*).
- For punishment judgment, the instruction was "You will rate how much the protagonist should be punished, on a scale from no punishment at all to very much punishment." and the rating was collected on a 7-point Likert scale (1: *none at all*, 7: *very much*).

Results

Descriptive statistics based on estimated marginal means are tabulated below:

**Intent**	**Outcome**	**Type of judgment**	**Mean**	**95% CI**	
				**LB**	**UB**
neutral	neutral	punishment	1.662	1.239	2.085
		wrongness	1.957	1.534	2.380
	negative	punishment	3.472	3.049	3.895
		wrongness	3.661	3.239	4.084
negative	neutral	punishment	4.027	3.604	4.450
		wrongness	5.038	4.615	5.460
	negative	punishment	5.872	5.449	6.295
		wrongness	6.199	5.776	6.622

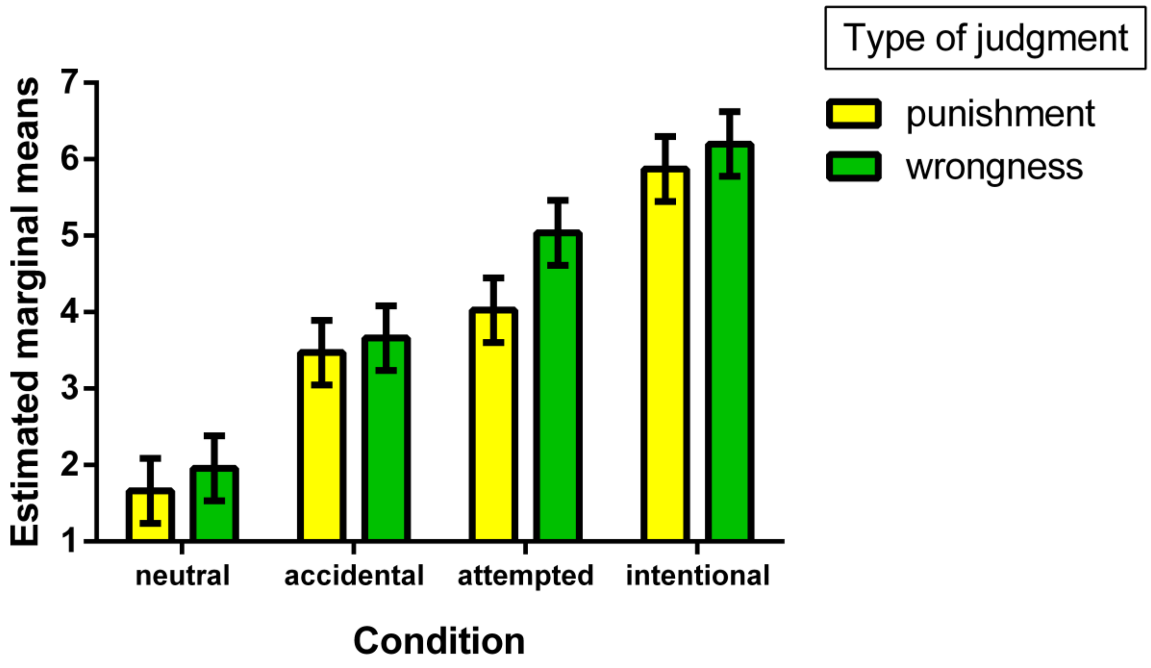

Note: The error bars represent 95% confidence interval.

We used linear mixed-effects model that included fixed effects for the factors intent, outcome, and type of judgment (all within-subjects factors), as well as all possible two-way interactions and the three-way interactions. We included random intercepts for group, participant, scenario, and version of the test, as well as random slopes for two-way interaction of each of these factors with within-subject factors. Thus, as recommended for confirmatory hypothesis testing (Barr, Levy, Scheepers, & Tily, 2013), the model included the maximal random effects structure. Maximum Likelihood (ML) estimation and variance components covariance structure were used.

Fixed effects	df_1	df_2	F	p
belief	1	93.632	390.86	< 0.001
outcome	1	97.366	257.224	< 0.001
type of judgment	1	2.899	23.158	0.018
belief × outcome	1	3704.29	7.36	0.007
belief × type of judgment	1	2923.29	15.171	< 0.001
outcome × type of judgment	1	2806.12	13.077	< 0.001
belief × outcome × type of judgment	1	3716.28	9.471	0.002

As expected, main effect of intent and outcome and their interaction were all significant. Of primary interest to us, the interaction between type of outcome (neutral versus negative) and type of judgment (wrongness versus punishment) was significant. Indeed, the *post hoc* comparisons revealed that the increase in punishment for negative outcomes over neutral outcomes (mean difference = 1.828, 95% CI [1.599, 2.057], df = 155.179, $p < 0.001$) was higher than the same difference for wrongness judgments (mean difference = 1.433, 95% CI [1.206, 1.660], df = 150.646, $p < 0.001$).

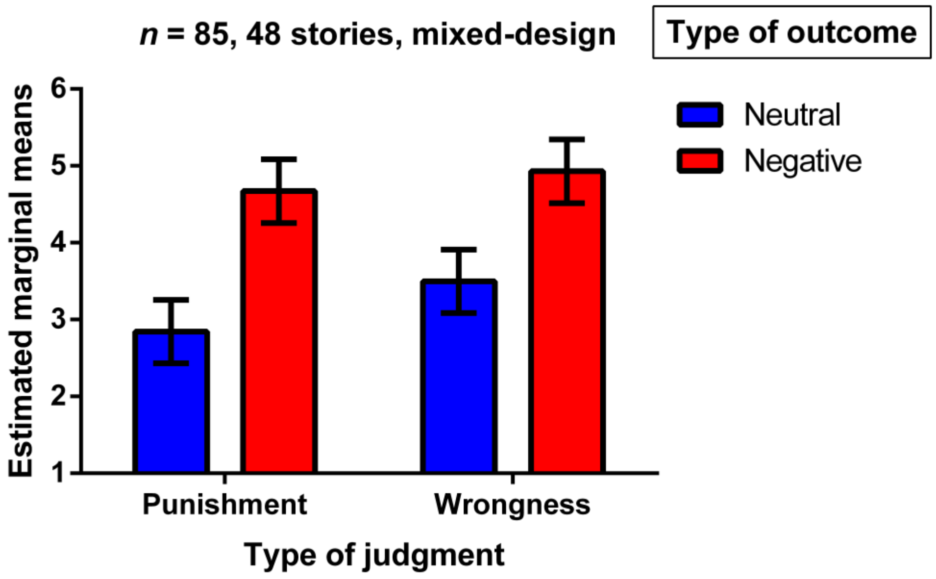

Note that there was also a significant belief-by-judgment interaction, which is indicative of the greater role of belief (as assessed by difference between response for negative vs neutral belief condition) for wrongness (mean difference = 2.809, $p < 0.001$) as compared to punishment (mean difference = 2.382, p

< 0.001) judgments. This is a converse of the primary effect of interest: since outcomes matter more for punishment compared to wrongness, the relative contribution of intent is also reduced across these two judgments (more for wrongness as compared to punishment) (Cushman, 2008).

Supplementary Text 8: Outcome-based punishment vs intent-based wrongness (Study 7)

Participants

Thirty-five undergraduates at Brown University completed the task for course credit in a lab-based study and all provided informed consent. Participation was restricted to native English speakers, although no restriction was imposed about US citizenship. All procedures were approved by the Brown University Institutional Review Board.

Methods and Materials

Methods and materials used for this study were identical to previous study with one major change. While in the previous additional study, some participants responded to either punishment or wrongness or punishment *and* wrongness, in the current study each participant faced both types of judgment across stories (wrongness judgments for some stories, while punishment judgments for other). This study thus employed a fully within-subjects design with the following factors: type of judgment (wrongness, punishment) × belief (neutral, negative) × outcome (neutral, negative). The story-by-judgment combination was counterbalanced across subjects by creating two different versions of the scenarios.

Results

Descriptive statistics based on estimated marginal means are tabulated below:

Intent	Outcome	Type of judgment	Mean	95% CI	
				LB	UB
neutral	neutral	punishment	1.341	0.992	1.691
		wrongness	1.638	1.289	1.987
	negative	punishment	3.074	2.725	3.423
		wrongness	3.102	2.753	3.451
negative	neutral	punishment	3.526	3.177	3.875
		wrongness	4.692	4.342	5.041
	negative	punishment	5.669	5.32	6.018
		wrongness	5.995	5.646	6.344

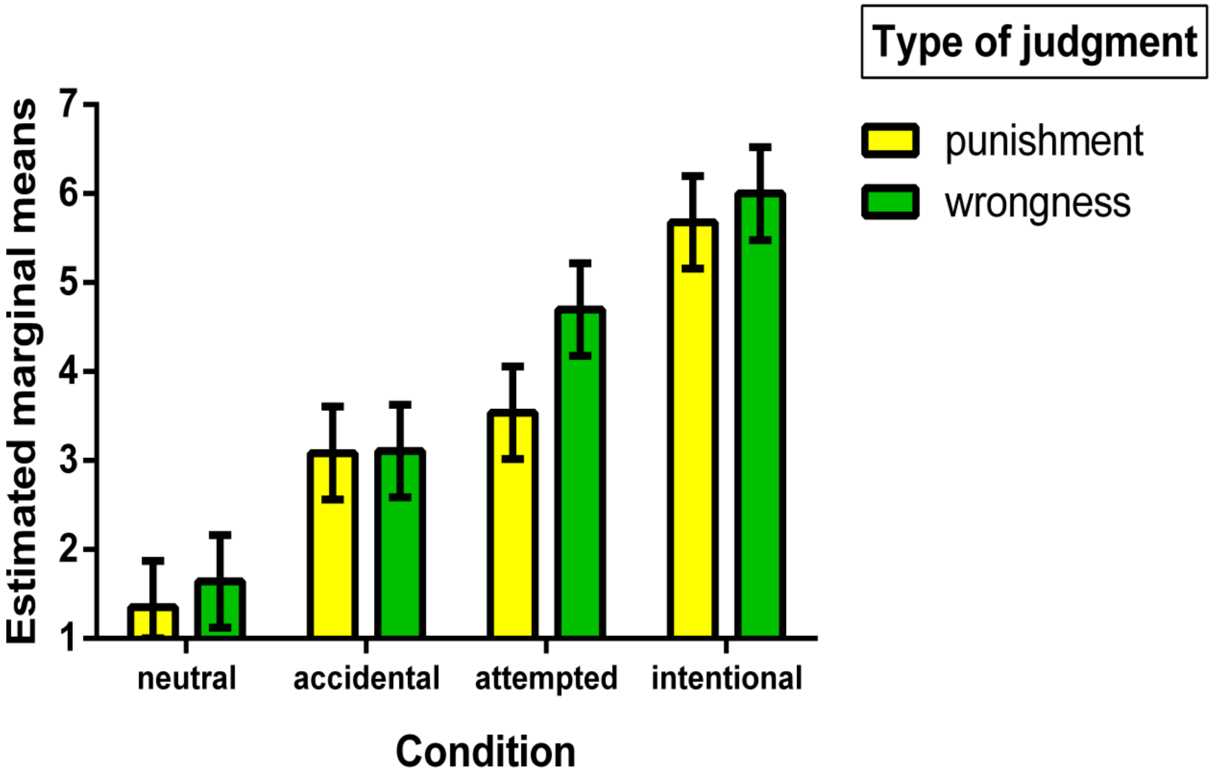

Note: The error bars represent 95% confidence interval.

We used linear mixed-effects model that included fixed effects for the factors belief, outcome, and type of judgment (all within-subjects factors), as well as all possible two-way interactions and the three-way interactions. We included random intercepts for participant, scenario, and version of the test, as well as

random slopes for two-way interaction of each of these factors with within-subject factors. Thus, the model included the maximal random effects structure (Barr et al., 2013). Maximum Likelihood (ML) estimation and variance components covariance structure were used.

**Fixed effects**	df_1	df_2	F	p
belief	1	35.936	249.218	< 0.001
outcome	1	32.968	114.563	< 0.001
type of judgment	1	1.941	17.741	0.055
belief × outcome	1	1232.29	0.78	0.377
belief × type of judgment	1	1228.99	17.062	< 0.001
outcome × type of judgment	1	1217.73	15.35	< 0.001
belief × outcome × type of judgment	1	1228.99	4.11	0.043

Once again, the predicted outcome-by-judgment interaction was observed and the post hoc comparisons revealed that the increase in punishment for harmful outcomes (mean difference = 1.937, 95% CI [1.598, 2.277], *df* = 50.650, *p* < .001) was higher than the same difference for wrongness judgments (mean difference = 1.384, 95% CI [1.045, 1.723], *df* = 50.650, *p* < .001).

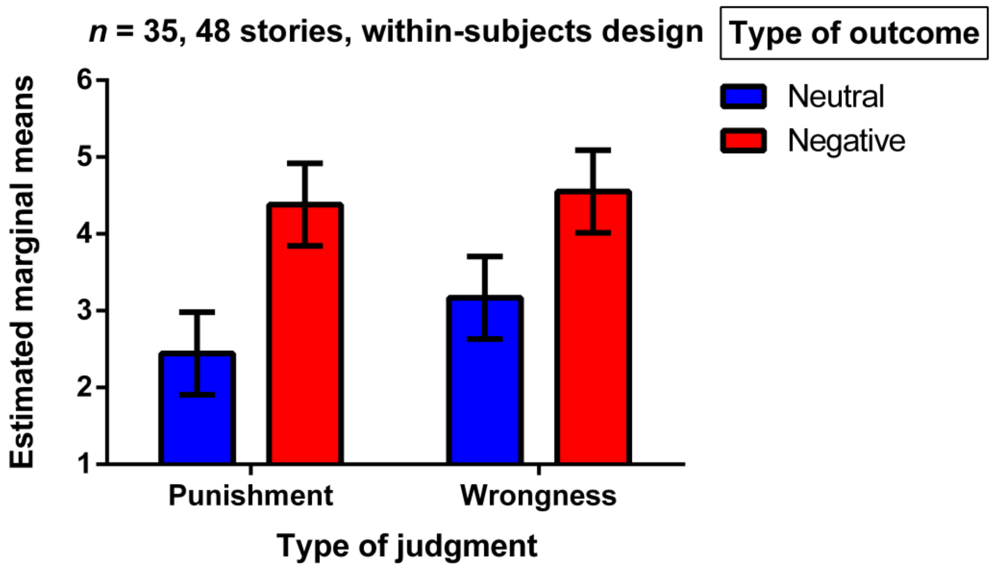

Conclusion:

Thus, across these two studies, we have shown a replication of the moral luck effect both in a within-subjects design (similar, but not identical, to our neuroimaging paradigm) and mixed-design (like prior research). Both studies revealed the expected outcome-by-judgment interaction (*p*s < 0.001), denoting that the presence vs. absences of harmful outcomes weighed more heavily on the punishment (versus acceptability) judgments.

Supplementary Text S9: Affective sharing and moral judgments (Study 8)

Our hypothesis was that punishment judgments rely to a greater degree on empathically encoded harmfulness information than wrongness judgments. Before investigating this hypothesis in an fMRI study, we conducted a proof of principle behavioral study where we predicted that asking participants to share victim's pain would increase punishment more than wrongness of the action, compared to a neutral condition where participants are not explicitly instructed to share victim's pain.

Participants

Six hundred participants (285 females) were recruited through Amazon Mechanical Turk and all provided informed consent. Level of education varied widely (from high school to PhD) and so did age, political orientation, and religiosity. All participants received monetary compensation ($0.10) for their participation and all procedures were approved by the Harvard University Institutional Review Board. Three participants reported to be less than 18 years old and were thus removed ($n = 597$).

Methods and Materials

Participants were randomly assigned to four conditions, which resulted from crossing of two factors: (i) priming: if participants were asked to share victim's pain or not; (ii) question: wrongness or punishment. That is, the study had a 2 (condition: affective sharing instructions, no affective sharing instructions) × 2 (question: wrongness, punishment) fully between-subjects design.

All participants read the following accidental harm scenario:

“*Frank is a dentist filling in the cavity of his patient. He must drill into the patient's tooth just above a major nerve. If the drill is used at a high speed, it will hit the nerve and cause excruciating pain. Franco thinks that if he switches the drill to a higher speed he will dig the cavity without hitting the nerve below. Frank switches the drill to a higher speed, hits the nerve, and causes the patient excruciating pain.*”

After reading this scenario, in priming condition, participants were presented with the following instruction:

“*Now that you have read this situation, try to mirror the feelings of the patient, try to share the patient's pain. Imagine yourself in the patient's position and imagine what would it be like to have your tooth nerve getting drilled into. In other words, try to share the pain the patient must have felt and answer the following question about Frank's action.*”

In the control condition, the participants didn’t receive any such instruction and read the following sentence:

“*Now that you have read this situation, answer the following question about Frank's action*.”

Finally, participants answered one of the following moral judgments (presented with visual analog scale):

1. wrongness (“How wrong was Frank's behavior?”; 0: *Not at all*, 100: *Very much*)

   or

2. punishment (“How much should Frank be punished?”; 0: *None at all*, 100: *A lot*).

Results

Descriptive statistics are tabulated below:

question	condition	N	rating	sd	se	ci
punishment	priming	154	62.357	30.232	2.436	4.813
punishment	control	148	53.365	30.669	2.521	4.982
wrongness	priming	146	73.705	28.314	2.343	4.631
wrongness	control	149	73.329	24.898	2.040	4.031

Density plots reveal that both wrongness and punishment judgments were more densely situated at more severe end of the judgments in the empathy priming condition.

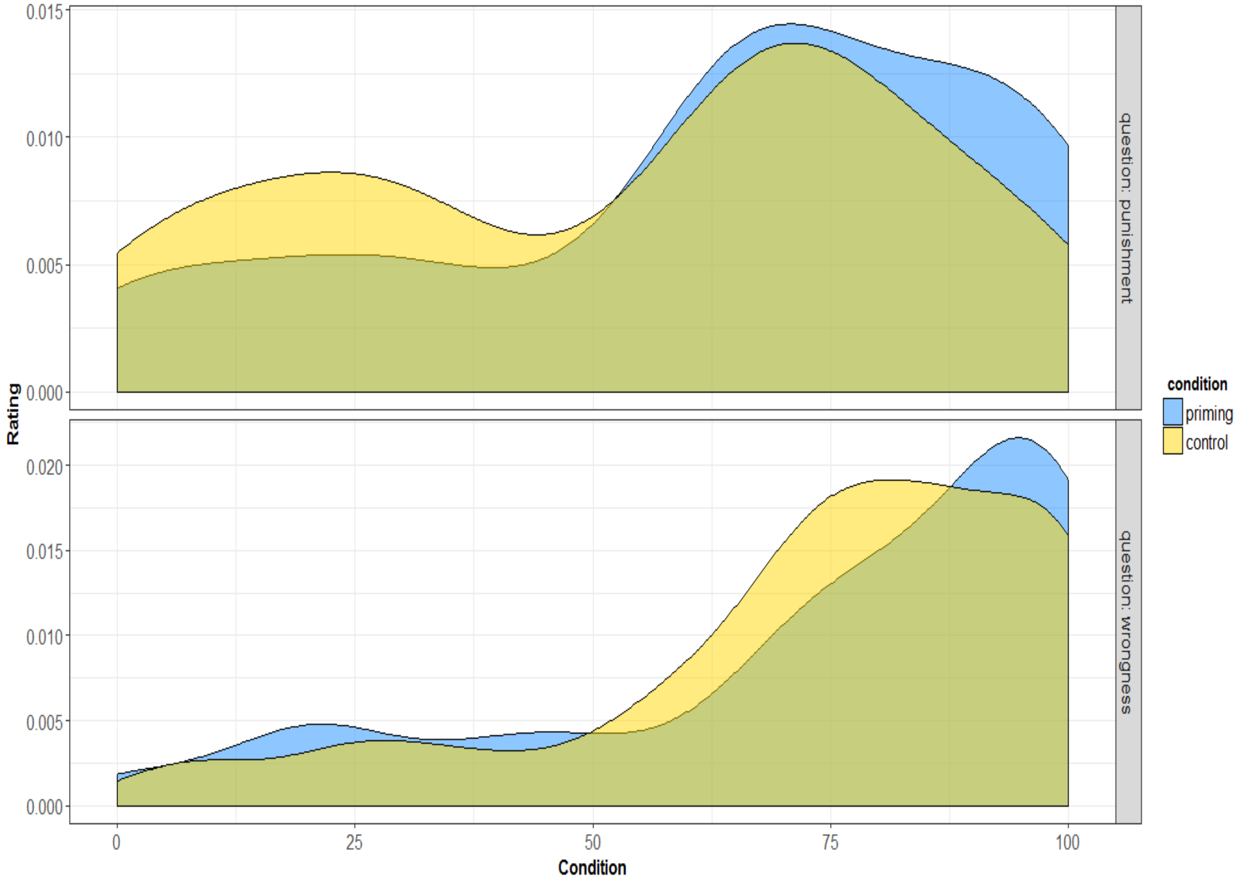

Linear regression revealed that (`lm(rating ~ condition*question)`) there was a main effect of both condition and question, such that participants primed for affective sharing were harsher in their moral judgments, while people were in general more severe in their wrongness (compared to punishment) judgments.

Fixed effects	estimate	se	*t*	*p*
(Intercept)	62.357	2.307	27.028	0.000
condition (control)	-8.992	3.296	-2.729	0.007
question (wrongness)	11.348	3.307	3.431	0.001
condition (control) × question (wrongness)	8.616	4.688	1.838	0.067

More importantly, we also observed a marginally significant interaction effect of condition and question, such that the change in moral judgment due to priming was dependent on the type of question asked. As predicted, post hoc comparisons (`summary(glht(emp_priming_lm, lsm(pairwise ~ condition*question)))`) revealed that this difference was larger for punishment (estimate = 8.9923, se = 3.2957, t = 2.729, p = 0.033) as compared to wrongness (estimate = 0.3766, se = 3.3341, t = 0.113, p = 0.999) judgments.

In other words, asking participants to share victim's pain in accidental harm scenario increased punishment, but left the wrongness judgments unaltered. This result highlights the increased influence of harmful outcomes on punishment as compared to wrongness judgments, mediated via empathic response towards the victim.

Supplementary Text S10: Scenario details

Scenario type by version breakdown. Red and green cells denote scenarios taken from Cushman (2008) and Young, Camprodon, Hauser, Pascual-Leone, & Saxe (2010), respectively.

No.	scenario	v1	v2	v3	v4
1	Popcorn	neu	att	int	acc
2	Malaria Pond/African pond	att	int	acc	neu
3	Spinach	int	acc	neu	att
4	Peanut allergy	acc	neu	att	int
5	Rabies/Rabid dog	neu	att	int	acc
6	Meatloaf	att	int	acc	neu
7	Seatbelt/Amusement park	int	acc	neu	att
8	Teenagers/Skiing	acc	neu	att	int
9	Ham sandwich	neu	att	int	acc
10	Safety Cord/Rock climbing	att	int	acc	neu
11	Sesame seeds	int	acc	neu	att
12	Coffee/Chemical Plant	acc	neu	att	int
13	Bridge	neu	att	int	acc
14	Pool	att	int	acc	neu
15	Mushrooms	int	acc	neu	att
16	Latex	acc	neu	att	int
17	Motorboat	neu	att	int	acc
18	Asthma	att	int	acc	neu
19	Veterinarian/Dog poison	int	acc	neu	att
20	Zoo	acc	neu	att	int
21	Sushi	neu	att	int	acc
22	Cayo/Monkeys	att	int	acc	neu
23	Wet floor	int	acc	neu	att
24	Lab	acc	neu	att	int
25	Vitamin	neu	att	int	acc
26	Airport	att	int	acc	neu
27	Chairlift	int	acc	neu	att
28	Bike	acc	neu	att	int
29	Safety Town/Fire drill	neu	att	int	acc
30	Parachute	att	int	acc	neu
31	Sculpture	int	acc	neu	att
32	Dentist	acc	neu	att	int
33	Iron	neu	att	int	acc
34	Tree House	att	int	acc	neu
35	Jellyfish/Ocean	int	acc	neu	att
36	Laptop	acc	neu	att	int

Note: The exact wording of the details can be found in Appendix at the end of this document[2]. Italian translations are also available on request.

[2] Included here on recommendation by one of the reviewers.

Additional details on nature of stimuli used:

When faced with possible harmful situations, human judges tend to perceive them in terms of a moral dyad consisting of (*a*) a moral agent with capacity for purposeful action and goal-directed behavior who is attributed *moral responsibility* for intending to cause or causing harm and (*b*) a moral patient/victim with capacity for sensations and feelings and is attributed *moral rights* that need to be defended (Gray & Schein, 2012; Theriault & Young, 2014). In other words, while assessing behavior of a perpetrator, judges need to simulate both epistemic (beliefs, knowledge, desires, etc.) and feeling (pain, suffering, etc.) states in others.

But we note that intent and harmfulness inputs represent *sufficient* but not *necessary* inputs to moral judgment (Inbar, Pizarro, & Cushman, 2012). Several other factors that have been shown to influence moral judgments about third-party violations were held constant across scenarios. In none of the scenarios, victims were responsible for their own fate since such scenarios tend to elicit reduced empathic reasoning about victims (Decety, Echols, & Correll, 2010; Fehse, Silveira, Elvers, & Blautzik, 2014), nor was the perpetrator's identifiability manipulated as identified wrongdoers are punished more severely (Kogut, 2011). Additionally, most of the scenarios featured a single victim and not multiple victims, since harm in the former context has been shown to lead to a more robust emotional response than the latter (Konis, Haran, Saporta, & Ayal, 2016). None of the scenarios systematically manipulated information about how reasonable the agent's belief was (Young, Nichols, & Saxe, 2010) or the nature of agent's desires (Cushman, 2008; Laurent, Nuñez, & Schweitzer, 2015). Additionally, all scenarios were formulated in such a way that the agent was in control of his/her own behavior (Martin & Cushman, 2016). The agent was causally responsible for the outcome and no information that would diminish agent's perceived responsibility for the outcome was presented (apart from belief information), e.g. mitigating circumstances (Buckholtz et al., 2008; Yamada et al., 2012) or external constraints on the agent by third-parties (Murray & Lombrozo, 2017; Phillips & Shaw, 2015; Woolfolk, Doris, & Darley, 2006). Moreover, when present, the nature of harmful outcome was described in a plain rather than

graphic language (Treadway et al., 2014). Importantly, all protagonists in scenarios had an obligation towards victims (due to their role in the relational context) and possessed the capacity to foresee and prevent the event (Malle et al., 2014).

Although some of the participants possessed basic understanding of English, we preferred to translate the original material into Italian given that foreign language reduces the relative weight placed on intentions versus outcomes during moral evaluations (Geipel, Hadjichristidis, & Surian, 2016).

Supplementary Text S11: Experimental protocol for the fMRI study (Study 9)

There was no restriction on handedness of participants (8 left-handed, as assessed using self-report) and all participants had normal or corrected-to-normal vision. Although we did not carry out any formal power analysis to settle down on a sample size of 50, we note that it is larger than median sample size in fMRI studies (Poldrack et al., 2017). Rule-out criteria for participation included Italian as a secondary language, presence of a diagnosed psychiatric illness and/or history of psychiatric treatment, history of significant neurological illness or brain injury, and current usage of psychoactive drugs.

Stimuli presentation: Scenarios were presented in the scanner using a visual display presented on an LCD panel and back-projected onto a screen positioned at the front of the magnet bore. Subjects were positioned supine in the scanner so as to be able to view the projector display using a mirror above their eyes. The behavioral data were collected using a Lumina response box (LP-400, Cedrus Corporation, San Pedro, USA). The stimuli were presented using Cogent 2000 (Wellcome Department of Imaging Neuroscience, http://www.fil.ion.ucl.ac.uk/Cogent2000.html) running on MATLAB platform. The text of the stories was presented in a black 21-point Arial font on a white background with a resolution of 800 × 600.

In the same session, participants completed both the moral judgment task and the empathy localizer task. The order in which participants performed moral judgment task and empathy localizer task was counterbalanced across participants.

Moral judgment task: During the rating phase, the location at which the cursor initially appeared on the scale was chosen at random on each trial to make sure that there were no systematic differences across conditions in terms of the required cursor movement, as this could have confounded effects of interest with movement-related activity (especially in ROIs like r-AI (Mutschler et al., 2009)). Additionally, the response buttons were active for as long as the question remained on the screen and participants could move the cursor to one position and could later change it again to a new position. As such, we could not collect any meaningful response time data.

Localizer task: To localize the functional empathy network in participants, we used the task from a prior study (Lamm, Batson, & Decety, 2007). Participants were told that they would be witnessing videos of people experiencing painful auditory stimulation. Note that, as opposed to the instructions provided in the original study, we did not tell participants that this stimulation was part of a medical treatment, as we suspected that this could have led to down-regulation of the empathic response (Lamm et al., 2007).

Questionnaires: Either after or before (randomized across participants) the scanning, participants completed various questionnaires assessing various personality traits (data not reported here).

Supplementary Text S12: Additional details about fMRI preprocessing and data visualization

Given the huge variation in possible preprocessing pipelines and flexibility in methodological choices (Carp, 2012) and differential effect of such variations across softwares (Pauli et al., 2016), we provide extensive details about our choices and rationale for the same here (Poldrack et al., 2017). To report acquisition and preprocessing details, we have followed a prior set of guidelines (Inglis, 2015; Poldrack et al., 2008), while data visualization choices were primarily based on prior recommendations (Allen, Erhardt, & Calhoun, 2012).

Data Acquisition:

High-resolution structural images were acquired as 180 T1-weighted transverse images (0.75 mm slice thickness). Functional images were acquired in interleaved manner using a T2*-weighted echoplanar imaging (EPI) sequence with 33 transverse slices covering the whole brain with the following parameters: slice thickness = 3.2 mm; interslice gap = 0.3 mm; repetition time (TR) = 2000 ms, echo time (TE) = 35 ms; flip angle = 90º, field of view = 230 × 230 mm^2; matrix size = 128 × 128, SENSE factor 2. The slices were oriented at a 30º oblique angle to the AC-PC. This slice prescription was selected for optimization of BOLD signal (by reducing drop-out effects caused by the air-tissue interface) in the orbitofrontal cortex (based on recommendations by Weiskopf, Hutton, Josephs, & Deichmann, 2006).

Preprocessing:

Data were analyzed with SPM12 (www.fil.ion.ucl.ac.uk/spm/software/spm12; Wellcome Department of Imaging Neuroscience, London, UK). First three scans were discarded to avoid T1-equilibration effects. The scans were not slice timing corrected because for relatively short TR (2 seconds or less), it can lead to artifacts (Poldrack, Mumford, & Nichols, 2011, p.42, 48). All functional volumes were realigned in two steps: initially to the first volume and then to the mean realigned image. The estimation of realignment parameters was carried out using a 6-parameter affine (rigid body) transformation such that the cost function comprising of difference in voxel intensities between images was minimized. The average of the motion-corrected images was co-registered to structural MRI scan using a 9-parameter affine transformation such that a suitable between-modality cost function (normalized mutual information) was minimized. The realigned functional images were then normalized to the ICBM-space template (2 mm × 2 mm × 2 mm voxels) for European brains by applying nonlinear deformation field estimated from the best overlay of the atlas image on the individual subject's co-registered structural image. The normalized images were then smoothed by convolving an isotropic Gaussian kernel with full width at half maximum (FWHM) of 10 mm (= $\sqrt{6^2 + 8^2}$, 6 mm at first and 8 mm at second level) in order to- (*i*) boost signal-to-noise ratio to ease the detection of large clusters, (*ii*) overcome imperfections remaining from inter-

subject registration, and (*iii*) validate assumptions of Gaussian random field theory (RFT) applied later to correct for multiple comparisons during statistical analysis (Poldrack et al., 2011, pp.50-52).

Data reporting and visualization:

Combination of the Anatomy Toolbox v2.2c (Eickhoff et al., 2005) and Neuromorphometrics atlas was used for anatomical interpretation. All peaks of activations are reported in MNI-coordinates but no Brodmann Area (BA) labels have been reported as assigning functional activations to cytoarchitectotonically defined BAs can be inaccurate in the absence of probabilistic maps of underlying cytoarchitectotonic variability (Devlin & Poldrack, 2007). All statistical parametric maps are displayed on smoothed, representative scans (average of 305 T1 images, provided in SPM12) and not on a single brain as this can deceive the reader into thinking that anatomical localization is more precise than is actually possible (Ridgway et al., 2008).

Motion and artefact analysis:

To avoid false positive activations owing to head movement, the following data quality checks were employed for each participant and for each task. Data from a participant for a particular task was removed without further analysis if TR-to-TR head movement exceeded 5 mm at any point during the task (none removed).

After this check, the artefact detection analysis was carried out using the Art toolbox (www.nitrc.org/projects/artifact_detect). For each task, outlier scans were identified based on two measures (cf. Koster-Hale, Bedny, & Saxe, 2014): (*a*) if the TR-to-TR composite motion was more than 2mm and/or (*b*) if the scan-to-scan global BOLD signal normalized to *z*-scores deviated from mean more than $z = 3$. Each time-point identified as an outlier was regressed out as a separate nuisance covariate in the first-level design matrix. Note that the motion outliers were identified based on composite motion parameter as this is a more comprehensive measure that outperforms individual motion parameters

(Wilke, 2014). Participant with more than 20% outlier scans were excluded from the analysis (two removed: 21.30% and 25%), but their behavioral data was retained.

Using the Art toolbox, we assessed quality of fMRI data[3] and also ensured that there were no systematic correlations between any of the task-related parameters, realignment parameters, and global BOLD-signal, which can lead to artifactual activation or loss of task-related signal after removing motion-related signal (Poldrack et al., 2011, p.44). Since we regressed out scans with excessive movement and the task regressors were not correlated with BOLD activity, we did not unwarp the realigned images to remove variance associated with susceptibility-by-movement interactions (B0 distortions).

Supplementary Text S13: Additional details for fMRI data analysis

First-level analysis:

Moral judgment task: For this task, 72 regressors of interest (with additional nuisance regressors) from a 6 (text segment: background, foreshadow, mental-state information, consequence, acceptability question, blame question) × 2 (belief: neutral, negative) × 2 (outcome: neutral, negative) × 3 (type of HRF: canonical, time derivative, dispersion derivative) design were defined.

Functional localizer task: In the first-level design matrix for the empathy localizer task, 3 regressors of interest corresponding to the informed basis set (i.e., canonical HRF + its derivatives) convolved with the event of witnessing empathy-eliciting videos and 6 additional regressors for events involving ratings were defined. Each subjects' whole brain *F*-contrast image (experimental videos > baseline) was masked with anatomical atlas labels provided by Neuromorphometrics atlas. Each ROI was defined by peak voxel of cluster containing more than 10 significantly active contiguous voxels ($p < 0.001$, uncorrected, $k > 10$).

[3] We note that, in the interim, better visual techniques have appeared that can help assess quality of fMRI data (Power, 2016), especially to delineate various sources that contribute to noise (Liu, 2016).

For each participant and for each task, the design matrices for a fixed-effects General Linear Model were constructed by convolving a canonical hemodynamic response function or HRF (double gamma, including a positive γ function and a smaller, negative γ function to reflect the BOLD undershoot) with the stimulus function for events (boxcar function) to create regressors of interest. Even a minor misspecification in hemodynamic model can lead to biased estimators and loss of power, possibly inflating the type I error rate (Lindquist, Meng Loh, Atlas, & Wager, 2009). Thus, in order to account for subject-to-subject and voxel-to-voxel variation in evoked BOLD response, the stimulus function was also convolved with partial derivative of canonical HRF with respect to onset latency (which allows for delay in peak response) and dispersion (which relaxes assumption about width of the response) to form the informed basis set (Chen, Taylor, & Cox, 2017; Henson, Rugg, & Friston, 2001).

Note that the inclusion of temporal derivative of HRF also reduces impact of slice timing differences by allowing some degree of timing misspecification, which is crucial for our study since we did not do slice timing correction (Ashby, 2011, pp.47-51). The convolution was performed in a higher resolution time-domain than TR (16 time-bins per TR). As a default, SPM orthogonalizes HRF derivatives on canonical HRF and not on the rest of the design matrix (Pernet, 2014). The orthogonality of other regressors of interest was also visually inspected in design matrices since collinearity between regressors can lead to highly unstable parameter estimates and loss of statistical power (Mumford, Poline, & Poldrack, 2015). High-pass temporal filtering with a cut-off of 128s was used to remove low-frequency drifts and power spectra were visually inspected to ascertain that signals of interest were not being filtered out. Temporal autocorrelations in fMRI time series data were modelled using an autoregressive AR(1) model. Since in the current study neither the ITI was less than 1 second nor was the stimulus exposure duration less than 3 seconds, we were confident that the BOLD-response did not exhibit significant nonlinearities and thus a second-order Volterra series was not modelled in the design matrix for any of the tasks (Ashby, 2011, pp.33-34).

Collinearity diagnostics:

Note that at the first-level each of the six segments of the moral scenario was presented without any jitter[4] from the preceding or succeeding segment and was assigned an independent regressor. This raises the possibility that there was a high or perfect collinearity between regressors which can make the model un-estimable (Henson, 2007; Monti, 2011; Mumford et al., 2015). The collinearity of the model was thus assessed by computing variance inflation factor (VIF) for each of the 78 regressors and average VIF was computed across regressors for each participant. High VIFs can denote that collinearity problem is present. Four participants with high VIFs ($z > 2$, i.e.) were excluded (VIFs = 14.71, 14.61, 18.20, ~ 10^6), but their behavioral data were retained. The remaining participants had average VIF value of 10.97 (SD = 0.75, [min, max] = [9.97, 13.17]). Although these values are above a typical threshold used to detect problematic collinearity (5-10), this rule of thumb has been criticized by noting that the VIFs need to be considered in the context of group sizes (O'brien, 2007).

We additionally note here that in all cases of collinearity, statistical inferences are still valid because although the parameter estimates tend to be variable, so does the variance estimates. As a result, the estimates are unbiased and the rate of false positives is controlled (Kutner, Nachtsheim, Neter, & Li, 2004). Additionally, the impact of collinearity depends on whether this collinearity occurs at the first-level or second-level. As Mumford and colleagues note (2015) –

> 'If the collinearity occurs in the first level, say if two explanatory variables for two trial types are correlated and the interest is in the effect of the first trial type, the individual subject parameter estimates will be highly variable, but when averaged at the group level the estimates that are too large tend to balance out with those that are too small to arrive at an estimate that is closer to the true mean estimate. On the other hand, collinearity between age and gender would occur in the

[4] This was done from the perspective of shortening the total scanning period as we wanted to complete instructions and debriefing, structural scans, resting-state scans, localizer task, and two main experimental tasks within 90 minutes. Introducing jitter between each of the six segments for each of the 36 scenarios would have further elongated the duration of this task (e.g., by about 10 minutes total for jittered intervals averaging 4 seconds) and thus we decided to not include jitter between segments.

group model and in this case the parameter estimates with, say, age may be much larger or smaller than the true effect such that if the magnitude of the effect is of great interest, care should be taken in interpreting it." (p.10)

Indeed, in the current study, we were interested in the regressors at the first level, which were used while computing PSC. These PSC values were used for statistical analysis at the group level instead of parameter estimates for regressors. Considering this discussion and precautions taken, we are thus confident in the statistical validity of our results at the group level.

Second-level analysis:

Moral judgment task: The group-level random effects analyses were conducted for each segment by contrasting the (canonical HRF) beta-weights from each subject's first-level analyses in a single full factorial design generated using a 4 (segment) × 2 (belief) × 2 (outcome) design matrix.

Functional localizer task: The empathy network at group-level was localized by entering beta-weights from all HRF contrasts from first-level in a full factorial design (*F*-contrast).

Heterogeneity of variance between different levels of factors and non-sphericity in the data was accounted for by estimating parameters using Weighted Least Squares (pre-whitening the data using estimated non-sphericity and then applying Ordinary Least Squares; SPM12 Manual, pp.277-78). Not assuming sphericity was especially important for our design since we included informed basis set at first-level that leads to stronger assumption about sphericity (Glaser & Friston, 2004), although only canonical HRF contrasts were retained for the whole-brain analysis for the moral judgment task because of the complexity of design (cf. http://imaging.mrc-cbu.cam.ac.uk/imaging/DealingWithDifference; Chen et al., 2016).

Whole-brain analyses were thresholded at $p < 0.05$, Family-wise Error (FWE) corrected at the voxel-level. Furthermore, we also used ROIs derived from the empathy localizer task for small volume correction to investigate regions which were *a priori* of interest to us. Although the cluster-level inference has greater overall sensitivity over more stringent voxel-level inference, the primary limitation of the former approach is that one can only claim that there is true signal *somewhere* in the large clusters (which can span many anatomical regions) that are found and thus is ill-suited to investigate question about overlapping or distinct activations across conditions and this limitation should be kept in mind while interpreting the results (Woo, Krishnan, & Wager, 2014). Additionally, it has also been shown to inflate the rate of false positives (Eklund, Nichols, & Knutsson, 2016; Han & Glenn, 2017).

Overall grand mean scaling was applied to the data (as recommended, Chen et al., 2016), but no global normalization was used as this procedure has been known to introduce bias in the results (Ashby, 2011, p.97). Also, no implicit threshold masking was applied. Activations lying outside of the brain (due to low variance problem) (Ridgway, Litvak, Flandin, Friston, & Penny, 2012) were weeded out using explicit threshold mask formed by averaging first-level masks for respective task from each participant.

Supplementary Text S14: Localizer task results

Behavioral results

Functional and behavioral data from the localizer task was available for all valid 49 participants, since no participant had to be left out due to excessive motion during this task. The ratings for two questions for the localizer task were provided using a 7-point Likert scale–

(i) *other-oriented* empathic response by gauging intensity of the experienced pain ("How painful was this stimulation for this person?"; -3: *not at all painful* to 3: *extremely painful*),

(ii) *self-oriented* distress via experienced unpleasantness ("How unpleasant was it for you to watch this person suffering?"; -3: *not at all unpleasant* to 3: *extremely unpleasant*).

Ratings revealed that although they recognized that the noxious stimulation was really painful for the protagonist in the video (*M* = 1.393, *SD* = 0.810; *t*(48) = 12.031, *p* < 0.001), this did not elicit self-oriented unpleasantness in proportional manner (*M* = 0.244, *SD* = 1.599; *t*(48) = 1.069, *p* = 0.290), as compared to mid-point of the scale. But note that participants still reported to feel some amount of self-oriented distress.

Correlating the average of ratings provided by participants for the other- and self-oriented empathy questions of the localizer task revealed that none of the ratings were predictive of intent-based moral judgments.

**Rating (*n* = 49)**	**statistic**	**Type of condition**			
		**neutral**	**accidental**	**attempted**	**intentional**
**Other-oriented response**	*r*	0.193	0.242	-0.023	0.175
	p	0.183	0.093	0.873	0.229
**Self-oriented unpleasantnenss**	*r*	0.275	0.084	0.210	0.227
	p	0.055	0.565	0.147	0.117

Although not significant, there was a marginally significant association between pain ratings (for the target in the videos) and severity of moral judgment about accidental harms.

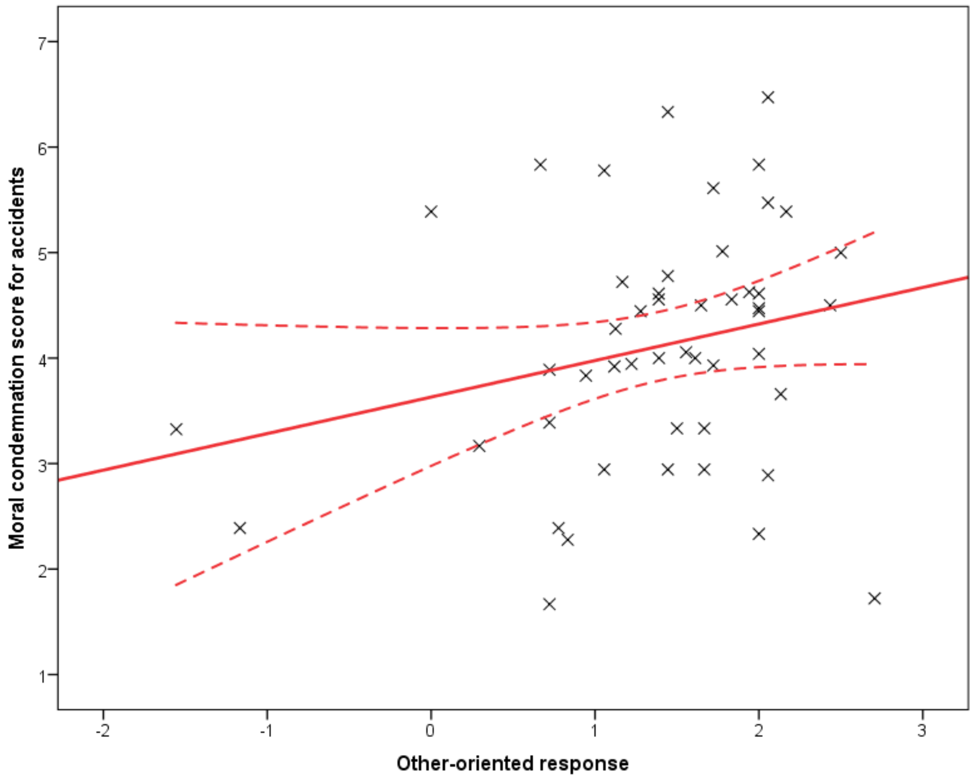

fMRI results

Although there is no consensus on this classification, the key nodes of the empathy network can be classified to belong to two distinct yet interacting components (Eisenberger, 2012) coding for different aspects of the nociceptive episode:

(*i*) *sensory-discriminatory* component codes the painful *stimulus properties* (location, intensity, and duration), which primarily consists (Betti & Aglioti, 2016; Eisenberger, 2012; Keysers, Kaas, & Gazzola, 2010) of the somatosensory cortices (S1, S2) and the bilateral posterior insula (PI);

(*ii*) *affective-motivational* component of the painful *experience* (unpleasantness, negative affect) and consists (Betti & Aglioti, 2016; Lamm, Decety, & Singer, 2011; Zaki & Ochsner, 2012) of

the bilateral anterior insula (AI), the dorsal anterior cingulate cortex (dACC), and the anterior middle cingulate cortex (aMCC).

The average coordinates at second level are tabulated below:

Type of ROI	ROI	Individual ROIs				Whole-brain contrast		
		n	*x*	*y*	*z*	*x*	*y*	*z*
Empathy (> baseline)	dACC	39/46	2	32	23	10	28	26
	l-AI	36/46	-36	13	2	-36	10	0
	r-AI	41/46	37	16	1	40	12	0
	l-PI	29/46	-37	-16	-10	-34	-22	4
	r-PI	26/46	38	-10	4	38	-20	6
	aMCC	42/46	3	5	38	-2	12	42

Note: Average peak voxels for ROIs are in MNI coordinates (in mm). The "Individual ROIs" columns show the average peak voxels for individual subjects' ROIs. The "Whole-brain contrast" columns show the peak voxel in the same regions in the whole-brain random effects group analysis. Results at both subject-level and group-level were masked anatomically by Neuromorphometrics atlas.

Model-free analysis of localizer task data

Since there was no control task that the empathy localizer task was contrasted with, we wanted to assess the robustness of our results using model-free analysis which make fewer assumptions about the data than model-based analysis (like GLM). We performed spatial group independent component analysis (gICA) on preprocessed functional datasets (*n* = 49) of equal length using the GIFT toolbox (v4.0, http://mialab.mrn.org/software/gift/; Calhoun, Liu, & Adalı, 2009) to localize the empathy network.

Summary of rationale behind gICA

The ICA was preferred for localizing functional network (e.g., seed-based correlation analysis) because it provides many advantages over other univariate approaches to functional connectivity in terms of accounting for artefactual influence of confounding signals, such as respiratory, cardiovascular, non-grey

matter, etc. (Cole, Smith, & Beckmann, 2010). The group-ICA method, as implemented in GIFT, involves following steps: initially data from all subjects are spatially normalized and dimensionally reduced by conducting principal component analysis (PCA) at individual subject level. All reduced datasets are then temporally concatenated to form one dataset on which group-ICA is applied. When applied, group-ICA decomposes a two-dimensional data matrix (with columns representing time course of voxels and rows representing different subjects) into two matrices, one corresponding to the time courses of components for the group and the other corresponding to spatial maps for components with component loading for each voxel. Individual-level components are then created via GICA back-reconstruction method based on PCA compression and projection.

Identifying the empathy network component

We closely followed the analysis protocol detailed in a previous study (Hyatt, Calhoun, Pearlson, & Assaf, 2015) and we provide here extensive details about the preprocessing pipeline as data preprocessing can affect gICA results (Vergara, Mayer, Damaraju, Hutchison, & Calhoun, 2016). Subject-specific principal component analysis (PCA) was run to retain 30 principal components (PCs). At the second stage of group data reduction, expectation maximization algorithm was used to retain a final set of 20 PCs (a low model order ICA, i.e.). The Infomax algorithm was then run, repeating it 15 times in ICASSO, to generate a stable set of final 20 components. Finally, subject-specific spatial maps (SMs) and time courses (TCs) were estimated using the GICA back-reconstruction method based on PCA compression and projection. Before looking for the component that represented the empathy network, we first identified a subset of components that were considered BOLD-related brain networks rather than physiological artifacts. To this effect, component viewer facility in GIFT was utilized to compute the frequency spectrum of each component TC and the dynamic range (DR) and fractional amplitude of low-frequency fluctuation (fALFF). Based on frequency-domain analysis (Allen et al., 2011), it has been shown that artefactual components often exhibit both low DR and low fALFF and such components were removed from further analysis after visual inspection (Griffanti et al., 2016). Based on this analysis, 3

components were removed. Out of the 17 biologically meaningful and non-artefactual remaining components, the component corresponding to the empathy network was identified by using spatial correlation feature within GIFT, which identified the component (shown in figure below) with the highest spatial correspondence to the meta-analytic functional map for empathy (Bzdok et al., 2012).

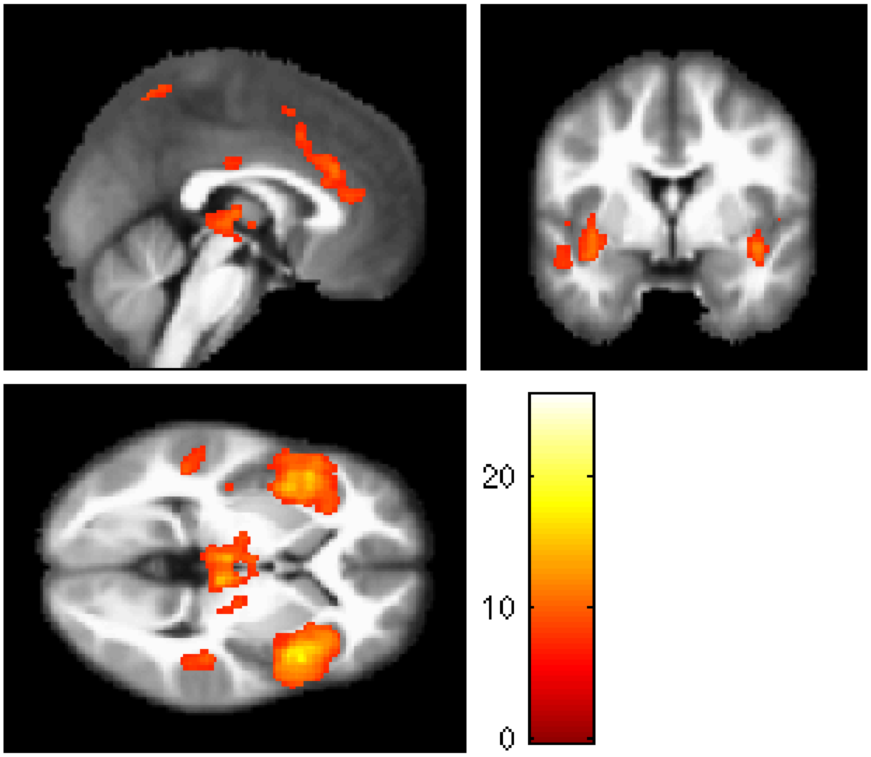

After identifying the component corresponding to the empathy network, the coordinates for the network were derived from a one-sample Student's *t*-test. Statistical significance was assessed using an FWE value of $p < .001$ corrected for the whole-brain volume at the cluster-level ($k > 50$; height threshold: $t = 6.85$). Significant clusters of activation from the component corresponding to the empathy network are provided in the table below.

MNI coordinates			cluster	cluster	peak	peak
x	y	z	p(FWE-corr)	equiv k	p(FWE-corr)	T
-38	18	-6	< 0.00001	3142	< 0.00001	26.09523
-42	18	-20			< 0.00001	15.45017
-38	8	2			< 0.00001	15.37402
34	18	-16	< 0.00001	2999	< 0.00001	19.82562
42	16	-6			< 0.00001	18.06725
44	8	0			< 0.00001	16.97335
6	-22	0	< 0.00001	1157	< 0.00001	14.48983
-4	-20	0			< 0.00001	13.71955
18	-16	2			6.02354E-08	9.667591
-58	-44	32	< 0.00001	312	1.76695E-11	12.46963
-60	-40	24			2.11451E-07	9.291094
-52	-40	28			5.32772E-07	9.016982
46	-24	-6	< 0.00001	523	5.30086E-11	11.88644
60	-30	26			1.89665E-07	9.323516
60	-40	14			9.80747E-07	8.837256
0	30	26	< 0.00001	654	6.71442E-10	11.06384
6	20	28			1.28281E-08	10.13824
0	36	14			1.5717E-07	9.379601
-4	-58	66	< 0.00001	97	9.71824E-09	10.22378
6	-56	66			1.03515E-05	8.151581
-50	-40	2	< 0.00001	238	6.1543E-08	9.661121
-52	-28	-6			1.0258E-06	8.824061
-56	-34	0			6.42715E-06	8.289274
6	-30	24	< 0.00001	136	1.37305E-06	8.738563
6	-42	22			8.00138E-06	8.225923
4	-18	36			1.42003E-05	8.06049

Supplementary Text S15: Additional details for ROI analysis

ROI selection: Although ToM can also be expected to be recruited while thinking about others' emotional experiences, recent work implies that empathizing and mentalizing are two independent cognitive processes that can be dissociated both at the behavioral and neural level (Kanske, Böckler, Trautwein, Lesemann, & Singer, 2016; Tusche, Bockler, Kanske, Trautwein, & Singer, 2016). Additionally, a prior study shows that empathizing with others in physical pain recruits primarily the empathy network, while processing of emotional suffering (without physical pain) recruits regions overlapping with the ToM network (Bruneau, Jacoby, & Saxe, 2015). Since the moral situations in the current studies did not

contain such information, none of the ToM regions were included among ROIs. Additionally, although prior studies also implicate somatosensory cortices (S1, S2) in empathy for pain (Betti & Aglioti, 2016), we did not include them in our ROI list because the task of interest (moral judgment task) did not feature any salient information about specific body parts being subjected to painful stimulation.

ROI analyses: To carry out ROI analysis, we investigated functional specificity (and *not* specialization) (Friston, Rotshtein, Geng, Sterzer, & Henson, 2006) of empathy network for each individual participant using functional localizer task. We note that the ROIs were not tailored to be the same for all participants and were determined on an individual basis for both tasks following individual-subjects functional localization approach (Nieto-Castañón & Fedorenko, 2012).

The data from spherical ROIs with a radius of 8mm was extracted and analyzed using the MarsBar toolbox (v0.44) for SPM (http://marsbar.sourceforge.net/) (Brett, Anton, Valabregue, & Poline, 2002). The GLM model set to time-series of summary statistic (mean signal) from each ROI was similar to that in the whole-brain analysis except that autocorrelations in the time-series were modelled using fmristat AR(2) (http://www.math.mcgill.ca/keith/fmristat/) processes instead of AR(1) processes, since second order autoregressive model is the most parsimonious way to model signal due to aliased physiological artefacts (Lindquist, 2008). Within the ROI, the average percent signal change (PSC) was computed relative to the adjusted mean of the time series. For computing PSC, Marsbar selects a particular event type and finds the design regressors for that event only, removes them, and replaces them with finite impulse response (FIR) basis set for that event only, and discards the FIR model after estimation is done (Matthew Brett, https://sourceforge.net/p/marsbar/mailman/message/35387799/). Since ROI analysis was carried out at the first-level, the smoothing kernel applied to data was 6 mm and not 10 mm.

Quality check was performed by reviewing if any of the PSC values were extreme (> 5%) as these can be indicative of artefacts in the data (Raichle & Mintun, 2006). Data from one additional participant was

removed from only one ROI: r-AI. After this exclusion, the minimum PSC observed was -3.528, while maximum was 3.948.

Supplementary Text S16: Psychophysiological Interaction (PPI) analysis details

Given that our exploratory post hoc comparisons found the difference across acceptability and blame segments for accidental harm condition was significant in r-AI, we carried out functional connectivitiy analysis to see context-dependent changes in r-AI's connectivity with the rest of the brain. The time series were extracted from a seed voxel in the r-AI (at coordinates given by the localizer task) that showed an increase in BOLD signal during blame (versus acceptability) judgments for accidental harm cases at an uncorrected threshold of $p < 0.99$ within 8 mm of this voxel, for each subject individually. Note that such liberal threshold was chosen to ensure all voxels in the ROI are used to compute the connectivity (McLaren, Ries, Xu, & Johnson, 2012). The time series from seed region was summarized by the first eigenvariate across all suprathreshold voxels. The resulting time series were adjusted for effects of no interest by demeaning the eigenvector by all effects not included in that contrast. This BOLD time series was deconvolved to estimate a neuronal time series for this region using the PPI-deconvolution parameter defaults in SPM12 (Gitelman, Penny, Ashburner, & Friston, 2003). The PPI regressor was calculated as the element-by-element product of the ROI neuronal time series and a vector coding for the main effect of task (contrast vectors: *blame* = 1, *acceptability* = -1). This product was then re-convolved with the canonical HRF.

At first-level analysis, we included the PPI as a regressor of interest in a GLM. The task vectors and the extracted time series were modelled as additional regressors, in order to assess the PPI estimates over and above shared functional activation and task-independent correlations in BOLD signal between the seed and other regions (O'Reilly, Woolrich, Behrens, Smith, & Johansen-Berg, 2012). These regressors were convolved with a canonical HRF and high-pass filtered (128 s). Since functional connectivity results have been shown to be severely affected by movement artifacts (Power, Barnes, Snyder, Schlaggar, &

Petersen, 2012), we also included realignment parameters and regressors for outlier in the first-level PPI models. We note that mean centering of the psychological variable is performed by default in SPM12 (r6556), which is important as recent work shows that absence of centering can introduce spurious PPI effects (Di, Reynolds, & Biswal, 2017).

Subject-wise PPI models were run, and contrast images were generated. Regions with positive or negative PPI denote region with greater or lesser context-dependent change in connectivity with the seed region. These subject-wise contrast images were then entered into second-level GLM analyses to generate *t*-maps on which statistical inference was carried out using uncorrected threshold of $p < 0.001$, $k > 10$. We did not choose FWE-correction for this analysis because PPI analyses tend to lack power (O'Reilly et al., 2012) and thus wanted to avoid greater risk of false negatives (Lieberman & Cunningham, 2009). Additionally, we included a large number of nuisance regressors from motion correction, which also led to reduction in degrees of freedom and, thereby, in loss of statistical power.

In passing, we note that we used standardized psychophysiological interaction (sPPI) because we only had two task conditions. Thus, utilizing generalized form of context-dependent PPI (gPPI) (McLaren et al., 2012), which is configured to automatically accommodate more than two task conditions in the same PPI model, did not provide any additional advantage. Recent meta-analysis with PPI studies show that it is a robust and reliable way to assess functional integration (Di, Huang, & Biswal, 2016; Smith, Gseir, Speer, & Delgado, 2016).

The figure corresponding to the PPI effect is depicted below:

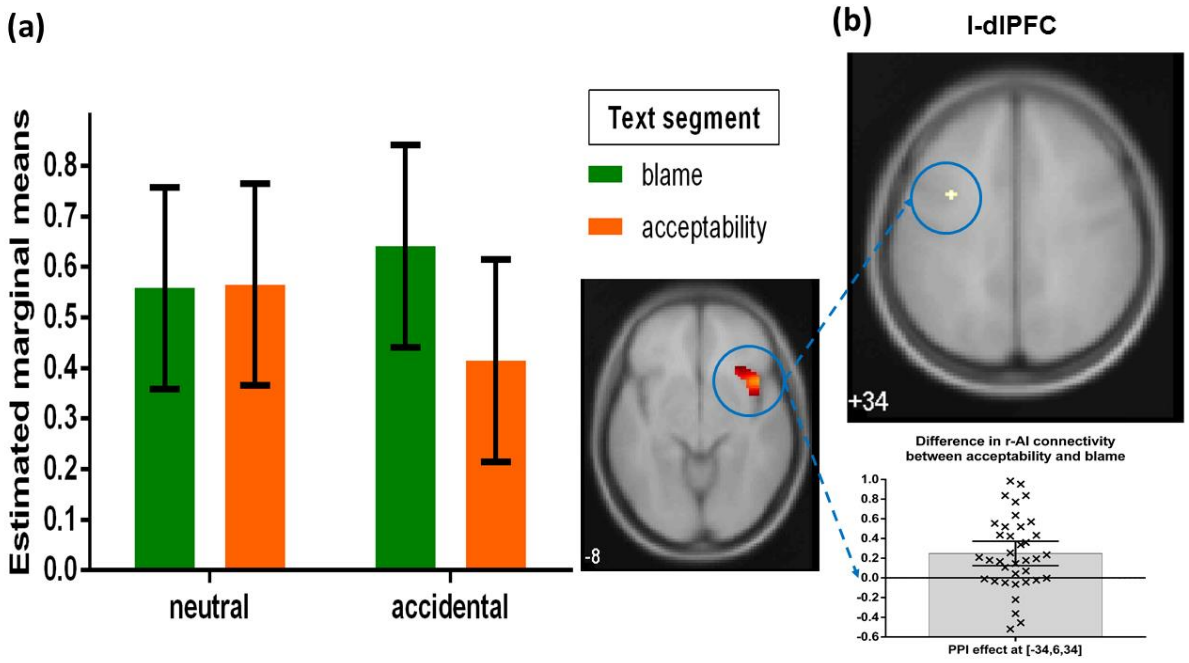

Neural basis of moral luck and connectivity analysis results. (*a*) The estimated PSC in the right anterior insula (r-AI) during the text segments when the participants provided moral judgments. The PSC was higher for blame compared to acceptability judgments only for accidental harm scenarios ($p < 0.05$), but not for no-harm scenarios. Error bars correspond to 95% confidence intervals. (*b*) The only region showing increased functional connectivity (significant positive PPI effect) with r-AI during blame (compared with acceptability) judgments for accidental harm was the left dorsolateral prefrontal cortex (l-dlPFC). Neither l-dlPFC nor any other region showed decreased functional connectivity across decision-contexts (significant negative PPI effect, i.e.). Plot of parameter estimate for PPI effect at the voxel [-34,6,34] are shown in bar graphs. Error bars represent 95% confidence interval. The displayed *z*-coordinates are in MNI-coordinates. Abbreviations - PPI: psychophysiological interaction. PSC: percent signal change.

Supplementary Text S17: Analysis of behavioral data from the fMRI study

Descriptive statistics for the moral judgment task data collected while participants completed this task in the scanner.

condition	question	order	*n*	rating	sd	se	ci
accidental	acceptability	first	208	4.106	2.050	0.142	0.280
		second	220	4.155	2.021	0.136	0.269
	blame	first	220	4.023	2.088	0.141	0.277
		second	210	4.148	2.103	0.145	0.286
attempted	acceptability	first	205	5.185	1.921	0.134	0.265
		second	225	5.298	1.759	0.117	0.231
	blame	first	225	5.187	1.866	0.124	0.245
		second	199	5.060	1.945	0.138	0.272
intentional	acceptability	first	189	6.360	1.086	0.079	0.156
		second	235	6.353	1.219	0.080	0.157
	blame	first	227	6.449	1.194	0.079	0.156
		second	190	6.242	1.257	0.091	0.180
neutral	acceptability	first	196	2.265	1.692	0.121	0.238
		second	226	2.071	1.598	0.106	0.210
	blame	first	230	1.878	1.511	0.100	0.196
		second	202	2.228	1.692	0.119	0.235

The R code used for the LMM was following:

```
lmer(rating ~ belief*outcome*question*order + (1 | id) + (1 |
version), data = data_file)
```

Effect of primary interest shown in bold.

Fixed effect	estimate	se	*df*	*t*	*p*
(Intercept)	2.247	0.139	34.625	16.110	0.000
belief (negative)	2.962	0.169	3361.124	17.541	0.000
outcome (negative)	1.880	0.168	3361.042	11.177	0.000
question (blame)	-0.347	0.164	3365.180	-2.111	0.035
order (second)	-0.154	0.165	3365.850	-0.933	0.351
belief (negative) × outcome (negative)	-0.744	0.240	3364.660	-3.096	0.002
belief (negative) × question (blame)	0.312	0.233	3370.813	1.339	0.181
**outcome (negative) × question (blame)**	**0.231**	**0.233**	**3370.585**	**0.993**	**0.321**
belief (negative) × order (second)	0.230	0.233	3370.862	0.987	0.324
outcome (negative) × order (second)	0.170	0.233	3370.461	0.729	0.466
question (blame) × order (second)	0.463	0.234	3378.446	1.978	0.048
belief (negative) × outcome (negative) × question (blame)	-0.076	0.333	3364.677	-0.227	0.820
belief (negative) × outcome (negative) × order (second)	-0.225	0.332	3365.446	-0.678	0.498
belief (negative) × question (blame) × order (second)	-0.627	0.334	3368.443	-1.880	0.060
outcome (negative) × question (blame) × order (second)	-0.320	0.332	3374.988	-0.963	0.336
belief (negative) × outcome (negative) × question (blame) × order (second)	0.224	0.478	3312.945	0.469	0.639

Supplementary Text S18: Fixed effects for LMM for reading phase segments

Descriptive statistics for the reading phase segments are tabulated below:

segment	condition	PSC	sd	se	ci
background	accidental	-0.260	0.808	0.042	0.083
	attempted	-0.314	0.815	0.042	0.083
	intentional	-0.151	0.785	0.041	0.080
	neutral	-0.299	0.777	0.041	0.080
foreshadow	accidental	-0.043	0.882	0.046	0.090
	attempted	-0.103	0.837	0.043	0.085
	intentional	0.006	0.769	0.040	0.079
	neutral	-0.079	0.817	0.042	0.083
mental-state information	accidental	0.141	0.899	0.046	0.091
	attempted	0.092	0.911	0.047	0.093
	intentional	0.234	0.911	0.047	0.093
	neutral	0.063	0.960	0.050	0.098
consequence	accidental	0.394	0.902	0.047	0.092
	attempted	0.396	0.955	0.049	0.097
	intentional	0.336	0.884	0.046	0.090
	neutral	0.361	0.917	0.048	0.094

The violin plots for the PSC for these segments are shown below:

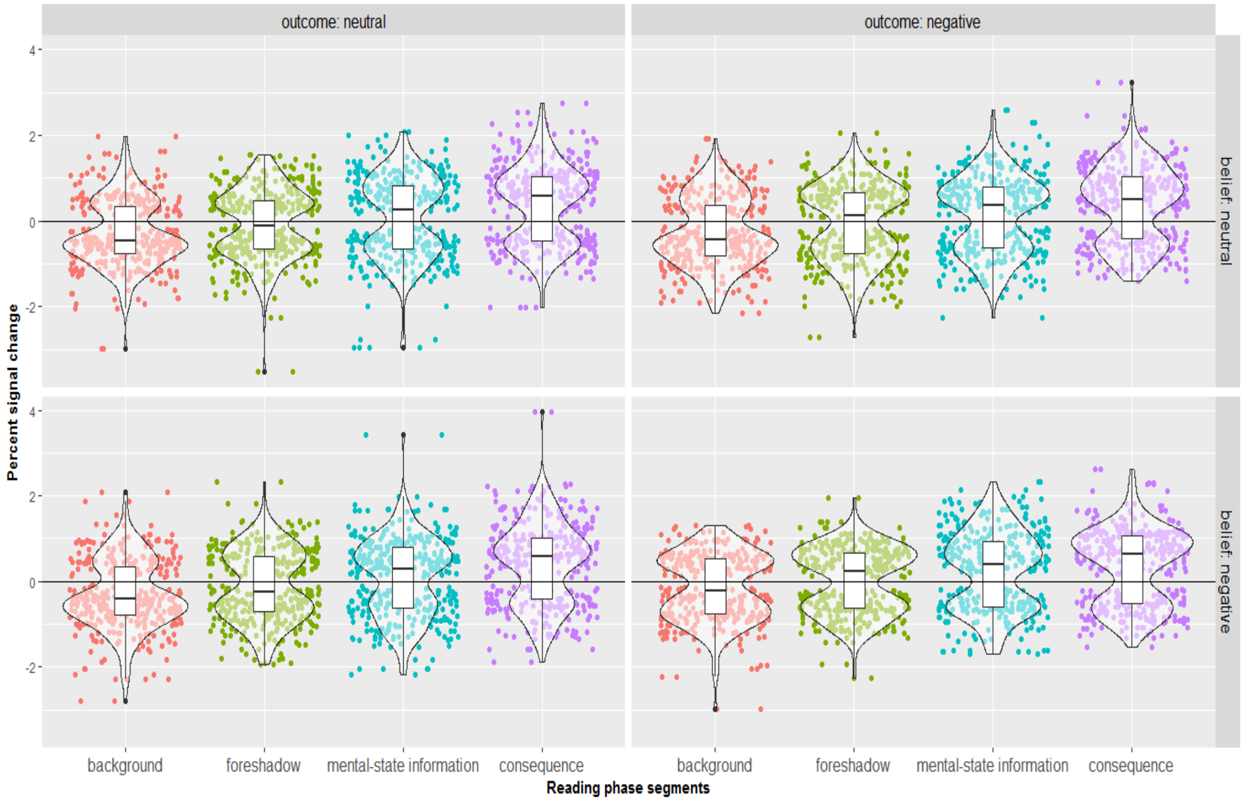

The exact LMM model for the PSC data from the reading phase segments, i.e. *background*, *foreshadow*, *mental-state information*, and *consequence* was the following:

```
lmer(PSC ~ belief*outcome*segment + (1| id) + (1 | ROI), data =
subset(data_file, segment == "background" | segment ==
"foreshadow" | segment == "mental-state information" | segment ==
"consequence"), REML = FALSE)
```

The results from this model are provided below:

**Fixed effects**	**estimate**	**se**	**df**	***t***	***p***
(Intercept)	-0.333	0.090	40.0	-3.717	0.001
belief (negative)	-0.016	0.055	5904.2	-0.289	0.773
outcome (negative)	0.033	0.055	5904.2	0.600	0.549
segment (foreshadow)	0.219	0.055	5904.2	3.957	0.000
segment (mental-state information)	0.358	0.055	5904.2	6.489	0.000
segment (consequence)	0.655	0.055	5904.2	11.855	0.000
belief (negative) × outcome (negative)	0.128	0.078	5904.2	1.642	0.101
belief (negative) × segment (foreshadow)	-0.007	0.078	5904.2	-0.089	0.929
belief (negative) × segment (mental-state information)	0.046	0.078	5904.2	0.590	0.555
belief (negative) × segment (consequence)	0.053	0.078	5904.2	0.682	0.495
outcome (negative) × segment (foreshadow)	-0.001	0.078	5904.2	-0.009	0.993
outcome (negative) × segment (mental-state information)	0.046	0.078	5904.2	0.591	0.554
outcome (negative) × segment (consequence)	0.002	0.078	5904.2	0.025	0.980
belief (negative) × outcome (negative) × segment (foreshadow)	-0.053	0.110	5904.2	-0.484	0.629
belief (negative) × outcome (negative) × segment (mental-state information)	-0.065	0.110	5904.2	-0.593	0.553
belief (negative) × outcome (negative) × segment (consequence)	-0.223	0.110	5904.2	-2.027	0.043

Supplementary Text S19: Mixed-effects regression assessing brain-behavior relationship

Consequence segment

The mixed-effects regression models used was the following-

```
lmer(avg_rating ~ PSC*belief*outcome + (1 + PSC | ID) + (1 + PSC | ROI), data = subset(intent_empathy_PSC, segment == "consequence"), REML = FALSE)
```

The mixed-effects regression showed that the greater PSC in the empathy network while reading consequences of the actor's actions was associated with more condemnation for negative outcomes, but not neutral outcomes.

Fixed effect	estimate	se	df	*t*	*p*
(Intercept)	2.046	0.083	71.876	24.533	0.000
PSC	-0.069	0.060	157.616	-1.153	0.251
belief (negative)	3.042	0.069	1452.013	44.314	0.000
outcome (negative)	2.214	0.069	1450.592	32.109	0.000
PSC × belief (negative)	-0.018	0.069	1463.278	-0.258	0.796
PSC × outcome (negative)	0.148	0.071	1457.995	2.080	0.038
belief (negative) × outcome (negative)	-0.936	0.097	1446.244	-9.628	0.000
PSC × belief (negative) × outcome (negative)	-0.089	0.100	1462.696	-0.892	0.373

Judgment phase

The mixed-effects regression models used were the following-

```
lmer(avg_rating ~ PSC*belief*outcome + (1 + PSC | ID) + (1 + PSC | ROI), data = subset(intent_empathy_PSC, segment == "acceptability" | segment == "blame"), REML = FALSE)
```

The mixed-effects regression showed that the greater the PSC in the empathy network during the judgment phase the greater was the condemnation for negative outcomes but not neutral outcomes.

Fixed effect	estimate	se	df	*t*	*p*
(Intercept)	2.071	0.083	52.430	25.022	0.000
PSC	-0.088	0.063	67.924	-1.398	0.167
belief (negative)	3.024	0.049	2949.892	61.230	0.000
outcome (negative)	2.208	0.049	2943.358	45.432	0.000
PSC × belief (negative)	0.040	0.062	2952.435	0.637	0.524
PSC × outcome (negative)	0.196	0.063	2947.937	3.119	0.002
belief (negative) × outcome (negative)	-0.917	0.070	2944.597	-13.092	0.000
PSC × belief (negative) × outcome (negative)	-0.162	0.089	2954.663	-1.824	0.068

Supplementary Text S20: Fixed effects for LMM for judgment phase segments

Descriptive statistics for the reading phase segments are tabulated below:

segment	condition	PSC	sd	se	ci
acceptability	accidental	0.197	0.728	0.038	0.074
	attempted	0.331	0.797	0.041	0.081
	intentional	0.433	0.700	0.036	0.071
	neutral	0.322	0.688	0.036	0.070
blame	accidental	0.372	0.733	0.038	0.075
	attempted	0.380	0.729	0.038	0.074
	intentional	0.371	0.665	0.034	0.068
	neutral	0.345	0.706	0.037	0.072

The violin plots for these segments are shown below:

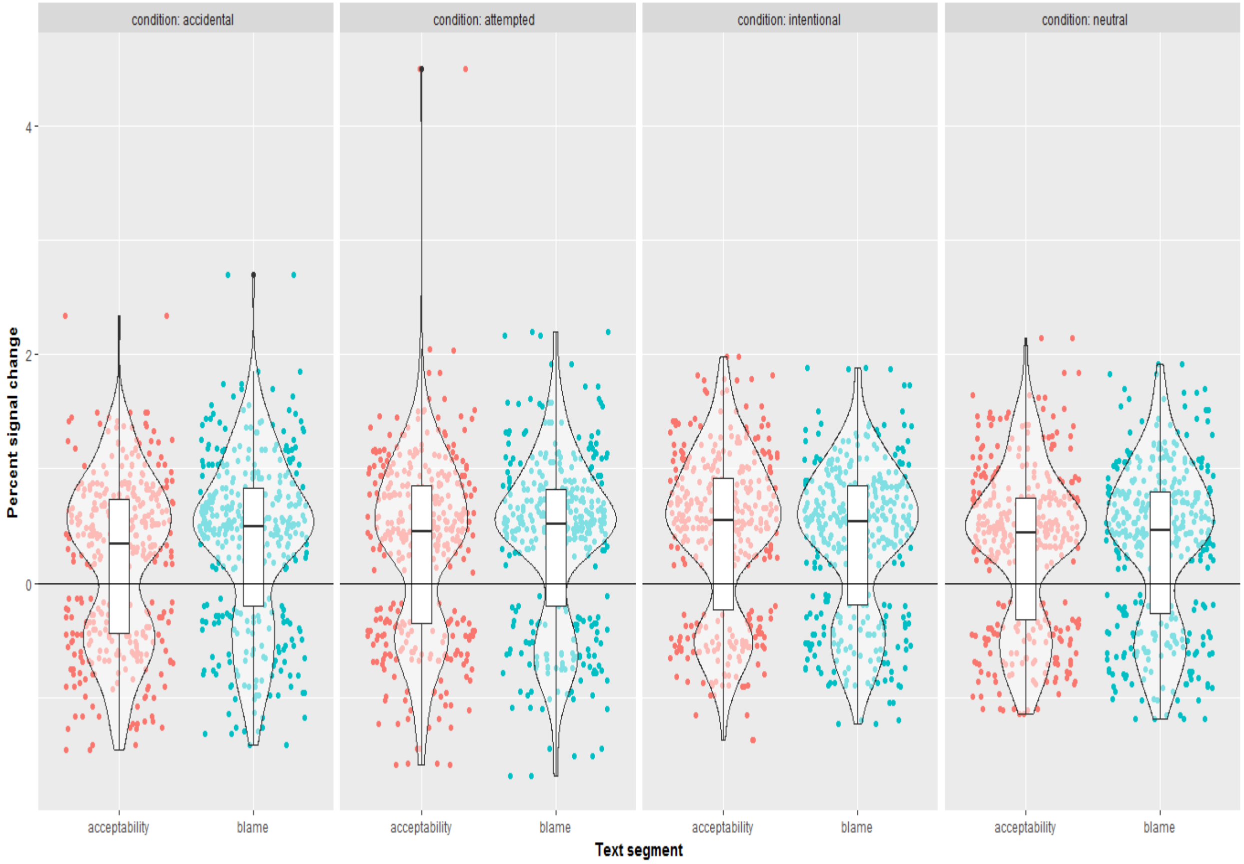

The exact LMM model for the PSC data from the judgment phase segments, i.e. *acceptability* and *blame* was the following:

```
lmer(PSC ~ belief*outcome*segment + (1| ID) + (1 | ROI), data =
subset(data_file, segment == "acceptability" | segment ==
"blame"), REML = FALSE)
```

The results from this model are provided below:

Fixed effects	estimate	se	*df*	*t*	*p*
(Intercept)	0.277	0.102	18.4	2.730	0.014
belief (negative)	0.009	0.042	2939.7	0.216	0.829
outcome (negative)	-0.126	0.042	2939.7	-2.989	0.003
segment (blame)	0.023	0.042	2939.7	0.539	0.590
belief (negative) × outcome (negative)	0.227	0.059	2939.7	3.824	0.000
belief (negative) × segment (blame)	0.026	0.059	2939.7	0.439	0.661
outcome (negative) × segment (blame)	0.152	0.059	2939.7	2.553	0.011
belief (negative) × outcome (negative) × segment (blame)	-0.262	0.084	2939.7	-3.119	0.002

Moral luck effect observed in PSC data but not behavioral data:

This result begs the question as to why the moral luck effect was observed in the PSC data, but not in the behavioral data. Here we provide possible explanations that are neither exhaustive nor are they mutually exclusive:

1. One explanation can be that there was less amount of variation in the behavioral data (coefficient of variation for accidental condition: blame = 29.1%, acceptability = 29.3%) as compared to the PSC data (coefficient of variation for accidental condition: blame = 54%, acceptability = 58.9%) due to restricted range of ratings that could be recorded in the scanner. A previous study (Patil et al., 2017) could behaviorally replicate this effect in within-subjects design with a visual analog scale (VAS) with continuous ratings on [0-20] scale.

2. Another explanation stems from interaction of self-consistency effects and type of scale used. Since participants provided acceptability and blame judgments for each item, they could have been more likely to be consistent with their choices on a Likert scale. Prior study that featured a similar design sidestepped this issue by using VAS with no ticks, which made it difficult for participants to keep track of their choices. Consequently, the outcome-by-judgment interaction was observed (Patil et al., 2017). In the current fMRI study, due to technical issues with the response pad we could not implement VAS in-

scanner and had to resort to using Likert scale where it was easier for participants to remember their choices on consecutive trials and, therefore, being consistent in their responses.

Supplementary Text S21: Whole-brain results for moral judgment task

The whole-brain analyses didn't reveal any effects of interest to us for any of the contrasts (p(FWE-corrected) > 0.05).

To investigate if the moral luck effect observed in the PSC data was also observed at the whole-brain level, we focused on the following analogous contrast: [(accidental: blame > acceptability) > (neutral: blame > acceptability)]. A small volume correction (SVC) with a sphere of 10 mm radius was used according to the ROI coordinates provided by the whole-brain level analysis of the empathy localizer task (r-AI: [40, 12, 0]; l-AI: [-36, 10, 0]; r-PI: [38, -20, 6]; l-PI: [-34, -22, 4]; ACC: [10, 28, 26], MCC: [-2, 12, 42]). Note that this analysis utilized the full sample (n = 42), as opposed to more restricted sample for the ROI analysis.

This revealed a significant cluster only in r-AI (x = 42, y = 14, z = 8; t = 3.20, p(FWE-corrected) = 0.021, k = 3), but in none of the other ROIs. We acknowledge here that adjusting for number of ROIs would lead to a threshold of significance of 0.008, but we refrain from adopting such stringent threshold given the higher risk of Type II error associated with this approach (Cunningham & Koscik, 2017; Lieberman & Cunningham, 2009). It is important to underscore here that the coordinates at which SVC was applied were derived from an independent task and not the task of interest (Poldrack, 2007). Thus, out of all the ROIs in which a significant moral luck effect was observed, only r-AI exhibited this effect at the whole-brain level.

As strongly recommended, we did not carry out whole-brain across-subject correlations between contrast estimates and moral judgments since the sample size in the current study was small to reliably carry out such analysis (Dubois & Adolphs, 2016; Vul & Pashler, 2017).

Supplementary Text S22: Supplementary Discussion

- *Activity in the empathy network reflects empathy for victim or perpetrator?*

In principle, the empathy network could also be invoked to represent the mental states of the perpetrator. Crucially, however, this network is specifically implicated in the representation of suffering, pain and harm. Within the context of our stimuli, these mental states are likely to be imputed exclusively to the victim, and not to the perpetrator. While our participants likely attended to the malicious mental states of the perpetrator (i.e., her intent to harm), past research suggests that the representation of such mental states are represented in a distinct network of brain regions sometimes called the Theory of Mind network, or mentalizing network (Young, Cushman, Hauser, & Saxe, 2007; Young & Saxe, 2008).

To belabor this point further, we are *not* claiming that the empathy network is implicated exclusively for the victim. Instead, we are arguing that the empathic response will be engaged any time there is information about pain or harm, be it in the victim or the perpetrator. The oft-observed empathic sensitivity's focus on the victim over the agent stems from the fact that, in the absence of explicit instruction, people attend more to victims than agents (Decety, Michalska, & Kinzler, 2012). This in turn may be result of the fact that- (*i*) information about pain and suffering (in the victim) is not only prioritized over processing of mental states (of the agent) but can even actively disrupt it given its greater salience (Kanske et al., 2016); (*ii*) in most realistic contexts, and also in hypothetical situations in the current study, information about affective states is available only for the victim and not for the perpetrator. For example, if Amy accidentally burns Steve's hand, we will recoil from empathizing with Steve (*how* the victim feels: empathy), while try to figure out Amy's motivations (*why* the perpetrator did what she did: Theory of Mind).

There is some prior work which shows that, if prompted to focus on the perpetrator, greater activity in the insular cortex predicts reduced punishment (Yamada et al., 2012), but it is difficult to take this to mean that participants reduced punishments for the perpetrators because they *empathized* to a greater degree

with the perpetrators since no information about their affective states was provided. It is more likely that this reduction in condemnation stems from sympathy, a general concern for others' wellbeing that is distinct from empathy (Bloom, 2016, 2017; Jordan et al., 2016) and which also elicits activity in the insular cortex (Immordino-Yang, McColl, Damasio, & Damasio, 2009; Yang, Bossmann, Schiffhauer, Jordan, & Immordino-Yang, 2012). The same conclusion can also be reached based on recent work showing that asking participants to empathize with the perpetrator (focusing on his feeling and emotions, i.e.) who acted unethically (intentionally or otherwise) does not lead to reduced or increased moral condemnation (Lucas, Galinsky, & Murnighan, 2016). Instead perspective-taking (focusing on thoughts and beliefs) led to increased moral condemnation for intentional harms, while reduced condemnation for accidental harms (Lucas et al., 2016).

Additionally, if we were to assume that the activity in the empathy network reflects empathy for the perpetrator, it is difficult to explain the positive relationship observed between activity and severity of moral condemnation for acts with harmful outcomes. That is, it is difficult to explain why sharing the perpetrator's pain to a greater degree would increase severity of moral judgment for the perpetrator. A parsimonious explanation would be that the activity reflects empathy for the victim, which would also explain this positive relationship as greater empathizing with victim's pain can indeed motivate blame for the perpetrator who produced harmful outcome.

- *Participants can choose to not empathize with the victim?*

The participant can recognize that the victim is suffering and yet not empathize with them. Indeed, such top-down influence is often observed, although principally in contexts where the participant is motivated to do. For example, while empathizing with outgroup members in competitive contexts (Hein, Silani, Preuschoff, Batson, & Singer, 2010), victims who bring bad outcomes on themselves by being careless (Fehse et al., 2014), victims who are perceived to possess immoral character (Cui, Ma, & Luo, 2016; Singer et al., 2006), when they perceive any expectation to engage in costly helping behavior (Cameron &

Payne, 2011), etc. Since no such information about the victim was given in our scenarios, we think it is unlikely that there will be such down-regulation in the current study.

Recent work also shows that people can actively choose to avoid entering into empathy-eliciting situations when alternative courses of actions are possible, given the effort involved in empathizing with someone (Cameron, Hutcherson, Ferguson, Scheffer, & Inzlicht, 2017; Cameron, Inzlicht, & Cunningham, 2017), and can even be incentivized with monetary rewards to empathize more (Hess, Blaison, & Dandeneau, 2017). But, in our current paradigm, people were not given either the option of avoiding empathizing with the victim by choosing some alternative course of action. Thus, we maintain that it is possible but unlikely that participants chose not to empathize with the victim.

- *What aspect of harm is the empathy network representing?*

The way we have defined the empathy network here may give the impression that we are subscribing to the strong claim that the insular cortex (AI and PI) and cingulate cortices (dACC and aMCC) are *specific* to empathy or pain perception (Lieberman & Eisenberger, 2015; Segerdahl, Mezue, Okell, Farrar, & Tracey, 2015). But this is far from being the case: these regions are involved in the processing of a variety of non-nociceptive, multimodal sensory inputs (Liberati et al., 2016; Wager et al., 2016) and undergird a host of other cognitive and affective functions (Adolfi et al., 2017; Corradi-Dell'Acqua, Tusche, Vuilleumier, & Singer, 2016) and are found to be active even in the absence of subjective sensation of pain (Salomons, Iannetti, Liang, & Wood, 2016). Despite this, we are reverse inferring the activity observed in this network here to be an index of harm-relevant empathic processing given that this activity was extracted from regions which showed increased activity while empathizing with others' in pain in an independent localizer task. The important question then is what aspect of harmful experience is the empathy network representing? We speculate on this question here.

The shared representations account (Lamm, Bukowski, & Silani, 2016; Zaki, Wager, Singer, Keysers, & Gazzola, 2016) posits that empathizing with others in pain leads to sharing of primarily affective

(unpleasantness and negative affect) and to a lesser degree sensory component of the painful experience. Thus, this account posits that the activity in the empathy network while representing harm to victims can be taken to mean vicarious sharing of affective states in the observer or moral judges (shared negative emotions and somatic representations). Accordingly, multivariate brain patterns that distinguish painful from neutral stimulations are shared between the self and the other (Corradi-Dell'Acqua, Hofstetter, & Vuilleumier, 2011) (favoring shared representations account), but the same is not observed when such multivariate patterns classify different intensities of painful experience (Krishnan et al., 2016) (contradicting shared representations account). Part of these inconsistencies lies in acknowledging the fact that no study yet has comprehensively considered the constellation of phenomena that comprise painful experience (unpleasantness, somatic representations, arousal, attention, etc.). A recent study that tries to account for such diverse functions of the insular cortex shows that, more broadly, these regions process different types of aversive events (painful, disgusting, unfair) through a common modality-independent code, reflecting the shared unpleasantness of such experiences (Corradi-Dell'Acqua et al., 2016). Thus, it is possible that the activity in this network while encoding and integrating information about harmful outcomes represents modality-independent unpleasantness and negative affect[5] that informs the judge's moral judgments (cf. Cheng, Ottati, & Price, 2013).

From another perspective, the empathy network and the pain matrix have been reconceptualized more broadly to be a part of the salience network (Legrain, Iannetti, Plaghki, & Mouraux, 2011; Menon & Uddin, 2010), which is involved in detecting and orienting attention towards sensory stimuli that are crucial for homeostatic balance and pain represents one such salient aspect of the internal and external environment (Uddin, 2014). Converging evidence demonstrates that activity in the r-AI correlates with

---

[5] Note that we are not arguing that the empathy network tracks the *arousal* in response to representation of others' pain, but only the unpleasantness itself. The emotional arousal stemming from pain perception is likely encoded in the amygdala (Bas-Hoogendam, van Steenbergen, Kreuk, van der Wee, & Westenberg, 2017; Buckholtz & Marois, 2012; Krueger & Hoffman, 2016; Ngo et al., 2015; Shenhav & Greene, 2014; Treadway et al., 2014; Yu, Li, & Zhou, 2015).

subjective salience across diverse task domains (Uddin, 2014). Additionally, the r-AI forms the central node of the salience network and coordinates activity of other large-scale neurocognitive networks by causally influencing activity in central hubs of such networks (Uddin, 2014). For example, detection of salient event like experience of pain leads to r-AI-induced changes in the activity of the dlPFC, a central hub in the central executive network that orchestrates externally oriented cognition, and allocates attentional resources to attend to salient event (Menon & Uddin, 2010). Thus, from the empathy network as salience processing network perspective, the selectively greater activation in the network during blame versus acceptability judgments for accidental harm cases could be interpreted to mean that subjective salience of information about harmfulness of the outcome is greater when one needs to decide on how much blame to attribute to the agent as compared to when agent's behavior needs to be evaluated on right-wrong dimension. Additionally, the correlation patterns can be interpreted to mean that the degree to which the harmful outcomes are perceived to be subjectively salient determines how severely the agents involved in producing harmful outcomes are going to be morally condemned.

References

Adolfi, F., Couto, B., Richter, F., Decety, J., Lopez, J., Sigman, M., … Ibáñez, A. (2017). Convergence of interoception, emotion, and social cognition: A twofold fMRI meta-analysis and lesion approach. *Cortex*, *88*, 124–142. http://doi.org/10.1016/j.cortex.2016.12.019

Allen, E. A., Erhardt, E. B., & Calhoun, V. D. (2012). Data visualization in the neurosciences: overcoming the curse of dimensionality. *Neuron*, *74*(4), 603–8. http://doi.org/10.1016/j.neuron.2012.05.001

Allen, E. A., Erhardt, E. B., Damaraju, E., Gruner, W., Segall, J. M., Silva, R. F., … Calhoun, V. D. (2011). A baseline for the multivariate comparison of resting-state networks. *Frontiers in Systems Neuroscience*, *5*, 2. http://doi.org/10.3389/fnsys.2011.00002

Ashby, F. G. (2011). *Statistical analysis of fMRI data* (1st ed.). Cambridge, Massachusetts: MIT Press.

Barr, D. J., Levy, R., Scheepers, C., & Tily, H. J. (2013). Random effects structure for confirmatory hypothesis testing: Keep it maximal. *Journal of Memory and Language*, *68*(3), 255–278. http://doi.org/10.1016/j.jml.2012.11.001

Bas-Hoogendam, J. M., van Steenbergen, H., Kreuk, T., van der Wee, N. J. A., & Westenberg, P. M. (2017). How embarrassing! The behavioral and neural correlates of processing social norm violations. *PLoS ONE*, *12*(4), e0176326. http://doi.org/10.1371/journal.pone.0176326

Batson, C. D. (2009). These things called empathy: eight related but distinct phenomena. In J. Decety & W. Ickes (Eds.), *The Social Neuroscience of Empathy* (pp. 3–16). The MIT Press.

http://doi.org/10.7551/mitpress/9780262012973.001.0001

Berg-Cross, L. (1975). Intentionality, degree of damage, and moral judgments. *Child Development*, *46*(4), 970–974. http://doi.org/10.2307/1128406

Betti, V., & Aglioti, S. M. (2016). Dynamic construction of the neural networks underpinning empathy for pain. *Neuroscience and Biobehavioral Reviews*, *63*, 191–206. http://doi.org/10.1016/j.neubiorev.2016.02.009

Bloom, P. (2016). *Against Empathy: The Case for Rational Compassion* (1st ed.). Ecco.

Bloom, P. (2017). Empathy and Its Discontents. *Trends in Cognitive Sciences*, *21*(1), 24–31. http://doi.org/10.1016/j.tics.2016.11.004

Brett, M., Anton, J., Valabregue, R., & Poline, J. (2002). Region of interest analysis using the MarsBar toolbox for SPM 99. *Neuroimage*, *16*(2), S497.

Brewer, R., Marsh, A., Catmur, C., Cardinale, E. M., Stoycos, S., Cook, R., & Bird, G. (2015). The impact of autism spectrum disorder and alexithymia on judgments of moral acceptability. *Journal of Abnormal Psychology*, *124*(3), 589–95. http://doi.org/10.1037/abn0000076

Bruneau, E., Jacoby, N., & Saxe, R. (2015). Empathic control through coordinated interaction of amygdala, theory of mind and extended pain matrix brain regions. *NeuroImage*, *114*, 105–119. http://doi.org/10.1016/j.neuroimage.2015.04.034

Buckholtz, J. W., Asplund, C. L., Dux, P. E., Zald, D. H., Gore, J. C., Jones, O. D., & Marois, R. (2008). The Neural Correlates of Third-Party Punishment. *Neuron*, *60*(5), 930–940. http://doi.org/10.1016/j.neuron.2008.10.016

Buckholtz, J. W., & Marois, R. (2012). The roots of modern justice: cognitive and neural foundations of social norms and their enforcement. *Nature Neuroscience*, *15*(5), 655–661. http://doi.org/10.1038/nn.3087

Bzdok, D., Schilbach, L., Vogeley, K., Schneider, K., Laird, A. R., Langner, R., & Eickhoff, S. B. (2012). Parsing the neural correlates of moral cognition: ALE meta-analysis on morality, theory of mind, and empathy. *Brain Structure & Function*, *217*(4), 783–96. http://doi.org/10.1007/s00429-012-0380-y

Calhoun, V. D., Liu, J., & Adalı, T. (2009). A review of group ICA for fMRI data and ICA for joint inference of imaging, genetic, and ERP data. *NeuroImage*, *45*(1), S163–S172. http://doi.org/10.1016/j.neuroimage.2008.10.057

Cameron, C. D., Hutcherson, C., Ferguson, A., Scheffer, J., & Inzlicht, M. (2017). Empathy is a Choice: People are Empathy Misers Because They are Cognitive Misers. *Journal of Experimental Psychology: General*. Retrieved from https://ssrn.com/abstract=2887903

Cameron, C. D., Inzlicht, M., & Cunningham, W. A. (2017). Deconstructing empathy: A motivational framework for the apparent limits of empathy. Retrieved from https://osf.io/preprints/psyarxiv/d99bp

Cameron, C. D., & Payne, B. K. (2011). Escaping affect: How motivated emotion regulation creates insensitivity to mass suffering. *Journal of Personality and Social Psychology*, *100*(1), 1–15. http://doi.org/10.1037/a0021643

Carp, J. (2012). On the plurality of (methodological) worlds: Estimating the analytic flexibility of fmri experiments. *Frontiers in Neuroscience*, *6*, 149. http://doi.org/10.3389/fnins.2012.00149

Chen, G., Taylor, P. A., & Cox, R. W. (2017). Is the Statistic Value All We Should Care about in

Neuroimaging? *NeuroImage*, *147*, 952–959. http://doi.org/10.1016/j.neuroimage.2016.09.066

Cheng, J. S., Ottati, V. C., & Price, E. D. (2013). The arousal model of moral condemnation. *Journal of Experimental Social Psychology*, *49*(6), 1012–1018. http://doi.org/10.1016/j.jesp.2013.06.006

Cole, D. M., Smith, S. M., & Beckmann, C. F. (2010). Advances and pitfalls in the analysis and interpretation of resting-state FMRI data. *Frontiers in Systems Neuroscience*, *4*, 8. http://doi.org/10.3389/fnsys.2010.00008

Corradi-Dell'Acqua, C., Hofstetter, C., & Vuilleumier, P. (2011). Felt and Seen Pain Evoke the Same Local Patterns of Cortical Activity in Insular and Cingulate Cortex. *The Journal of Neuroscience* , *31*(49), 17996–18006. http://doi.org/10.1523/JNEUROSCI.2686-11.2011

Corradi-Dell'Acqua, C., Tusche, A., Vuilleumier, P., & Singer, T. (2016). Cross-modal representations of first-hand and vicarious pain, disgust and fairness in insular and cingulate cortex. *Nature Communications*, *7*, 10904. http://doi.org/10.1038/ncomms10904

Cui, F., Ma, N., & Luo, Y.-J. (2016). Moral judgment modulates neural responses to the perception of other's pain: an ERP study. *Scientific Reports*, *6*, 20851. http://doi.org/10.1038/srep20851

Cunningham, W. A., & Koscik, T. R. (2017). Balancing Type I and Type II error concerns in fMRI through compartmentalized analysis. *Cognitive Neuroscience*. http://doi.org/10.1080/17588928.2017.1299122

Cushman, F. (2008). Crime and punishment: distinguishing the roles of causal and intentional analyses in moral judgment. *Cognition*, *108*(2), 353–80. http://doi.org/10.1016/j.cognition.2008.03.006

Cushman, F. (2015). Deconstructing intent to reconstruct morality. *Current Opinion in Psychology*, *6*, 97–103. http://doi.org/10.1016/j.copsyc.2015.06.003

Cushman, F., Dreber, A., Wang, Y., & Costa, J. (2009). Accidental outcomes guide punishment in a "trembling hand" game. *PLoS ONE*, *4*(8), e6699. http://doi.org/10.1371/journal.pone.0006699

Davis, M. H. (1983). Measuring individual differences in empathy: Evidence for a multidimensional approach. *Journal of Personality and Social Psychology*, *44*(1), 113–126. http://doi.org/10.1037/0022-3514.44.1.113

de Vignemont, F., & Singer, T. (2006). The empathic brain: how, when and why? *Trends in Cognitive Sciences*, *10*(10), 435–41. http://doi.org/10.1016/j.tics.2006.08.008

Decety, J., & Cowell, J. M. (2014a). Friends or Foes: Is Empathy Necessary for Moral Behavior? *Perspectives on Psychological Science*, *9*(5), 525–537. http://doi.org/10.1177/1745691614545130

Decety, J., & Cowell, J. M. (2014b). The complex relation between morality and empathy. *Trends in Cognitive Sciences*, *18*(7), 337–9. http://doi.org/10.1016/j.tics.2014.04.008

Decety, J., Echols, S., & Correll, J. (2010). The blame game: the effect of responsibility and social stigma on empathy for pain. *Journal of Cognitive Neuroscience*, *22*(5), 985–997. http://doi.org/10.1162/jocn.2009.21266

Decety, J., Michalska, K. J., & Kinzler, K. D. (2012). The contribution of emotion and cognition to moral sensitivity: A neurodevelopmental study. *Cerebral Cortex*, *22*(1), 209–20. http://doi.org/10.1093/cercor/bhr111

Devlin, J., & Poldrack, R. (2007). In praise of tedious anatomy. *NeuroImage*, *37*(4), 1033-41–8. http://doi.org/10.1016/j.neuroimage.2006.09.055

Di, X., Huang, J., & Biswal, B. B. (2016). Task modulated brain connectivity of the amygdala: a meta-

analysis of psychophysiological interactions. *Brain Structure & Function*, *222*(1), 619–634. http://doi.org/10.1007/s00429-016-1239-4

Di, X., Reynolds, R. C., & Biswal, B. B. (2017). Imperfect (de)convolution may introduce spurious psychophysiological interactions and how to avoid it. *Human Brain Mapping*, *38*(4), 1723–1740. http://doi.org/10.1002/hbm.23413

Dubois, J., & Adolphs, R. (2016). Building a Science of Individual Differences from fMRI. *Trends in Cognitive Sciences*, *20*(6), 425–443. http://doi.org/10.1016/j.tics.2016.03.014

Eickhoff, S. B., Stephan, K. E., Mohlberg, H., Grefkes, C., Fink, G. R., Amunts, K., & Zilles, K. (2005). A new SPM toolbox for combining probabilistic cytoarchitectonic maps and functional imaging data. *NeuroImage*, *25*(4), 1325–1335. http://doi.org/10.1016/j.neuroimage.2004.12.034

Eisenberger, N. I. (2012). The pain of social disconnection: examining the shared neural underpinnings of physical and social pain. *Nature Reviews Neuroscience*, *13*(6), 421–34. http://doi.org/10.1038/nrn3231

Eklund, A., Nichols, T. E., & Knutsson, H. (2016). Cluster failure: Why fMRI inferences for spatial extent have inflated false-positive rates. *Proceedings of the National Academy of Sciences*, *113*(28), 7900–7905. http://doi.org/10.1073/pnas.1602413113

Fehse, K., Silveira, S., Elvers, K., & Blautzik, J. (2014). Compassion, guilt and innocence: An fMRI study of responses to victims who are responsible for their fate. *Social Neuroscience*, *10*(3), 243–252. http://doi.org/10.1080/17470919.2014.980587

Friston, K., Rotshtein, P., Geng, J. J., Sterzer, P., & Henson, R. (2006). A critique of functional localisers. *NeuroImage*, *30*(4), 1077–87. http://doi.org/10.1016/j.neuroimage.2005.08.012

Geipel, J., Hadjichristidis, C., & Surian, L. (2016). Foreign language affects the contribution of intentions and outcomes to moral judgment. *Cognition*, *154*, 34–39. http://doi.org/10.1016/j.cognition.2016.05.010

Gitelman, D. R., Penny, W. D., Ashburner, J., & Friston, K. (2003). Modeling regional and psychophysiologic interactions in fMRI: the importance of hemodynamic deconvolution. *Neuroimage*, *19*(1), 200–207. http://doi.org/10.1016/S1053-8119(03)00058-2

Glaser, D. E., & Friston, K. (2004). Variance components. In R. S. J. Frackowiak, J. T. Ashburner, W. D. Penny, S. Zeki, K. J. Friston, C. D. Frith, … C. J. Price (Eds.), *Human brain function* (2nd ed., pp. 781–791). London: Academic Press.

Gray, K., & Schein, C. (2012). Two Minds Vs. Two Philosophies: Mind Perception Defines Morality and Dissolves the Debate Between Deontology and Utilitarianism. *Review of Philosophy and Psychology*, *3*(3), 405–423. http://doi.org/10.1007/s13164-012-0112-5

Griffanti, L., Douaud, G., Bijsterbosh, J., Evangelisti, S., Alfaro-Almagro, F., Glasser, M. F., … Smith, S. M. (2016). Hand classification of fMRI ICA noise components. *NeuroImage*. http://doi.org/10.1016/j.neuroimage.2016.12.036

Han, H., & Glenn, A. L. (2017). Evaluating Methods of Correcting for Multiple Comparisons Implemented in SPM12 in Social Neuroscience fMRI Studies: An Example from Moral Psychology. *Social Neuroscience*. http://doi.org/http://www.sciencedirect.com/science/article/pii/S0022096517301807

Hein, G., Silani, G., Preuschoff, K., Batson, C. D., & Singer, T. (2010). Neural responses to ingroup and outgroup members' suffering predict individual differences in costly helping. *Neuron*, *68*(1), 149–160. http://doi.org/10.1016/j.neuron.2010.09.003

Henson, R. (2007). Efficient experimental design for fMRI. In K. Friston, J. Ashburner, S. Kiebel, T. Nichols, & W. Penny (Eds.), *Statistical Parametric Mapping: The Analysis of Functional Brain Images* (pp. 193–210). London: Elsevier.

Henson, R., Rugg, M., & Friston, K. (2001). The choice of basis functions in event-related fMRI. *Neuroimage, 13*(6), 149. http://doi.org/10.1016/S1053-8119(01)91492-2

Hess, U., Blaison, C., & Dandeneau, S. (2017). The impact of rewards on empathic accuracy and emotional mimicry. *Motivation and Emotion*, *41*(1), 107–112. http://doi.org/10.1007/s11031-016-9590-6

Hyatt, C. J., Calhoun, V. D., Pearlson, G. D., & Assaf, M. (2015). Specific default mode subnetworks support mentalizing as revealed through opposing network recruitment by social and semantic FMRI tasks. *Human Brain Mapping*, *36*(8), 3047–63. http://doi.org/10.1002/hbm.22827

Immordino-Yang, M. H., McColl, A., Damasio, H., & Damasio, A. (2009). Neural correlates of admiration and compassion. *Proceedings of the National Academy of Sciences*, *106*(19), 8021–6. http://doi.org/10.1073/pnas.0810363106

Inbar, Y., Pizarro, D., & Cushman, F. (2012). Benefiting from misfortune: when harmless actions are judged to be morally blameworthy. *Personality and Social Psychology Bulletin*, *38*(1), 52–62. http://doi.org/10.1177/0146167211430232

Inglis, B. (2015). A checklist for fMRI acquisition methods reporting in the literature. *The Winnower*, *4*, e143191.17127. http://doi.org/10.15200/winn.143191.17127

Jordan, M. R., Amir, D., & Bloom, P. (2016). Are Empathy and Concern Psychologically Distinct? *Emotion*, *16*(8), 1107–1116. http://doi.org/10.1037/emo0000228

Kanske, P., Böckler, A., Trautwein, F.-M., Lesemann, F. H. P., & Singer, T. (2016). Are strong empathizers better mentalizers? Evidence for independence and interaction between the routes of social cognition. *Social Cognitive and Affective Neuroscience*, *11*(9), 1383–1392. http://doi.org/10.1093/scan/nsw052

Keysers, C., Kaas, J. H., & Gazzola, V. (2010). Somatosensation in social perception. *Nature Reviews. Neuroscience*, *11*(6), 417–428. http://doi.org/10.1038/nrn2919

Kogut, T. (2011). The role of perspective taking and emotions in punishing identified and unidentified wrongdoers. *Cognition & Emotion*, *25*(8), 1491–1499. http://doi.org/10.1080/02699931.2010.547563

Konis, D., Haran, U., Saporta, K., & Ayal, S. (2016). A Sorrow Shared is a Sorrow Halved: Moral Judgments of Harm to Single versus Multiple Victims. *Frontiers in Psychology*, *7*, 1142. http://doi.org/10.3389/fpsyg.2016.01142

Koster-Hale, J., Bedny, M., & Saxe, R. (2014). Thinking about seeing: Perceptual sources of knowledge are encoded in the theory of mind brain regions of sighted and blind adults. *Cognition*, *133*(1), 65–78. http://doi.org/10.1016/j.cognition.2014.04.006

Krishnan, A., Woo, C.-W., Chang, L. J., Ruzic, L., Gu, X., López-Solà, M., … Wager, T. D. (2016). Somatic and vicarious pain are represented by dissociable multivariate brain patterns. *eLife*, *5*, e15166. http://doi.org/10.7554/eLife.15166

Krueger, F., & Hoffman, M. (2016). The Emerging Neuroscience of Third-Party Punishment. *Trends in Neurosciences, 39*(8), 499–501. http://doi.org/10.1016/j.tins.2016.06.004

Kutner, M. H., Nachtsheim, C. J., Neter, J., & Li, W. (2004). *Applied Linear Statistical Models*. *McGraw-*

Hill/Irwin (5th ed.). McGraw-Hill/Irwin.

Lamm, C., Batson, C. D., & Decety, J. (2007). The neural substrate of human empathy: effects of perspective-taking and cognitive appraisal. *Journal of Cognitive Neuroscience*, *19*(1), 42–58. http://doi.org/10.1162/jocn.2007.19.1.42

Lamm, C., Bukowski, H., & Silani, G. (2016). From shared to distinct self-other representations in empathy: evidence from neurotypical function and socio-cognitive disorders. *Philosophical Transactions of the Royal Society of London. Series B, Biological Sciences*, *371*(1686), 20150083. http://doi.org/10.1098/rstb.2015.0083

Lamm, C., Decety, J., & Singer, T. (2011). Meta-analytic evidence for common and distinct neural networks associated with directly experienced pain and empathy for pain. *NeuroImage*, *54*(3), 2492–502. http://doi.org/10.1016/j.neuroimage.2010.10.014

Laurent, S. M., Nuñez, N. L., & Schweitzer, K. A. (2015). The influence of desire and knowledge on perception of each other and related mental states, and different mechanisms for blame. *Journal of Experimental Social Psychology*, *60*, 27–38. http://doi.org/10.1016/j.jesp.2015.04.009

Legrain, V., Iannetti, G. D., Plaghki, L., & Mouraux, A. (2011). The pain matrix reloaded: A salience detection system for the body. *Progress in Neurobiology*, *93*(1), 111–124. http://doi.org/10.1016/j.pneurobio.2010.10.005

Liberati, G., Klöcker, A., Safronova, M. M., Ferrão Santos, S., Ribeiro Vaz, J.-G., Raftopoulos, C., & Mouraux, A. (2016). Nociceptive Local Field Potentials Recorded from the Human Insula Are Not Specific for Nociception. *PLoS Biology*, *14*(1), e1002345. http://doi.org/10.1371/journal.pbio.1002345

Lieberman, M. D., & Cunningham, W. A. (2009). Type I and Type II error concerns in fMRI research: Re-balancing the scale. *Social Cognitive and Affective Neuroscience*, *4*(4), 423–428. http://doi.org/10.1093/scan/nsp052

Lieberman, M. D., & Eisenberger, N. I. (2015). The dorsal anterior cingulate cortex is selective for pain: Results from large-scale reverse inference. *Proceedings of the National Academy of Sciences, 112*(49), 15250–15255. http://doi.org/10.1073/pnas.1515083112

Lindquist, M. (2008). The Statistical Analysis of fMRI Data. *Statistical Science*, *23*(4), 439–464. http://doi.org/10.1214/09-STS282

Lindquist, M., Meng Loh, J., Atlas, L., & Wager, T. D. (2009). Modeling the hemodynamic response function in fMRI: efficiency, bias and mis-modeling. *NeuroImage*, *45*(1 Suppl), S187-98. http://doi.org/10.1016/j.neuroimage.2008.10.065

Liu, T. T. (2016). Noise contributions to the fMRI signal: An Overview. *NeuroImage*, *143*, 141–151. http://doi.org/10.1016/j.neuroimage.2016.09.008

Lucas, B. J., Galinsky, A. D., & Murnighan, K. J. (2016). An Intention-Based Account of Perspective-Taking. *Personality and Social Psychology Bulletin*, *42*(11), 1480–1489. http://doi.org/10.1177/0146167216664057

Malle, B. F., Guglielmo, S., & Monroe, A. E. (2014). A Theory of Blame. *Psychological Inquiry*, *25*(2), 147–186. http://doi.org/10.1080/1047840X.2014.877340

Martin, J. W., & Cushman, F. (2016). Why we forgive what can’t be controlled. *Cognition*, *147*, 133–143. http://doi.org/10.1016/j.cognition.2015.11.008

Mazzocco, P., Alicke, M., & Davis, T. (2004). On the robustness of outcome bias: No constraint by prior

culpability. *Basic and Applied Social Psychology*, *26*(2–3), 131–146. http://doi.org/10.1080/01973533.2004.9646401

McLaren, D. G., Ries, M. L., Xu, G., & Johnson, S. C. (2012). A generalized form of context-dependent psychophysiological interactions (gPPI): A comparison to standard approaches. *NeuroImage*, *61*(4), 1277–1286. http://doi.org/10.1016/j.neuroimage.2012.03.068

Meindl, P., & Graham, J. (2014). Know Thy Participant: The Trouble with Nomothetic Assumptions in Moral Psychology. In H. Sarkissian & J. C. Wright (Eds.), *Advances in Experimental Moral Psychology* (pp. 233–252). London: Bloomsbury.

Menon, V., & Uddin, L. Q. (2010). Saliency, switching, attention and control: a network model of insula function. *Brain Structure & Function*, *214*(5–6), 655–67. http://doi.org/10.1007/s00429-010-0262-0

Miller, R., & Cushman, F. (2017). Empathy, compassion, and moral judgment: The dissociable effects of other-oriented emotions across help and harm contexts. *PLoS ONE*.

Monti, M. (2011). Statistical Analysis of fMRI Time-Series: A Critical Review of the GLM Approach. *Frontiers in Human Neuroscience*, *5*, 28. http://doi.org/10.3389/fnhum.2011.00028

Mumford, J., Poline, J.-B., & Poldrack, R. (2015). Orthogonalization of Regressors in fMRI Models. *PLoS ONE*, *10*(4), e0126255. http://doi.org/10.1371/journal.pone.0126255

Murray, D., & Lombrozo, T. (2017). Effects of Manipulation on Attributions of Causation, Free Will, and Moral Responsibility. *Cognitive Science*, *41*(2), 447–481. http://doi.org/10.1111/cogs.12338

Mutschler, I., Wieckhorst, B., Kowalevski, S., Derix, J., Wentlandt, J., Schulze-Bonhage, A., & Ball, T. (2009). Functional organization of the human anterior insular cortex. *Neuroscience Letters*, *457*(2), 66–70. http://doi.org/10.1016/j.neulet.2009.03.101

Ngo, L., Kelly, M., Coutlee, C. G., Carter, R. M., Sinnott-Armstrong, W., & Huettel, S. A. (2015). Two Distinct Moral Mechanisms for Ascribing and Denying Intentionality. *Scientific Reports*, *5*, 17390. http://doi.org/10.1038/srep17390

Nieto-Castañón, A., & Fedorenko, E. (2012). Subject-specific functional localizers increase sensitivity and functional resolution of multi-subject analyses. *NeuroImage*, *63*(3), 1646–69. http://doi.org/10.1016/j.neuroimage.2012.06.065

O'brien, R. M. (2007). A Caution Regarding Rules of Thumb for Variance Inflation Factors. *Quality & Quantity*, *41*(5), 673–690. http://doi.org/10.1007/s11135-006-9018-6

O'Reilly, J. X., Woolrich, M. W., Behrens, T. E. J., Smith, S. M., & Johansen-Berg, H. (2012). Tools of the trade: Psychophysiological interactions and functional connectivity. *Social Cognitive and Affective Neuroscience*, *7*(5), 604–609. http://doi.org/10.1093/scan/nss055

Patil, I., & Silani, G. (2014). Alexithymia increases moral acceptability of accidental harms. *Journal of Cognitive Psychology*, *26*(5), 597–614. http://doi.org/10.1080/20445911.2014.929137

Patil, I., Young, L., Sinay, V., & Gleichgerrcht, E. (2017). Elevated moral condemnation of third-party violations in multiple sclerosis patients. *Social Neuroscience*, *12*(3), 308–329. http://doi.org/10.1080/17470919.2016.1175380

Pauli, R., Bowring, A., Reynolds, R., Chen, G., Nichols, T. E., & Maumet, C. (2016). Exploring fMRI Results Space: 31 Variants of an fMRI Analysis in AFNI, FSL, and SPM. *Frontiers in Neuroinformatics*, *10*, 24. http://doi.org/10.3389/fninf.2016.00024

Pernet, C. (2014). Misconceptions in the use of the General Linear Model applied to functional MRI: a tutorial for junior neuro-imagers. *Frontiers in Neuroscience*, *8*, 1.

http://doi.org/10.3389/fnins.2014.00001

Phillips, J., & Shaw, A. (2015). Manipulating Morality: Third-Party Intentions Alter Moral Judgments by Changing Causal Reasoning. *Cognitive Science*, *39*(6), 1320–47. http://doi.org/10.1111/cogs.12194

Poldrack, R. (2007). Region of interest analysis for fMRI. *Social Cognitive and Affective Neuroscience, 2*(1), 67–70. http://doi.org/10.1093/scan/nsm006

Poldrack, R., Baker, C. I., Durnez, J., Gorgolewski, K. J., Matthews, P. M., Munafò, M. R., … Yarkoni, T. (2017). Scanning the horizon: towards transparent and reproducible neuroimaging research. *Nature Reviews Neuroscience*, *18*, 115–126. http://doi.org/10.1038/nrn.2016.167

Poldrack, R., Fletcher, P. C., Henson, R., Worsley, K. J., Brett, M., & Nichols, T. E. (2008). Guidelines for reporting an fMRI study. *NeuroImage*, *40*(2), 409–414. http://doi.org/10.1016/j.neuroimage.2007.11.048

Poldrack, R., Mumford, J., & Nichols, T. E. (2011). *Handbook of functional MRI data analysis* (1st ed.). New York: Cambridge University Press.

Power, J. D. (2016). A simple but useful way to assess fMRI scan qualities. *NeuroImage*. http://doi.org/10.1016/j.neuroimage.2016.08.009

Power, J. D., Barnes, K., Snyder, A., Schlaggar, B., & Petersen, S. (2012). Spurious but systematic correlations in functional connectivity MRI networks arise from subject motion. *NeuroImage*, *59*(3), 2142–2154. http://doi.org/10.1016/j.neuroimage.2011.10.018

Raichle, M., & Mintun, M. (2006). Brain work and brain imaging. *Annual Review of Neuroscience*, *29*, 449–76. http://doi.org/10.1146/annurev.neuro.29.051605.112819

Ridgway, G. R., Henley, S. M. D., Rohrer, J. D., Scahill, R. I., Warren, J. D., & Fox, N. C. (2008). Ten simple rules for reporting voxel-based morphometry studies. *NeuroImage*, *40*(4), 1429–1435. http://doi.org/10.1016/j.neuroimage.2008.01.003

Ridgway, G. R., Litvak, V., Flandin, G., Friston, K., & Penny, W. D. (2012). The problem of low variance voxels in statistical parametric mapping; a new hat avoids a "haircut." *NeuroImage*, *59*(3), 2131–2141. http://doi.org/10.1016/j.neuroimage.2011.10.027

Salomons, T. V., Iannetti, G. D., Liang, M., & Wood, J. N. (2016). The "Pain Matrix" in Pain-Free Individuals. *JAMA Neurology*, *73*(6), 755–6. http://doi.org/10.1001/jamaneurol.2016.0653

Segerdahl, A. R., Mezue, M., Okell, T. W., Farrar, J. T., & Tracey, I. (2015). The dorsal posterior insula subserves a fundamental role in human pain. *Nature Neuroscience*, *18*(4), 499–500. http://doi.org/10.1038/nn.3969

Shenhav, A., & Greene, J. (2014). Integrative moral judgment: dissociating the roles of the amygdala and ventromedial prefrontal cortex. *The Journal of Neuroscience*, *34*(13), 4741–9. http://doi.org/10.1523/JNEUROSCI.3390-13.2014

Singer, T., Seymour, B., O'Doherty, J. P., Stephan, K. E., Dolan, R. J., & Frith, C. D. (2006). Empathic neural responses are modulated by the perceived fairness of others. *Nature*, *439*(7075), 466–9. http://doi.org/10.1038/nature04271

Smith, D. V., Gseir, M., Speer, M. E., & Delgado, M. R. (2016). Toward a cumulative science of functional integration: A meta-analysis of psychophysiological interactions. *Human Brain Mapping*, *37*(8), 2904–17. http://doi.org/10.1002/hbm.23216

Theriault, J., & Young, L. (2014). Taking an "Intentional Stance" on Moral Psychology. In J. Systma (Ed.), *Advances in Experimental Philosophy of Mind* (pp. 101–124). Continuum Press.

Treadway, M. T., Buckholtz, J. W., Martin, J. W., Jan, K., Asplund, C. L., Ginther, M. R., … Marois, R. (2014). Corticolimbic gating of emotion-driven punishment. *Nature Neuroscience, 17*(9), 1270–5. http://doi.org/10.1038/nn.3781

Trémolière, B., & Djeriouat, H. (2016). The sadistic trait predicts minimization of intention and causal responsibility in moral judgment. *Cognition*, *146*, 158–171. http://doi.org/10.1016/j.cognition.2015.09.014

Tusche, A., Bockler, A., Kanske, P., Trautwein, F.-M., & Singer, T. (2016). Decoding the Charitable Brain: Empathy, Perspective Taking, and Attention Shifts Differentially Predict Altruistic Giving. *Journal of Neuroscience*, *36*(17), 4719–4732. http://doi.org/10.1523/JNEUROSCI.3392-15.2016

Uddin, L. Q. (2014). Salience processing and insular cortical function and dysfunction. *Nature Reviews Neuroscience*, *16*(1), 55–61. http://doi.org/10.1038/nrn3857

Vergara, V. M., Mayer, A., Damaraju, E., Hutchison, K., & Calhoun, V. (2016). The effect of preprocessing pipelines in subject classification and detection of abnormal resting state functional network connectivity using group ICA. *NeuroImage*, *145*, 365–376. http://doi.org/10.1016/j.neuroimage.2016.03.038

Vul, E., & Pashler, H. (2017). Suspiciously high correlations in brain imaging research. In S. O. Lilienfeld & I. D. Waldman (Eds.), *Psychological science under scrutiny: Recent challenges and proposed solutions* (pp. 196–220). New York: Wiley.

Wager, T. D., Atlas, L. Y., Botvinick, M. M., Chang, L. J., Coghill, R. C., Davis, K. D., … Yarkoni, T. (2016). Pain in the ACC? *Proceedings of the National Academy of Sciences*, *113*(18), E2474–E2475. http://doi.org/10.1073/pnas.1600282113

Weiskopf, N., Hutton, C., Josephs, O., & Deichmann, R. (2006). Optimal EPI parameters for reduction of susceptibility-induced BOLD sensitivity losses: a whole-brain analysis at 3 T and 1.5 T. *Neuroimage*, *33*(2), 493–504.

Wilke, M. (2014). Isolated Assessment of Translation or Rotation Severely Underestimates the Effects of Subject Motion in fMRI Data. *PLoS ONE*, *9*(10), e106498. http://doi.org/10.1371/journal.pone.0106498

Woo, C. W., Krishnan, A., & Wager, T. D. (2014). Cluster-extent based thresholding in fMRI analyses: Pitfalls and recommendations. *NeuroImage*, *91*, 412–419. http://doi.org/10.1016/j.neuroimage.2013.12.058

Woolfolk, R. L., Doris, J. M., & Darley, J. M. (2006). Identification, situational constraint, and social cognition: Studies in the attribution of moral responsibility. *Cognition*, *100*(2), 283–301. http://doi.org/10.1016/j.cognition.2005.05.002

Yamada, M., Camerer, C. F., Fujie, S., Kato, M., Matsuda, T., Takano, H., … Takahashi, H. (2012). Neural circuits in the brain that are activated when mitigating criminal sentences. *Nature Communications*, *3*, 759. http://doi.org/10.1038/ncomms1757

Yang, X.-F., Bossmann, J., Schiffhauer, B., Jordan, M., & Immordino-Yang, M. H. (2012). Intrinsic Default Mode Network Connectivity Predicts Spontaneous Verbal Descriptions of Autobiographical Memories during Social Processing. *Frontiers in Psychology*, *3*, 592. http://doi.org/10.3389/fpsyg.2012.00592

Young, L., Camprodon, J. A., Hauser, M., Pascual-Leone, A., & Saxe, R. (2010). Disruption of the right temporoparietal junction with transcranial magnetic stimulation reduces the role of beliefs in moral judgments. *Proceedings of the National Academy of Sciences*, *107*(15), 6753–6758. http://doi.org/10.1073/pnas.0914826107

Young, L., Cushman, F., Hauser, M., & Saxe, R. (2007). The neural basis of the interaction between theory of mind and moral judgment. *Proceedings of the National Academy of Sciences*, *104*(20), 8235–40. http://doi.org/10.1073/pnas.0701408104

Young, L., Nichols, S., & Saxe, R. (2010). Investigating the Neural and Cognitive Basis of Moral Luck: It's Not What You Do but What You Know. *Review of Philosophy and Psychology*, *1*(3), 333–349. http://doi.org/10.1007/s13164-010-0027-y

Young, L., & Saxe, R. (2008). The neural basis of belief encoding and integration in moral judgment. *NeuroImage*, *40*(4), 1912–20. http://doi.org/10.1016/j.neuroimage.2008.01.057

Yu, H., Li, J., & Zhou, X. (2015). Neural Substrates of Intention–Consequence Integration and Its Impact on Reactive Punishment in Interpersonal Transgression. *The Journal of Neuroscience*, *35*(12), 4917–4925. http://doi.org/10.1523/JNEUROSCI.3536-14.2015

Zaki, J., & Ochsner, K. (2012). The neuroscience of empathy: progress, pitfalls and promise. *Nature Neuroscience*, *15*(5), 675–680. http://doi.org/10.1038/nn.3085

Zaki, J., Wager, T. D., Singer, T., Keysers, C., & Gazzola, V. (2016). The Anatomy of Suffering: Understanding the Relationship between Nociceptive and Empathic Pain. *Trends in Cognitive Sciences*, *20*(4), 249–59. http://doi.org/10.1016/j.tics.2016.02.003

Appendix

Popcorn

Matteo is babysitting at his cousin's house. They're eating popcorn. Suddenly they hear a loud beeping noise coming from the kitchen. Matteo's cousin gets up to investigate.

The beeping noise is from a smoke detector responding to leftover smoke coming from the microwave where Matteo and his cousin burned popcorn. The kitchen is not dangerous.

The beeping noise is from a carbon monoxide detector in the kitchen. Deadly carbon monoxide is escaping from the furnace under the kitchen. The kitchen is now dangerous.

Matteo thinks that the noise is the smoke detector as they had just burned a bag of popcorn. He thinks going to the kitchen to turn off the beeping device is a good idea.

Matteo thinks that the noise is a carbon monoxide detector because of the sound. He thinks going to the kitchen to turn off the beeping could cause his cousin to pass out.

Matteo watches quietly while his cousin goes into the kitchen. His cousin returns laughing, saying that the burnt popcorn had made the smoke alarm go off.

Matteo watches quietly while his cousin goes into the kitchen. His cousin goes into the kitchen and, while fidgeting with the device, passes out from carbon monoxide inhalation.

How morally acceptable was Matteo's behavior?

How much punishment does Matteo deserve for his behavior?

How much blame does Matteo deserve?

Malaria Pond /African Pond

Pietro is traveling in Africa with a friend. His friend sees a pond and wants to go swimming in it because it is very hot. His friend begins to walk toward the pond.

The pond is a good place for tourists. It does not contain any disease-carrying organisms. The water is unusually clean, so it is safe to swim in.

Malarial mosquitoes actually live in the pond. A single bite is enough to create an infection, so the pond is unsafe to swim in.

Pietro thinks that it is safe to swim in the pond because other tourists around them are doing it too and are obviously having fun.

Pietro thinks that it is unsafe to swim in the pond because Africa is known for malarial mosquitoes, and mosquitoes congregate around water.

Pietro encourages his friend to swim in the pond. His friend loves the cool water and has a great time splashing around.

Pietro encourages his friend to swim in the pond. His friend is bitten by several mosquitoes and contracts malaria.

How morally acceptable was Pietro's behavior?

How much punishment does Pietro deserve for his behavior?

How much blame does Pietro deserve?

Spinach

Luca is grocery shopping for his grandmother who adores spinach. Recently there had been incidents of E. coli (type of a germ) contamination of bagged spinach leading to a recall of all bagged spinach.

Bagged spinach has been restocked at many markets. It is 100% safe to eat and no longer contaminated with E. coli.

Bagged spinach has been restocked at many markets. But some inspections aren't thorough and contaminated batches are still being missed.

At the market, Luca sees that bagged spinach is being carried again. He thinks that it's perfectly safe now because of some official-looking information by the spinach.

At the market, Luca sees that bagged spinach is being carried again. He thinks that bagged spinach may still be contaminated because of an incident just that day in his town.

Luca buys his grandmother bagged spinach. His grandmother cooks some for dinner that evening. The meal is healthy and delicious.

Luca buys his grandmother bagged spinach. His grandmother cooks some for dinner that evening and ends up in the hospital, violently ill.

How morally acceptable was Luca's behavior?

How much punishment does Luca deserve for his behavior?

How much blame does Luca deserve?

Peanut allergy

Susanna teaches first grade. She is reviewing the student health forms to see who has food allergies, so she can assign classrooms appropriately. One of the students is missing a form.

The student used to have a peanut allergy but has outgrown it. So, now, she is completely fine being around peanuts and even eating food that contains peanuts.

The student didn't use to have a peanut allergy but has developed a severe one. So, now, even when she's in a room where someone's eating peanuts, she goes into shock.

Susanna calls the student's home to find out whether she has any allergies and talks to the student's brother. Susanna thinks that the student is allergy-free.

Susanna calls the student's home to find out whether she has any allergies and talks to the student's brother. Susanna thinks that the student is allergic to peanuts.

Susanna puts the student in the classroom for people with no food allergies. The student sits next to someone eating a peanut butter sandwich and is totally fine.

Susanna puts the student in the classroom for people with no food allergies. The student sits next to someone eating a peanut butter sandwich and soon goes into shock.

How morally acceptable was Susanna's behavior?

How much punishment does Susanna deserve for her behavior?

How much blame does Susanna deserve?

Rabies/Rabid dog

Chiara works at the pound. Several new dogs have just come in. A lady comes in, interested in taking one of the new dogs home with her.

The dogs are in fact very healthy and active and will make great pets for anyone who loves dogs.

The dogs are very sick with rabies and will make their owners sick too by biting them.

Chiara talks with one of the other people at the pound. Chiara thinks that the new dogs have been through a thorough health inspection and will make good pets.

Chiara talks with one of the other people at the pound. Chiara thinks that the new dogs all failed their health inspection and are infected with rabies.

Chiara gives the lady one of the new dogs. It is in perfect health, and the lady bonds immediately with her new pet.

Chiara gives the lady one of the new dogs. It is infected with rabies and bites the lady on the neck on the very first day.

How morally acceptable was Chiara's behavior?

How much punishment does Chiara deserve for her behavior?

How much blame does Chiara deserve?

Meatloaf

Rebecca works at a day care. For lunch, Rebecca plans to make meatloaf for all of the children. She opens a package of ground beef to make the meatloaf.

The meat is actually completely fresh, because the package was tightly sealed while stored in the fridge. So the meat is safe to eat.

The meat has some invisible but very deadly bacteria on it, because of a small tear in the seal. So the meat is not safe to eat.

Rebecca thinks that ground beef is perfectly safe to eat because the expiration date on it is two weeks away from now.

Rebecca thinks that the ground beef is not safe to eat, because the expiration date on it passed two weeks ago.

Rebecca makes meatloaf out of the ground beef and serves it to the children. The children eat the meal and find it delicious. They are quite full afterwards.

Rebecca makes meatloaf out of the ground beef and serves it to the children. The children suffer from diarrhea, and are hospitalized with severe food poisoning.

How morally acceptable was Rebecca's behavior?

How much punishment does Rebecca deserve for her behavior?

How much blame does Rebecca deserve?

Seatbelt/Amusement Park

Giovanni works at an old and very small amusement park. His job is to operate one of the rides. One of the customers finds the seatbelts uncomfortable, so he leaves them unfastened.

The ride is actually quite tame, so wearing seatbelts is really unnecessary. Nobody has ever been injured before riding without seatbelts.

The ride is actually quite bumpy and dangerous, so anyone not wearing a seatbelt risks getting tossed and getting hurt.

Having heard from his friend who operated this ride before, Giovanni thinks that the seatbelts aren't necessary at all for this ride.

Having heard from his friend who operated this ride before, Giovanni thinks that the seatbelts are totally necessary for this ride.

Giovanni keeps his mouth shut as the customer sits without fastening his seatbelt. The customer enjoys the ride just like everyone else.

Giovanni keeps his mouth shut as the customer sits without fastening his seatbelt. The customer gets tossed during the ride and suffers a severe concussion.

How morally acceptable was Giovanni's behavior?

How much punishment does Giovanni deserve for his behavior?

How much blame does Giovanni deserve?

Teenagers/Skiing

Jessica is skiing somewhere in the Alps. She sees a group of teenagers about to ski down a slope that feeds into a dangerously rocky section of the mountain.

The teenagers are professional skiers and know how to ski down the most difficult slopes under any conditions.

The teenagers are only novice skiers and do not know how to ski very well at all especially on rocky terrain.

Jessica believes that the teenagers must be expert skiers, based on their impressive skis and equipment, and that they must know how to maneuver around the rocks.

Jessica believes that the teenagers are only novice skiers, based on their cheap ski rentals, and that they must not know how to maneuver around the rocks.

Jessica skis past the teenagers without saying anything. The teenagers ski down the slope and have an awesome time.

Jessica skis past the teenagers without saying anything. The teenagers ski down the slope, crash into the sharp rocks, and get badly injured.

How morally acceptable was Jessica's behavior?

How much punishment does Jessica deserve for her behavior?

How much blame does Jessica deserve?

Ham sandwich

Veronica and a friend are having lunch on Saturday. They are making sandwiches with stuff from Veronica's fridge, when Veronica's friend says she'd prefer a ham sandwich.

The ham in the refrigerator is high quality and was purchased just a day ago. It's fresh and ready to be used in sandwiches.

The ham in the refrigerator was purchased a week ago and has since become slightly spoiled, so it's not safe to eat.

Since Veronica's mom always shops for meats only on Fridays, Veronica thinks that the ham in the refrigerator is fresh and is safe to eat.

Since Veronica's mom usually shops for meat only on Sundays, Veronica thinks that the ham in the refrigerator is very old and not safe to eat.

Veronica makes a ham sandwich for her friend. Her friend enjoys the sandwich, and they go outside to play afterwards.

Veronica makes a ham sandwich for her friend. Her friend eats the sandwich and throws up all night from food poisoning.

How morally acceptable was Veronica's behavior?

How much punishment does Veronica deserve for her behavior?

How much blame does Veronica deserve?

Safety Cord/Rock climbing

Cristina works at a rock-climbing shop. She is unpacking some equipment when a customer walks in, looking for a safety cord.

The safety cords from the new company are incredibly well made. They come with a lifetime guarantee and work very well for expert rock-climbers.

The safety cords from the new company are about to be recalled. They won't hold anyone who is rock-climbing for longer than 20 minutes.

Cristina sees that the equipment is from a new company that a rock-climbing friend of hers finds really reliable. So, Cristina thinks that their safety cords are solid.

Cristina sees that the equipment is from a new company that a rock-climbing friend of hers finds really unreliable. So, Cristina thinks that their safety cords are untrustworthy.

Cristina sells the customer one of the new safety cords. The safety cord serves the customer well on his next rock-climbing expedition. The customer is very pleased.

Cristina sells the customer one of the new safety cords. The safety cord snaps during the customer's next rock-climbing expedition. The customer falls 50 meters.

How morally acceptable was Cristina’s behavior?

How much punishment does Cristina deserve for her behavior?

How much blame does Cristina deserve?

Sesame seeds

Caterina is a waitress preparing to take a meal out to a customer's table. The customer is with his friends, and he orders a meal that calls for sesame seeds.

The customer happens to love sesame seeds and that he will have no problem at all if he eats the sesame seeds in his meal.

The customer happens to be highly allergic to sesame seeds and that he will likely be very sick if he eats the sesame seeds.

After overhearing part of the customer's conversation with his friends, Caterina believes that the customer loves sesame seeds.

After overhearing part of the customer's conversation with his friends, Caterina believes that the customer is highly allergic to sesame seeds.

Caterina puts the sesame seeds in. The customer enjoys his meal and is fine.

Caterina puts the sesame seeds in. The customer eats his meal and ends up in the hospital with a very bad allergic reaction.

How morally acceptable was Caterina's behavior?

How much punishment does Caterina deserve for her behavior?

How much blame does Caterina deserve?

Chemical Plant/Coffee

Olga and her friend are taking a tour of a chemical plant. When Olga goes over to the coffee machine to pour some coffee, Olga's friend asks for some sugar in hers. There is white powder in a container by the coffee.

The white powder is just the regular sugar that the scientists use every day, and is therefore perfectly safe to have in coffee.

The white powder is a very toxic left behind by a scientist, and therefore deadly when ingested in any form.

The container is labeled "sugar", so Olga believes that the white powder by the coffee is sugar left out by the kitchen staff.

The container is labeled "toxic", so Olga believes that the white powder is toxic substance left behind by a scientist.

Olga puts the substance in her friend's coffee. Her friend drinks the coffee and is fine.

Olga puts the substance in her friend's coffee. Her friend drinks the coffee and starts vomiting blood.

How morally acceptable was Olga's behavior?

How much punishment does Olga deserve for her behavior?

How much blame does Olga deserve?

Bridge/River-Bridge

Eugenio and his girlfriend are hiking. They come across a long narrow bridge that spans a shallow river.

The bridge happens to be extremely sturdy and can easily carry the weight of many people at once.

The bridge happens to be extremely unsteady and cannot carry the weight of even one very light person.

Eugenio believes that whoever walks on the bridge will cross the river quite safely because the bridge is maintained by the national park.

Eugenio believes that whoever walks on the bridge will break the bridge and fall into the river because the bridge looks unsteady and old.

Eugenio says nothing as his girlfriend starts walking across the bridge. His girlfriend reaches the other side safely.

Eugenio says nothing as his girlfriend starts walking across the bridge. His girlfriend breaks the bridge, falls down in the river, and breaks her legs.

How morally acceptable was Eugenio's behavior?

How much punishment does Eugenio deserve for his behavior?

How much blame does Eugenio deserve?

Pool

Matilda is walking by a neighbour's swimming pool when she sees a child about to dive in.
The child is about to dive into the deep end and have fun swimming around and playing all day.

The child is about to dive into the shallow end and smack his head very hard on the concrete bottom of the pool.

Because of a label on the side of the pool, Matilda believes that the child is about to dive safely into the deep end and swim around.

Because of a warning sign at the side of the pool, Matilda believes that the child is about to dive into shallow water and break his neck.

Matilda walks by, without saying anything to the child. The child dives in and is fine.

Matilda walks by, without saying anything to the child. The child dives in and breaks his neck.

How morally acceptable was Matilda's behavior?

How much punishment does Matilda deserve for her behavior?

How much blame does Matilda deserve?

Mushrooms

Carlotta and an acquaintance are camping in the woods. Carlotta spots some wild mushrooms growing along the campsite. Carlotta studies the mushrooms and consults her plant life guide.

The mushrooms happen to be edible and delicious. They are the kind that one can buy in the supermarket and put in salad.

The mushrooms happen to be lethal and tasteless. They are the kind that result in painful convulsions and ultimately death.

Carlotta sees a picture of an edible mushroom in her book that looks just like these mushrooms at the campsite, so she believes that the mushrooms are edible.

Carlotta sees a picture of a lethal mushroom in her book that looks just like these mushrooms at the campsite, so she believes that the mushrooms are lethal.

Carlotta offers the mushrooms to her acquaintance. Her acquaintance eats them and finds them very tasty.

Carlotta offers the mushrooms to her acquaintance. Her acquaintance eats them and starts convulsing after 10 minutes.

How morally acceptable was Carlotta's behavior?

How much punishment does Carlotta deserve for her behavior?

How much blame does Carlotta deserve?

Latex

Valentina sees a patient for a routine check-up. Valentina normally uses latex gloves to examine her patients.

This patient has been in contact with latex before and, like most people, does not have an allergy to latex.

This patient has a lethal allergy to latex and cannot be examined with latex without immediately going into shock.

Having read the information in the patient's records, Valentina believes that the patient is not allergic to latex and using latex gloves to examine him poses no risk to his health.

Having read the information in the patient's records, Valentina believes that the patient is highly allergic to latex and using latex gloves to examine him will result in his death.

Valentina puts on latex gloves and examines her patient. The patient is fine.

Valentina puts on latex gloves and examines her patient. The patient goes into shock.

How morally acceptable was Valentina's behavior?

How much punishment does Valentina deserve for her behavior?

How much blame does Valentina deserve?

Motorboat

Alessandro is driving his motorboat in the bay, on his way home. He spots a swimmer in the far distance.

The swimmer is in fact fine and is waving at Alessandro for fun as he does with people in the bay.

The swimmer is in fact drowning and is waving frantically at Alessandro for help.

Alessandro sees the swimmer waving and believes that the swimmer is waving an enthusiastic "hello".

Alessandro sees the swimmer waving and believes that the swimmer is drowning and waving desperately for help because his arm motions are so emphatic.

Alessandro drives home, leaving the swimmer behind. The swimmer is fine.

Alessandro drives home, leaving the swimmer behind. The swimmer drowns.

How morally acceptable was Alessandro's behavior?

How much punishment does Alessandro deserve for his behavior?

How much blame does Alessandro deserve?

Asthma

Erica is driving home when she sees a runner by the side of the road. The runner is bent over and has one hand on his chest. There is an empty asthma inhaler on the ground.

The runner is just short of breath and has stopped to rest for a moment before continuing his long jog.

The runner is having a serious asthma attack and needs to get to a hospital immediately before he goes into shock.

Because Erica doesn't see the inhaler, she believes that the runner has just stopped to catch his breath and will continue his jog shortly.

Because Erica sees the inhaler and the gasping runner, she believes that the runner is having an asthma attack and must get to a hospital immediately.

Erica continues to drive, leaving the runner. The runner catches his breath and is fine.

Erica continues to drive, leaving the runner. The runner faints and collapses.

How morally acceptable was Erica's behavior?

How much punishment does Erica deserve for her behavior?

How much blame does Erica deserve?

Veterinarian/Dog poison

Roberto is a student working at a veterinarian's office. His friend comes to visit him and sees cookies shaped like bones laying out in the worker's lounge. His friend asks if he can eat one.

One of Roberto's coworkers had baked the cookies for her birthday and had put them out for others to enjoy. They are delicious and safe to eat.

One of Roberto's co-workers had put the cookies in the lounge by accident. They are used to put dogs to sleep, so they are poisonous to eat.

Roberto believes that the cookies are safe to eat, since they are out in the workers' lounge where people normally put food to share.

Roberto believes that the cookies are poisonous, because they are the shape of the cookies the veterinarian uses with special chemicals to put dogs to sleep.

Roberto invites his friend to eat a dog bone-shaped cookie. His friend enjoys the cookie and asks for a second one.

Roberto invites his friend to eat a dog bone-shaped cookie. His friend breaks into a sweat after several bites, stops breathing, and needs to be hospitalized.

How morally acceptable was Roberto's behavior?

How much punishment does Roberto deserve for his behavior?

How much blame does Roberto deserve?

Zoo

Leonardo is at the zoo with his nephew. They are watching the dolphin show when the nephew complains that his stomach hurts.

Leonardo’s nephew is really fine. His stomach sometimes hurts when he eats too much junk food like that day, but he usually feels a lot better after an hour or so.

Leonardo’s nephew is really sick. After his recent operation, his doctors had warned that stomach pain could indicate really serious complications.

Leonardo believes that his nephew’s stomach hurts because he ate too much cotton candy and fried dough that afternoon. Leonardo thinks his nephew just needs to walk it off.

Leonardo believes that his nephew’s stomach hurts because of a major operation he’d had several weeks ago. Leonardo thinks that his nephew needs medical attention immediately.

Leonardo takes his nephew to see the monkeys next. His nephew starts feeling better in no time. They end up seeing nearly all the exhibits at the zoo.

Leonardo takes his nephew to see the monkeys next. His nephew starts feeling worse and soon blacks out because of severe internal bleeding.

How morally acceptable was Leonardo’s behavior?

How much punishment does Leonardo deserve for his behavior?

How much blame does Leonardo deserve?

Sushi

Mauro and his colleagues are at a new sushi restaurant close to their office. Mauro happens to know the owner of the restaurant through a mutual friend.

The restaurant owner takes great care to ensure the freshness of all the fish prepared sushi-style. Everything exceeds health standards. The tuna is a specialty.

The restaurant owner has purchased some of his fish at cheap but disreputable fish markets to save money. Some batches of fish, usually the tuna, have parasites.

Mauro believes the tuna is especially excellent, after hearing his friend rave about it. Mauro thinks anyone who likes tuna should order the tuna here.

Mauro believes that the tuna isn’t very fresh after hearing his friend complain about it. Mauro thinks everyone should avoid the tuna in case of parasites.

Mauro recommends the tuna to his colleagues at the table. One of his colleagues orders the tuna and ends up finding it quite good.

Mauro recommends the tuna to his colleagues at the table. One of his colleagues orders the tuna and ends up getting a nasty strain of parasites.

How morally acceptable was Mauro’s behavior?

How much punishment does Mauro deserve for his behavior?

How much blame does Mauro deserve?

Cayo/Monkeys

Elisabetta and her assistant are studying primate cognition on an island off Puerto Rico. The monkeys there roam around freely. The assistant is in charge of handling the monkeys.

The monkeys carry some diseases that can be transmitted to birds, but they don’t carry any diseases that could be transmitted to humans.

The monkeys carry some rare diseases that can be transmitted to humans by bites or even minor scratches.

Elisabetta talks to the knowledgeable natives on the island at length about the monkeys. She believes that the monkeys are free of diseases that may be dangerous to humans.

Elisabetta talks to the knowledgeable natives on the island at length about the monkeys. She believes that the monkeys carry many diseases that may be dangerous to humans.

Elisabetta tells her research assistant to not bother with protective gear. Her research assistant takes her advice. She leaves the island totally healthy and disease-free a month later.

Elisabetta tells her research assistant to not bother with protective gear. Her research assistant takes her advice. She gets scratched by a monkey and contracts a strain of Herpes.

How morally acceptable was Elisabetta’s behavior?

How much punishment does Elisabetta deserve for her behavior?

How much blame does Elisabetta deserve?

Wet floor

Maria is at the airport. She sees an elderly man with a cane clumsily running down the terminal. The floor in the terminal has just been mopped, and there is a “Caution” sign.

The man sees the caution sign right in front of him, so he’ll slow down when he reaches the wet part of the floor.

The man completely misses the caution sign, so he’ll continue running clumsily in order to make his flight in time.

Maria believes that the man sees the sign since he is staring right at it. She thinks that he’ll slow down when he reaches the wet part of the floor.

Maria believes that the man has not seen the caution sign himself since he is not looking in front of him. She thinks that he’ll slip and get hurt.

Maria does not shout for the elderly man to slow down. The man slows down and walks safely across the terminal.

Maria does not shout for the elderly man to slow down. The man slips on the wet floor and falls hard, breaking his hip.

How morally acceptable was Maria’s behavior?

How much punishment does Maria deserve for her behavior?

How much blame does Maria deserve?

Lab

Dan is giving a visitor a tour of a lab. Before visitors enter the testing room, all test tubes containing disease antigens must be contained in a chamber by flipping a switch. A repairman has just come to fix the switch, which had been broken.

The switch has been successfully repaired, so the test tubes are quite safely contained. Thus, anybody who enters the room will be safe and unexposed.

The switch is still broken, so the test tubes are out in the open. Thus, anybody who enters the room will contract certain disease.

Dan believes that the switch has been fixed after a conversation with the repairman, so he believes it is safe for the visitor to enter.

Dan believes that the switch is still broken after a conversation with the repairman, so he believes it is not safe for the visitor to enter.

Dan tells the visitor to enter the testing room. The visitor does not contract any disease and is fine.

Dan tells the visitor to enter the testing room. The visitor contracts an infectious disease.

How morally acceptable was Dan's behavior?

How much punishment does Dan deserve for his behavior?

How much blame does Dan deserve?

Vitamin

Claudio is instructed by a doctor to give his senile wife pills for her heart disease. The doctor says that she must not intake vitamin K within an hour in order to safely take the pills. One day his wife tries a new kind of fruit.

The new kind of fruit does not have Vitamin K, so it is safe for Claudio's wife to take the pills right away.

The new kind of fruit is high in Vitamin K, so it is dangerous for Claudio's wife to take the pills right away.

Claudio does his research and believes that the new kind of fruit does not have vitamin K, and that it is safe to give her the pills.

Claudio does his research and believes that the new kind of fruit is high in vitamin K, and that it is not safe to give her the pills.

Claudio gives his wife the pills right away. His wife is just fine.

Claudio gives his wife the pills right away. His wife has a heart attack.

How morally acceptable was Claudio's behavior?

How much punishment does Claudio deserve for his behavior?

How much blame does Claudio deserve?

Airport

Giuseppe works at airport. He is in charge of ensuring that the runways are clear of debris that could damage planes during takeoff. A plane is about to take off on a distant runway.

The distant runway has been completely cleared. It is ready for the plane to use for takeoff.

The distant runway has not yet been cleared. There is potentially damaging debris on it.

Giuseppe checks with his supervisor and believes that the runway has already been cleared of any debris. He thinks that it's safe for the plane to taxi.

Giuseppe checks with his supervisor and believes that the runway has not yet been cleared of debris. He thinks that it may be unsafe for the plane to taxi.

Giuseppe gives the OK for the takeoff. The plane takes off on time from the distant runway. The takeoff is safe and without incident.

Giuseppe gives the OK for the takeoff. The plane takes off and suffers serious damage from some debris. Three passengers are seriously hurt.

How morally acceptable was Giuseppe's behavior?

How much punishment does Giuseppe deserve for his behavior?

How much blame does Giuseppe deserve?

Chairlift

Sara is on winter break. She is running a chair lift at a ski lodge. She has just taken over after lunch break and is starting her afternoon shift. The first passenger is a child.

This afternoon, the chair lift is functioning perfectly normally and can safely carry passengers of any size up the side of the ski slope.

This afternoon, a problem with the electricity is making the old chairs bounce violently, making the lift dangerous for small passengers.

Sara believes that the ski lift is in good condition and is safe for anyone to ride, as usual. She saw her coworker had sent a group of children on the chairlift before lunch.

Sara believes that the ski lift is in bad condition and is unsafe for children in particular since her supervisor told her that it was malfunctioning before lunch.

Sara starts the chairlift, and the child gets on. The child has a safe ride to the top of the ski slope, skis down the hill, and returns to the chairlift again and again.

Sara starts the chairlift, and the child gets on. About half way through the ride the chairlift makes a massive jolt, dislodging the child who falls in the snow few feet down and sustains severe injuries.

How morally acceptable was Sara's behavior?

How much punishment does Sara deserve for her behavior?

How much blame does Sara deserve?

Bike

Raffaella's classmate wants to borrow her bike to go biking. Raffaella's bike has just come back from the repair shop. The brakes had not been working.

The brakes on Raffaella's bike are working perfectly now, so the bike is safe to ride.

The brakes on Raffaella's bike still aren't working at all, so the bike is dangerous to ride.

The people at the repair shop told Raffaella that the brakes are fully fixed now, and gave her a demo to explain how they were fixed, so she believes the bike is safe.

The people at the repair shop told Raffaella that the brakes are still broken, and gave her a demo to explain why they aren't fixed, so she believes the bike is unsafe.

Raffaella lends the bike to her classmate. Her classmate bikes on a country road and has a wonderful time.

Raffaella lends the bike to her classmate. Her classmate has a nasty accident because she can't brake while biking on a country road and fractures her ribs.

How morally acceptable was Raffaella's behavior?

How much punishment does Raffaella deserve for her behavior?

How much blame does Raffaella deserve?

Safety Town/Fire drill

Silvio is babysitting a preschool boy. His job is to watch the boy at Safety Town, which is a class designed to help children know what to do in case a fire breaks out. Today the children will go into a fake house full of smoke.

The boy has very strong lungs and is also used to second-hand smoke, so he will be comfortable going into the Safety Town smoking house.

The boy has asthma, which makes his lungs close up around smoke, so he will not be able to breathe if he goes into the Safety Town smoking house.

Silvio believes that the boy is familiar with smoky environments, since both of the boy's parents are smokers. So, Silvio thinks the boy should be just fine participating at Safety Town.

Silvio believes that the boy has a bad reaction to smoky environments, since the boy's parents told Silvio that he has asthma. So, Silvio thinks that it is dangerous for the boy to participate at Safety town.

Silvio watches the boy go into the Safety Town smoking house. The boy comes out smiling because he has learned what to do in case of a fire.

Silvio watches the boy go into the Safety Town smoking house. The boy has a severe asthma attack inside and starts having a seizure.

How morally acceptable was Silvio’s behavior? How much punishment does Silvio deserve for his behavior? How much blame does Silvio deserve?

Parachute

Alice is a new employee at a small sky diving company. Her first customer weighs 100 kg. She has just opened a new batch of parachutes.

The parachutes are very strong and well-made. They will last a lifetime and can be used by safely anyone of any weight.

The parachutes are faulty and should be discarded immediately. They won’t be able to support anyone heavier than 70 kg.

After receiving assurances from her supervisor, Alice believes that the new parachutes are well-made and can bring all her customers to a safe landing.

After receiving warnings from her supervisor, Alice believes that the new parachutes are faulty and cannot bring anyone over 70 kg to a safe landing.

Alice gives the 100 kg customer a new parachute. He uses the new parachute, and it successfully slows his fall. He enjoys a perfect landing.

Alice gives the 100 kg customer a new parachute. He uses the new parachute, and it cannot hold his weight. He hits the ground way too fast and breaks his legs.

How morally acceptable was Alice’s behavior?

How much punishment does Alice deserve for her behavior?

How much blame does Alice deserve?

Sculpture

Paola is taking a class in sculpture. She is assigned to work with a partner to weld together pieces of metal and her partner is holding the other end of the metal being welded.

The metal being welded is a bad conductor of heat so heat travels very slowly along the piece of metal.

The metal being welded is a good conductor of heat so heat travels very fast along the piece of metal.

Paola thinks that the metal is a bad conductor and if she welds a piece of metal that her partner is holding the heat will travel down really slow down the metal and will not burn her partner's hand.

Paola thinks that the metal is a good conductor and if she welds a piece of metal that her partner is holding the heat will travel down the metal fast and burn her partner's hand.

Paola welds the metal, and her partner’s hand is not burned.

Paola welds the metal, and her partner's hand is burned.

How morally acceptable was Paola’s behavior?

How much punishment does Paola deserve for her behavior?

How much blame does Paola deserve?

Dentist

Franco is a dentist filling in the cavity of his patient. He must drill into the patients tooth just above a major nerve.

If the drill is used at a high speed, it will just dig the cavity and will not hit the nerve.

If the drill is used at a high speed, it will hit the nerve and cause excruciating pain.

Franco thinks that if he switches the drill to a higher speed he will dig the cavity without hitting the nerve below.

Franco thinks that if he switches the drill to a higher speed he will not only dig the cavity but also hit the nerve below.

Franco switches the drill to a higher speed, digs the cavity, and does not touch the nerve below.

Franco switches the drill to a higher speed, hits the nerve, and causes the patient excruciating pain.

How morally acceptable was Franco's behavior?

How much punishment does Franco deserve for her behavior?

How much blame does Franco deserve?

Iron

Anna and her little sister are in the bathroom doing makeovers by the sink. Anna had straightened her hair earlier in the day using a straightening iron. The iron is still on the sink.

Anna's mother had turned the button on the iron off 3 h ago, so it is no longer hot and is perfectly safe to touch.

Anna's mother had just used the iron herself 5 min ago, so it is still extremely hot and could cause severe burns.

Because the cord on the iron was unplugged, Anna believes that it is no longer hot and cannot burn her sister.

Because the cord on the iron is still in the socket, Anna believes that it is still hot and could easily burn her sister.

Anna lets her sister continue to play by the sink. Her sister's arm hits the iron, but it is okay because the iron is cold. They have fun continuing their makeovers.

Anna lets her sister continue to play by the sink. Her sister's arm hits the iron, and she gets badly burned. She cries hysterically all the way the emergency room.

How morally acceptable was Anna's behavior?

How much punishment does Anna deserve for her behavior?

How much blame does Anna deserve?

Tree House

Federico finds an abandoned tree house in the woods. He takes a younger buddy there to show him his great discovery.

The tree house is new and has been built with thick heavy wood, so it makes a very safe and fun hangout spot for boys.

The tree house is old and built with now rotting wood, so it is incredibly dangerous to even set foot in.

Because of its newly built appearance, Federico believes that the tree house is sturdy and that it will be a safe place for them to play.

Because of its old dingy appearance, Federico believes that the tree house is weak and that it will be an unsafe place for them to play.

Federico invites his buddy to climb into the tree house. His buddy jumps around and has a great time up in the air.

Federico invites his buddy to climb into the tree house. His buddy breaks one of his legs after falling through broken floorboards.

How morally acceptable was Federico's behavior?

How much punishment does Federico deserve for his behavior?

How much blame does Federico deserve?

Jellyfish/ Ocean

Ilaria and her neighbor are kayaking in a part of the ocean with lots of jellyfish. Ilaria's neighbor asks her if she should go for a swim.

It is perfectly safe to swim in the ocean, because the jellyfish do not sting and are totally harmless.

It is not safe to swim in the ocean, because the jellyfish sting and their stings are really painful.

Because Ilaria read information that said the ocean's jellyfish are harmless, she believes that it is quite safe to swim in the ocean.

Because Ilaria read information that said the ocean's jellyfish are harmful, she believes that it is not safe to swim in the ocean.

Ilaria tells her neighbor to go for a swim. Her neighbor does, enjoys the swim, and is just fine.

Ilaria tells her neighbor to go for a swim. Her neighbor does, gets stung by jellyfish, and experiences a lot of pain due to the sting.

How morally acceptable was Ilaria's behavior?

How much punishment does Ilaria deserve for her behavior?

How much blame does Ilaria deserve?

Laptop

Claudio is a new computer technician at a store. A customer comes to the store to get her laptop checked out. The laptop gets very hot after only 10 min of work.

The laptop is within the normal range in terms of heating up with use. It is totally safe to use on one's desk and lap.

The laptop is malfunctioning and heats up much more than normal. It is dangerous to use, especially on one's lap.

Claudio checks with his boss and comes back believing it is very normal for all laptops to heat up during use.

Claudio checks with his boss and comes back believing that many laptops will catch on fire if they overheat and cause burns.

Claudio returns the laptop to the customer. The customer goes home, and uses her laptop to get a lot of important work done.

Claudio returns the laptop to the customer. The customer goes home, and her laptop catches on fire. She suffers painful second-degree burns.

How morally acceptable was Claudio's behavior?

How much punishment does Claudio deserve for his behavior?

How much blame does Claudio deserve?
